# Utilizing bioinformatics and machine learning to identify CXCR4 gene-related therapeutic targets in diabetic foot ulcers

**DOI:** 10.3389/fendo.2025.1520845

**Published:** 2025-02-07

**Authors:** Hengyan Zhang, Ye Zhou, Heguo Yan, Changxing Huang, Licong Yang, Yangwen Liu

**Affiliations:** ^1^ Department of Dermatology, Zhaotong Hospital of Traditional Chinese Medicine, Zhaotong, Yunnan, China; ^2^ Department of Endocrinology, Zhaotong Hospital of Traditional Chinese Medicine, Zhaotong, Yunnan, China

**Keywords:** diabetic foot ulcer, CXCR4, bioinformatics, machine learning, therapeutic target, wound healing

## Abstract

**Background:**

Diabetic foot ulcers (DFUs) are a serious complication of diabetes mellitus that manifests as chronic, non-healing wounds that have a significant impact on patients quality of life. Identifying key molecular targets associated with DFUs could help develop targeted therapies to promote wound healing and prevent further complications. The CXCR4 gene is known to play a key role in cell migration, immunology response, and tissue repair, and thus may be an important target for DFU treatment.

**Methods:**

We used the GEO database (Gene Expression Omnibus database) to obtain DFU-related gene expression data, identified differentially expressed genes (DEGs), and performed enrichment analysis to reveal the related biological pathways. Meanwhile, protein-protein interaction (PPI) networks were constructed using STRING to identify core genes. Feature selection methods such as LASSO, SVM-RFE and random forest algorithm were applied to localize possible therapeutic target genes. Finally, We analyzed the molecular pathways of CXCR4 in DFUs by Gene set enrichment analysis (GSEA).

**Results:**

We identified a total of 751 differential genes, of which 409 genes were significantly upregulated and 342 genes were downregulated in diabetic foot ulcer tissues. Functional enrichment analysis showed that these genes were mainly involved in pathways such as oxidative phosphorylation, phagosome, synaptic vesicle cycle, and pathways of neurodegeneration. We integrated the genes screened by three machine learning models (LASSO, SVM, and Random Forest), and CXCR4 was identified as a key gene with potential therapeutic value in DFUs. Gene set enrichment analysis (GSEA) showed that CXCR4 was closely associated with pathways related to immunology regulation and tissue repair.

**Conclusion:**

The findings suggest that CXCR4 and its related pathways play an important role in the pathogenesis of DFUs, providing a new perspective on targeted therapy for wound healing in diabetic patients. Further validation of the role of CXCR4 is expected to establish it as an important target in DFU management.

## 1 Introduction

Diabetic foot ulcers (DFUs) are one of the most common and highly disabling complications of diabetes, characterized by persistent foot ulcers with high rates of infection and risk of amputation, posing a major challenge to patient quality of life and public health systems (1). According to data projections, by 2030, the global diabetic population is estimated to reach approximately 439 million (2). Among diabetic patients, around 30% will develop foot ulcers during their lifetime (3), with a portion of these patients at risk of amputation due to worsening ulcers. Studies indicate that by 2050, one in three Americans will have diabetes, and up to 34% of diabetic patients will develop diabetic foot ulcers (DFUs) in their lifetime (4).

DFUs are a severe complication for adult diabetic patients (5), with approximately 19%-34% experiencing foot ulcers during their lifetime, and this risk increases with patient age and healthcare complexity (6). DFUs can lead to serious outcomes such as infection, amputation, and death, with a recurrence rate of 65% within 3-5 years (7), an amputation rate of 20%, and a 5-year mortality rate as high as 50%-70% (8). Despite advancements in multidisciplinary prevention and early screening, amputation rates have increased in certain regions, especially affecting younger individuals and minority groups, highlighting disparities and inequalities in DFU management (9). Additionally, diabetic patients often have weakened immune function and reduced infection resistance (10), further increasing the risk of DFU-related infections (11). Among these mechanisms, persistent inflammatory responses and impaired tissue repair (12) are considered key drivers in the progression of DFUs. Recent studies have indicated that the CXCR4 gene plays an important role in processes such as cell migration, inflammatory regulation, and tissue repair (13), and that aberrant expression of CXCR4 is regarded as a driver of disease progression in multiple chronic conditions (14, 15).

CXCR4 is expressed in various cell types (16) and regulates cell migration, proliferation, and inflammatory responses via its ligand CXCL12 (17). Research has shown that CXCR4 is aberrantly expressed in chronic wounds like DFUs, potentially leading to delayed ulcer healing and increased infection rates. For instance, studies have indicated that CXCR4 upregulation in DFUs may accelerate angiogenesis and wound healing by promoting the expression of VEGF and SDF-1α, thereby improving recovery in DFU patients (18). These findings suggest that CXCR4 plays a crucial regulatory role in the chronic inflammatory response and immune imbalance seen in DFUs, making it a potential therapeutic target.

With rapid advancements in genomics and molecular biology, bioinformatics and machine learning have become essential in studying disease mechanisms. In recent years, obtaining high-throughput gene expression data from public databases (e.g., GEO and TCGA) and conducting differential gene expression analysis (19) have become effective ways to explore the molecular mechanisms of complex diseases like DFUs. Additionally, protein-protein interaction (PPI) networks (20) and enrichment analysis (21) can systematically reveal key genes and pathways related to DFUs. In DFU research, feature selection algorithms based on machine learning (e.g., LASSO, SVM-RFE, and Random Forest) effectively screen biomarkers with diagnostic or therapeutic potential, providing data support for clinical interventions. Bioinformatics (22) and machine learning (23) have become essential tools in understanding disease mechanisms, especially for complex diseases like Diabetic Foot Ulcers (DFUs). Bioinformatics helps in identifying disease-related genes (24) through genomic, transcriptomic, and proteomic data analysis, while machine learning models can predict disease progression and therapeutic responses. These approaches are increasingly used in personalized medicine to identify novel biomarkers and therapeutic targets.

This study aims to systematically analyze the role of the CXCR4 gene in DFUs by integrating bioinformatics and machine learning techniques. We obtained DFU-related gene expression data from the GEO database and identified DFU-associated key genes and their signaling pathways through differential gene analysis, PPI network construction, and enrichment analysis. Additionally, various machine learning algorithms were applied to screen potential therapeutic targets, providing a scientific basis for precision treatment of DFUs.

## 2 Methods

### 2.1 Data acquisition and preprocessing

This study utilized the diabetic foot ulcer (DFU)-associated gene expression dataset GSE165816 (platform GPL24676) from the NCBI GEO database. This dataset includes 18 samples from healthy non-diabetic subjects, 12 samples from diabetic subjects without DFU, and 24 samples from subjects with healing or non-healing DFU. To ensure data consistency and minimize noise, we applied several preprocessing steps. First, gene expression data were standardized using the NormalizeBetweenArray function in R to correct for technical biases. Next, batch effects were corrected using the ComBat method (25), which adjusted for confounding variables, ensuring that observed differences reflected biological variation rather than technical artifacts. For differential gene expression analysis, we employed the Limma package in R to identify differentially expressed genes (DEGs). A P-value < 0.05, |LogFC| > 1, and an adjusted P-value < 0.05 were set as the criteria for selecting significant DEGs. These DEGs were then used for functional enrichment and pathway analysis. Notably, during the data processing and analysis, through appropriate data cleaning, augmentation, and restructuring, we constructed 105 analytical samples from the initial 54 samples. This process included optimizing technical replicates, dataset integration, and preprocessing steps to ensure data quality and the reliability of the analysis.

Regarding sample grouping, we classified the data into two groups: the “DFU group” and the “Control group”. The “DFU group” consists of all samples from DFU patients, including skin samples from both healing and non-healing DFU patients, as well as peripheral blood mononuclear cells (PBMCs) from DFU patients. The “Control group” encompasses samples from healthy controls and diabetic patients without DFU, including skin samples from diabetic patients, as well as skin and PBMC samples from healthy non-diabetic individuals. Although the dataset contains samples from diabetic patients without DFU, we did not further categorize this group, as the dataset does not provide a detailed definition for this subgroup. Moreover, the “Control group” is sufficiently representative for our analysis. Therefore, the focus of this study is to compare DFU patients with healthy controls, rather than further subdividing the diabetic patients without DFU. All sample groupings were based on the original classifications in the GSE165816 dataset, ensuring consistency and traceability of the data.

### 2.2 Functional enrichment analysis

In order to deeply resolve the functions of the screened genes and their roles in key biological processes, recognized bioinformatics tools and databases were used in this study. We utilized gene ontology (GO) analysis and Kyoto Encyclopedia of Genes and Genomes (KEGG) pathway enrichment analysis to identify the key roles of these genes in biological functions and signaling pathways. The GO analysis systematically categorized and annotated the genes according to biological processes (BP), molecular functions (MF), and cellular components (CC), which helped to reveal the important biological processes and molecular functions of the genes in the pathogenesis of diabetic foot ulcer. Meanwhile, KEGG pathway analysis revealed gene interactions in specific cellular processes and molecular cascade reactions, providing clues for identifying potential regulatory imbalances and therapeutic targets for the disease.

### 2.3 Protein interaction network analysis

To deeply explore the protein interaction network (PPI) of the screened genes, this study constructed a high-quality PPI network with the help of advanced bioinformatics tools and databases. We used the STRING database (https://cn.string-db.org/), a wide-coverage and finely organized resource of protein interaction information, and set a confidence threshold of 0.9 to ensure that the network contains only significant interactions with high confidence.

For network visualization, we used Cytoscape 3.8.1, a widely used platform for network data visualization, whose intuitive interface and powerful functional support enabled us to create clear PPI network diagrams, thus helping to reveal the complex interrelationships among genes. In addition, to identify core genes that play important regulatory roles in PPI networks, we used the cytoHubba plug-in for Cytoscape. By computationally analyzing the network structure with cytoHubba, we successfully identified multiple genes that play key roles in network integrity and function.

### 2.4 Feature screening and diagnostic biomarker identification

In this study, three feature selection methods—LASSO, SVM-RFE, and Random Forest (RF)—were employed to identify key genes as potential diagnostic biomarkers for diabetic foot ulcers. LASSO takes as input the gene expression matrix and disease labels. By applying L1 regularization, LASSO shrinks less relevant gene coefficients to zero, leaving only the most predictive genes. The penalty parameters for LASSO were optimized using ten-fold cross-validation to improve model accuracy and robustness. Similarly, SVM-RFE also uses gene expression data and disease labels as input. This method recursively eliminates the least important features, ranking the remaining genes based on their contribution to the classification. Before five-fold cross-validation, the top 30 key genes were selected as the most representative genes in the dataset. The Random Forest method, in contrast, uses only the gene expression data as input. It constructs multiple decision trees to reduce overfitting and improve the model’s generalization ability. The output is a ranking of genes based on their contribution to model performance. Finally, an intersection analysis of the genes selected by all three methods was conducted to identify the most representative gene set. The diagnostic accuracy of these genes was evaluated using the area under the curve (AUC) of the receiver operating characteristic (ROC) curve.

### 2.5 Gene set enrichment analysis

Gene set enrichment analysis (GSEA) was performed on the identified genes using gene sets from the Molecular Signatures Database (MSigDB), focusing on pathways related to immune regulation and tissue repair. GSEA is a statistical method that evaluates whether a predefined gene set exhibits consistent and significant differences between two biological conditions. In our analysis, we calculated the normalized enrichment score (NES) for each gene set by performing 1,000 permutations. To assess the statistical significance of the KEGG pathways, we set the false discovery rate (FDR) threshold at <0.05.

## 3 Results

### 3.1 Differential expression analysis

**Figure 1:**
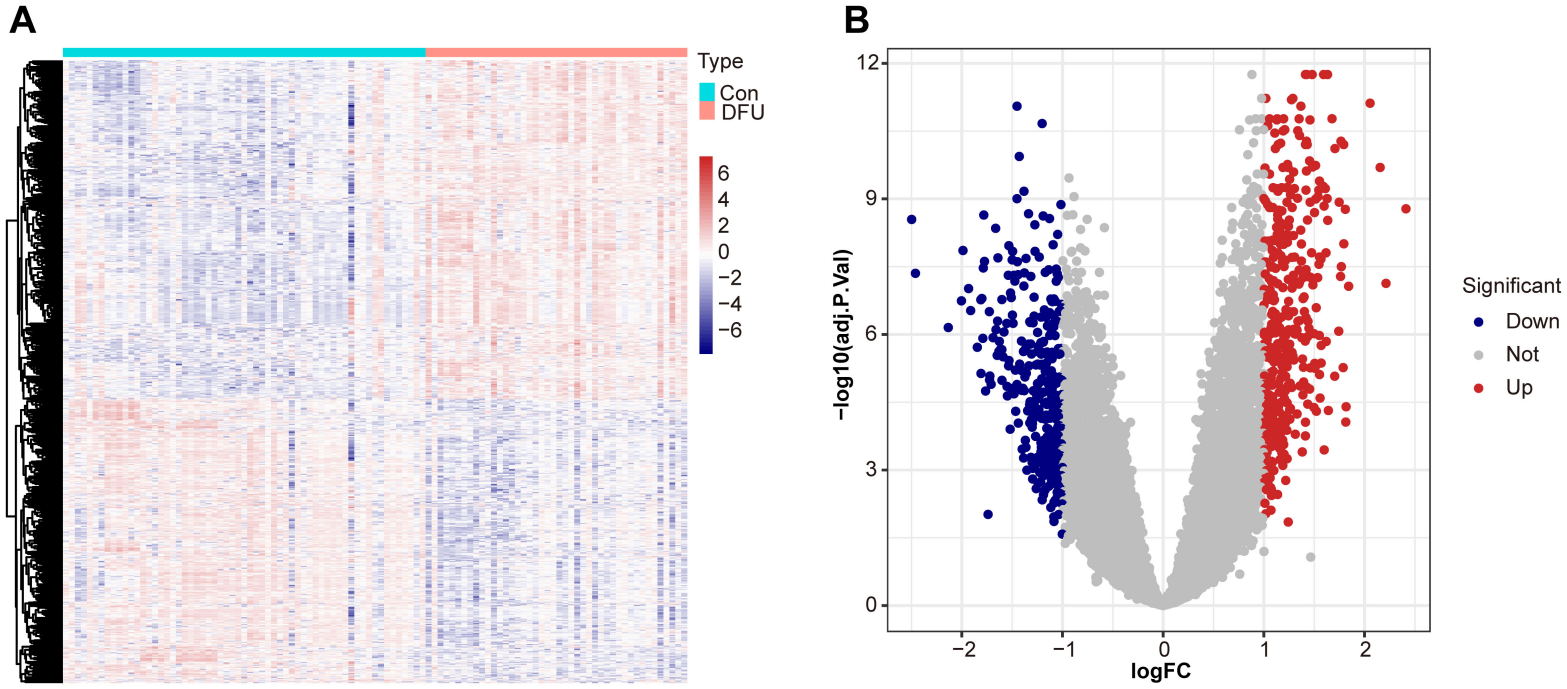
Differential expression analysis between diabetic foot ulcer (DFU) tissues and control samples. **(A)** Heat map showcases the differentially expressed genes (DEGs). **(B)** Volcano plots of DEGs distribution based on dataset GSE165816, with upregulated genes shown in red and downregulated genes in blue. Statistical significance is indicated by p-values and fold changes (threshold: p < 0.05).

In order to describe the results in **Figure 1** in detail, we performed a comprehensive differential expression analysis, revealing significant gene expression differences between diabetic foot ulcer tissues and control tissues. Using the “limma” package in R, we accurately compared the gene expression profiles of the two groups, and identified a variety of genes that were significantly up- or down-regulated in the ulcerated tissues. Using hierarchical cluster analysis, we constructed a heat map show in **Figure 1A** to help reveal the intrinsic relationship between these differentially expressed genes in terms of their expression patterns and identify clusters of similarly expressed genes. The results showed that we identified a total of 751 differentially expressed genes, of which 409 genes were significantly upregulated and 342 genes were downregulated in diabetic foot ulcer tissues. These genes have significant effects in several biological processes and molecular functions, constituting a molecular network closely related to ulcer formation and its progression. **Figure 1B** demonstrates the expression differences between up- and down-regulated genes in a visual way, which provides important clues to unravel the pathological mechanisms of diabetic foot ulcers.

### 3.2 Gene set enrichment analysis

**Figure 2:**
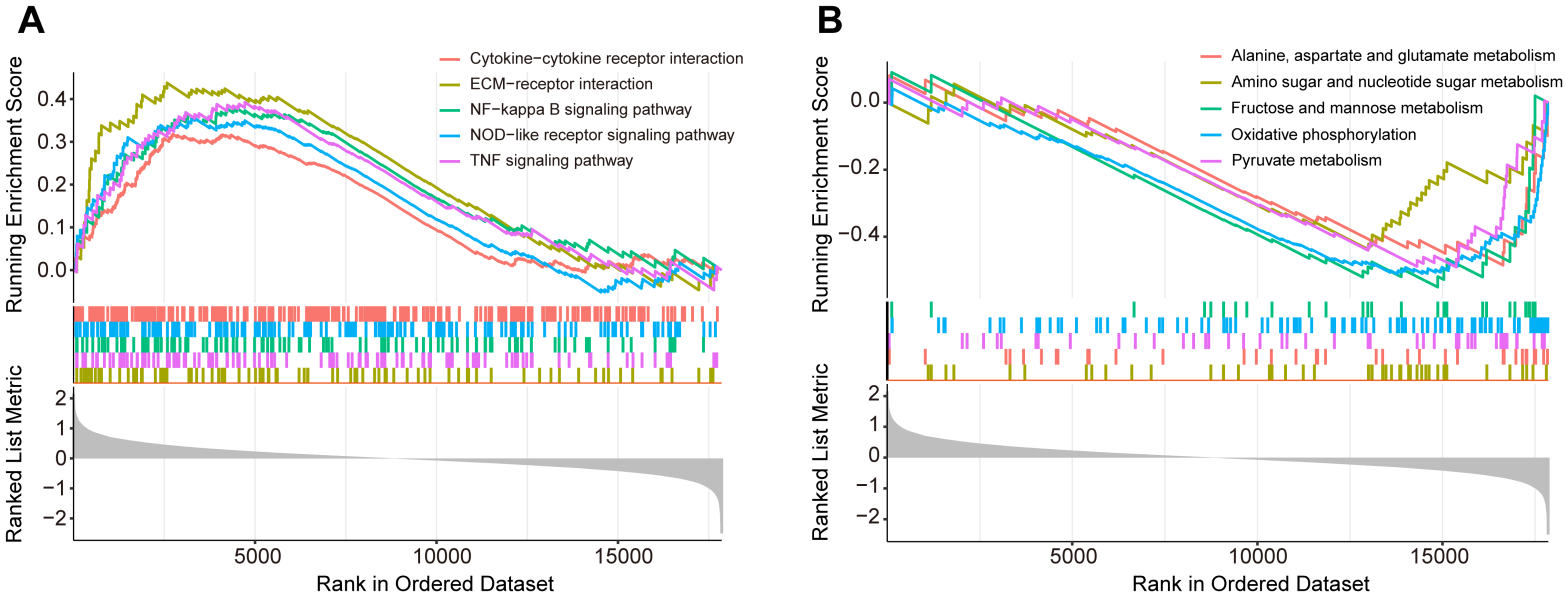
Gene set enrichment analysis (GSEA) of the GSE165816 dataset to identify key biological pathways associated with diabetic foot ulcers (DFU). **(A)** Analysis of the five upregulated pathways of interest. **(B)** Analysis of the five downregulated pathways of interest.

To explore the underlying molecular mechanisms in diabetic foot ulcers, we performed gene set enrichment analysis (GSEA) on the GSE165816 dataset, aiming to revealed significantly enriched processes closely associated with DFU and identify key biological pathways. Further analysis showed that multiple upregulated or downregulated pathways were closely associated with the development of diabetic foot ulcers. The five upregulated pathways of interest demonstrated in **Figure 2A**, included pathways of NF-kappa B signaling pathway, NOD-like receptor signaling pathway, TNF signaling pathway, extracellular matrix-receptor interaction, and cytokine−cytokine receptor interaction. These pathways showed significant upregulation in diabetic foot ulcers, reflecting the important molecular roles of inflammation and immunology regulation in the disease, and provided new ideas for possible targeted therapies. Meanwhile, the five downregulated pathways of interest demonstrated in **Figure 2B** reveals significant decreases in several metabolic and cell function-related pathways, including oxidative phosphorylation, amino acid metabolism and carbohydrate metabolism. The downregulation of these pathways may be associated with the impaired metabolic capacity and reduced antioxidant capacity of ulcerated tissues, which provides further basis for our understanding of the pathophysiological mechanisms of diabetic foot ulcers.

### 3.3 Differential gene-based functional enrichment analysis

**Figure 3:**
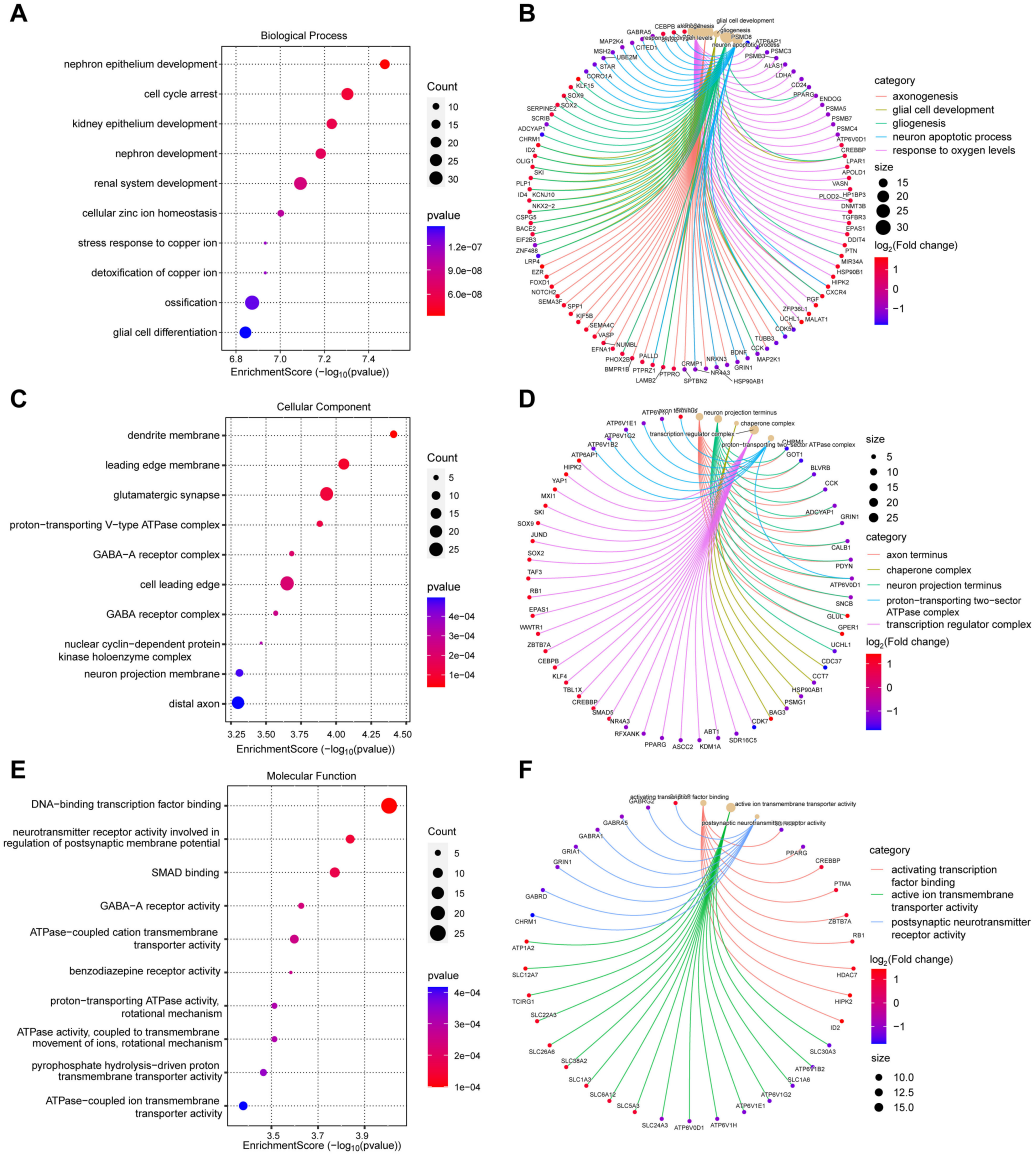
Gene Ontology (GO) analysis of differentially expressed genes (DEGs) in diabetic foot ulcers (DFU). **(A, B)** Analysis of the significantly enriched biological processes. **(C, D)** Analysis of the significantly enriched cellular components. **(E, F)** Analysis of the significantly enriched molecular functions of the DEGs.

To explore the underlying molecular mechanisms of diabetic foot ulcers, we performed comprehensive Gene Ontology (GO) analysis and Kyoto Encyclopedia of Genes and Genomes (KEGG) analysis of differentially expressed genes (DEGs). These analyses helped us gain a deeper understanding of the associated biological processes (BPs), cellular components (CCs), molecular functions (MFs), and notable signaling pathways, which shed light on the causes of dysregulated gene expression in diabetic foot ulcers from multiple perspectives. First, GO analysis revealed biological processes significantly associated with diabetic foot ulcers. **Figure 3A** exhibits the top ten significantly enriched biological processes, including cell cycle arrest, glial cell differentiation, cellular zinc ion homeostasis, and ossification. **Figure 3B** showcases five significantly enriched BPs of interest to the researchers, such as response to oxygen levels, axonogenesis, glial cell development, gliogenesis, and neuron apoptotic process, which may be associated with peripheral neuropathy in diabetic foot ulcers (DFU).

**Figure 4:**
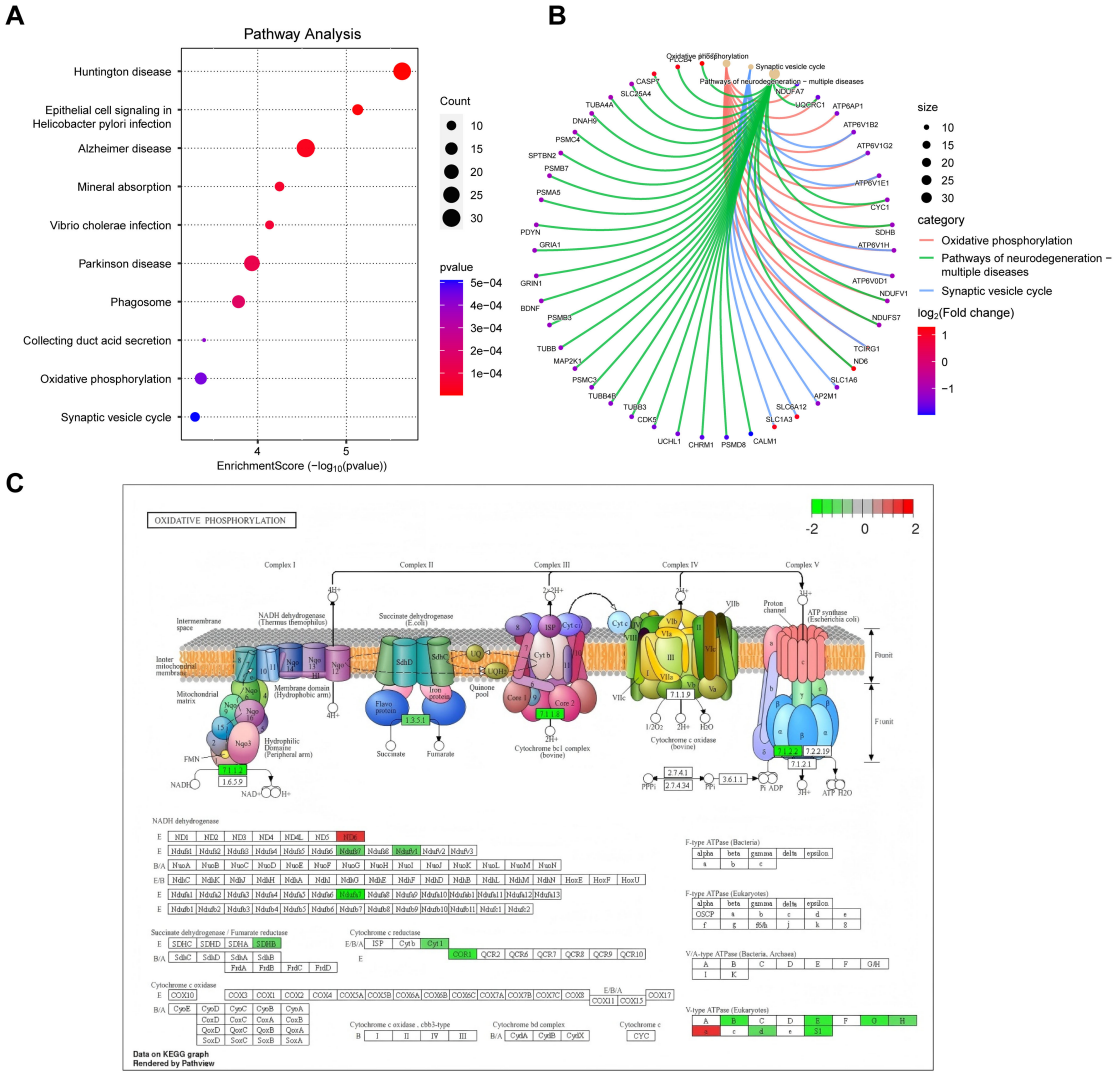
KEGG pathway analysis of DEGs in diabetic foot ulcers (DFU). **(A)** Analysis of the enriched top 10 pathways in DFU. **(B)** Analysis of the enriched pathways of interest. **(C)** An separate pathway diagram.

These findings provide a comprehensive understanding of the molecular pathways and cellular events that contribute to the pathogenesis and progression of DFU. Next, we analyzed the enriched cellular components. The top ten cellular components results, as presented in **Figure 3C**, included leading edge membrane, glutamatergic synapse, distal axon, and some protein complexes. **Figure 3D** reveals some significantly enriched CCs of interest to the researchers, such as axon terminus, transcription regulator complex. In terms of molecular functions, GO analysis revealed diverse functions of DEGs, encompassed activating transcription factor binding, ATPase activity, transmembrane transporter activity, and postsynaptic neurotransmitter receptor activity, etc., as shown in **Figures 3E, F**. These molecular functions indicate the existence of complex signaling and molecular interactions in diabetic foot ulcers, which provide a basis for targeted interventions. Finally, we performed KEGG pathway analysis to reveal biological pathways significantly enriched in diabetic foot ulcers. **Figures 4A, B** showcase the key pathways, including oxidative phosphorylation, phagosome, synaptic vesicle cycle, and pathways of neurodegeneration. The enrichment of these pathways reveals the complex signaling and molecular events that drive diabetic foot ulcers and contributes to a deeper understanding of the mechanisms of disease onset and progression. In addition, we focused on the oxidative phosphorylation pathway, as shown in **Figure 4C**, which provided substantial information about the genes and molecular processes supporting this pathway.

### 3.4 PPI network analysis and core gene identification

**Figure 5:**
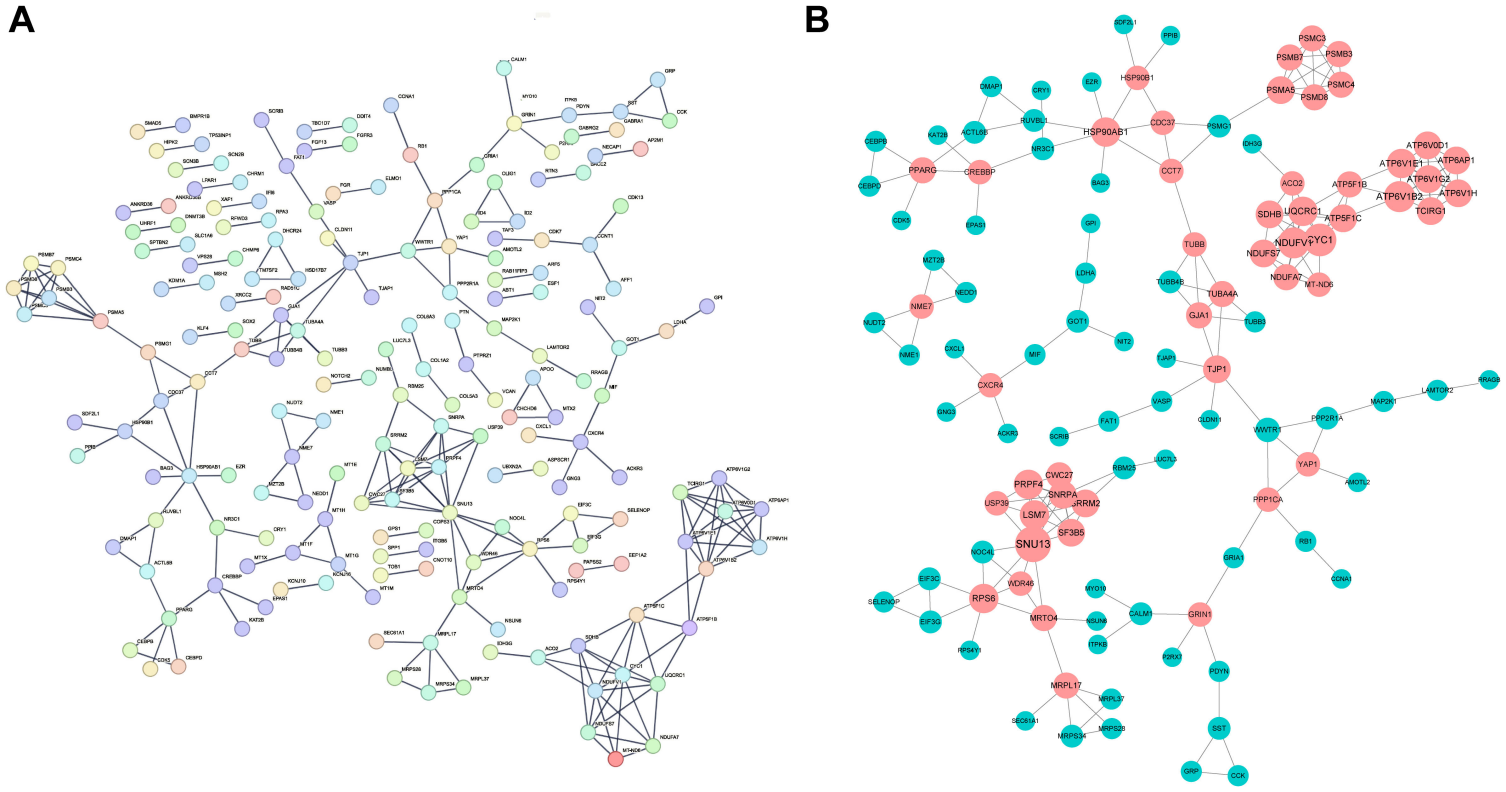
Construction of PPI network and identification of hub genes. **(A)** The Protein-Protein Interaction (PPI) network of the differentially expressed genes (DEGs) identified in DFU tissues. The network illustrates the interactions between key genes that play significant roles in the pathogenesis of DFUs. **(B)** The key hubs within the network were highlighted in red.

By utilizing the STRING database, we constructed protein-protein interaction (PPI) network maps of differentially expressed genes (**Figure 5A**). Subsequently, we imported the PPI network into Cytoscape software for more in-depth network analysis. With the aid of the cytoHubba plug-in in Cytoscape, we identified a core set of genes(n=50) that are closely associated with diabetic foot ulcers and that play important hub roles in the PPI network (**Figure 5B**). These genes within the network were highlighted, particularly those that may influence inflammatory responses and tissue repair processes, which offered insights into potential targets for therapeutic intervention to improve wound healing.

### 3.5 Construction and validation of LASSO, SVM and RF models

**Figure 6:**
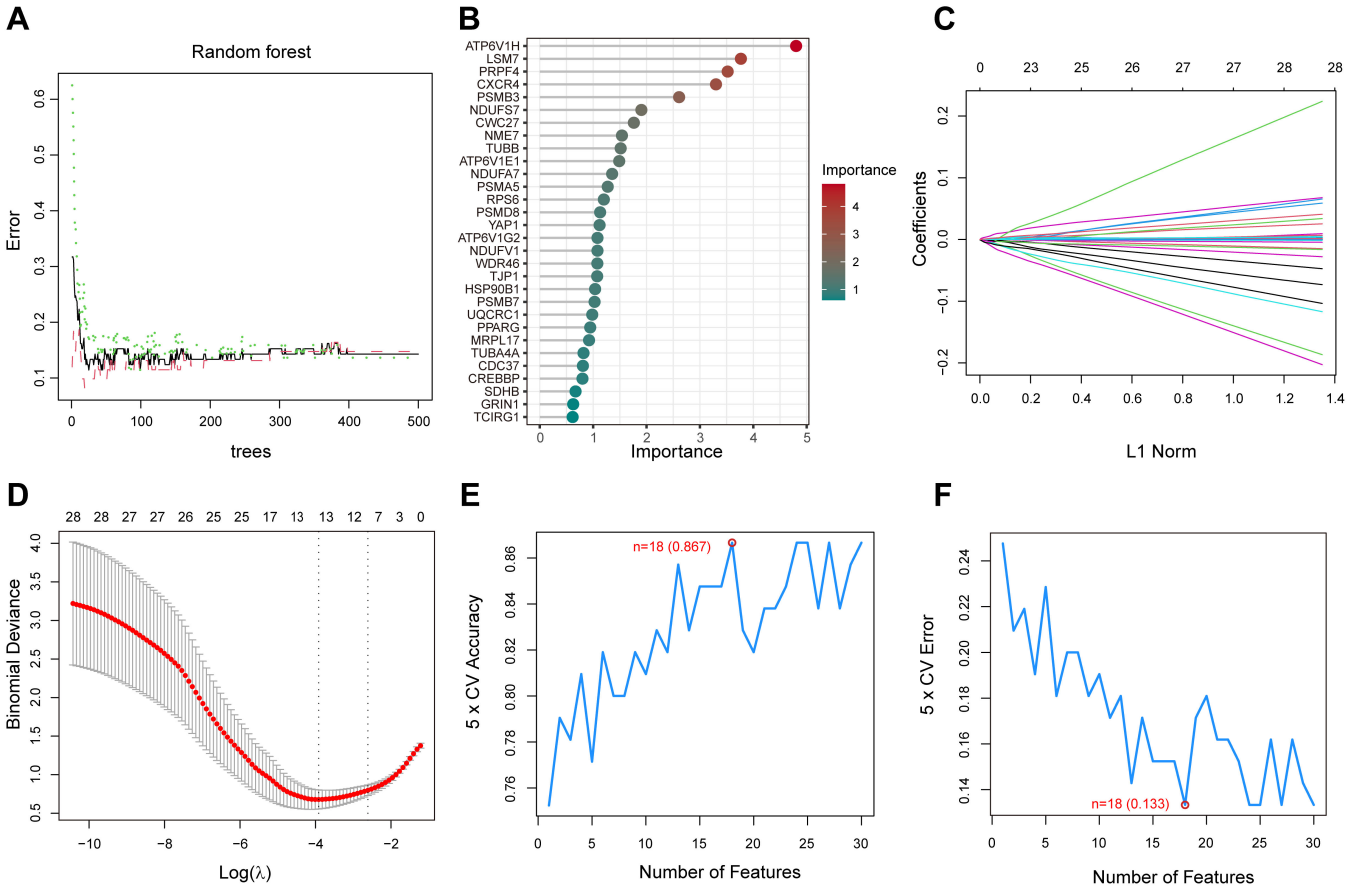
Random Forest (RF), LASSO, and Support Vector Machine (SVM) analysis for gene selection. **(A)** The correlation between the total number of trees in the RF models and the associated error rates. **(B)** An order based on the gene importance scores. **(C)** LASSO analysis of the coefficients. **(D)** The cross-validation process for parameter selection in the LASSO model. **(E)** The average accuracy of the model during the cross-validation process, with the red-marked point indicating the optimal value. **(F)** The average error rate of the model during the cross-validation process, with the red-marked point indicating the optimal value.

We screened key genes from a total of 50 PPI-related DEGs, which closely associated with diabetic foot ulcers by LASSO, support vector machine (SVM) and random forest (RF) models. In the random forest model, gene importance scores were used to visualize the genes identified as the most relevant for predicting diabetic foot ulcer (DFU) occurrence(**Figures 6A, B**). The top 10 scoring genes included ATP6V1H, LSM7, PRPF4, CXCR4, PSMB3, NDUFS7, etc., which were highly weighted in the molecular mechanisms of diabetic foot ulcers, suggesting that they may play an important role in the pathological progression of the disease. The LASSO model further identified a set of genes(n=11) that were significantly associated with diabetic foot ulcers, enabling us to focus on the diagnostic potential of these genes(**Figures 6C, D**). On the other hand, the SVM model eliminated less important features and ranked genes based on their contribution to classification, while the RF model assesses gene importance by evaluating their impact on model performance. By optimizing feature selection, we identified the top 30 genes that could serve as potential biomarkers for DFU prediction and therapeutic targets. Finally, we performed 5-fold cross-validation on these genes to identify the optimal number of features(**Figures 6E, F**).

### 3.6 Identification of feature genes

**Figure 7:**
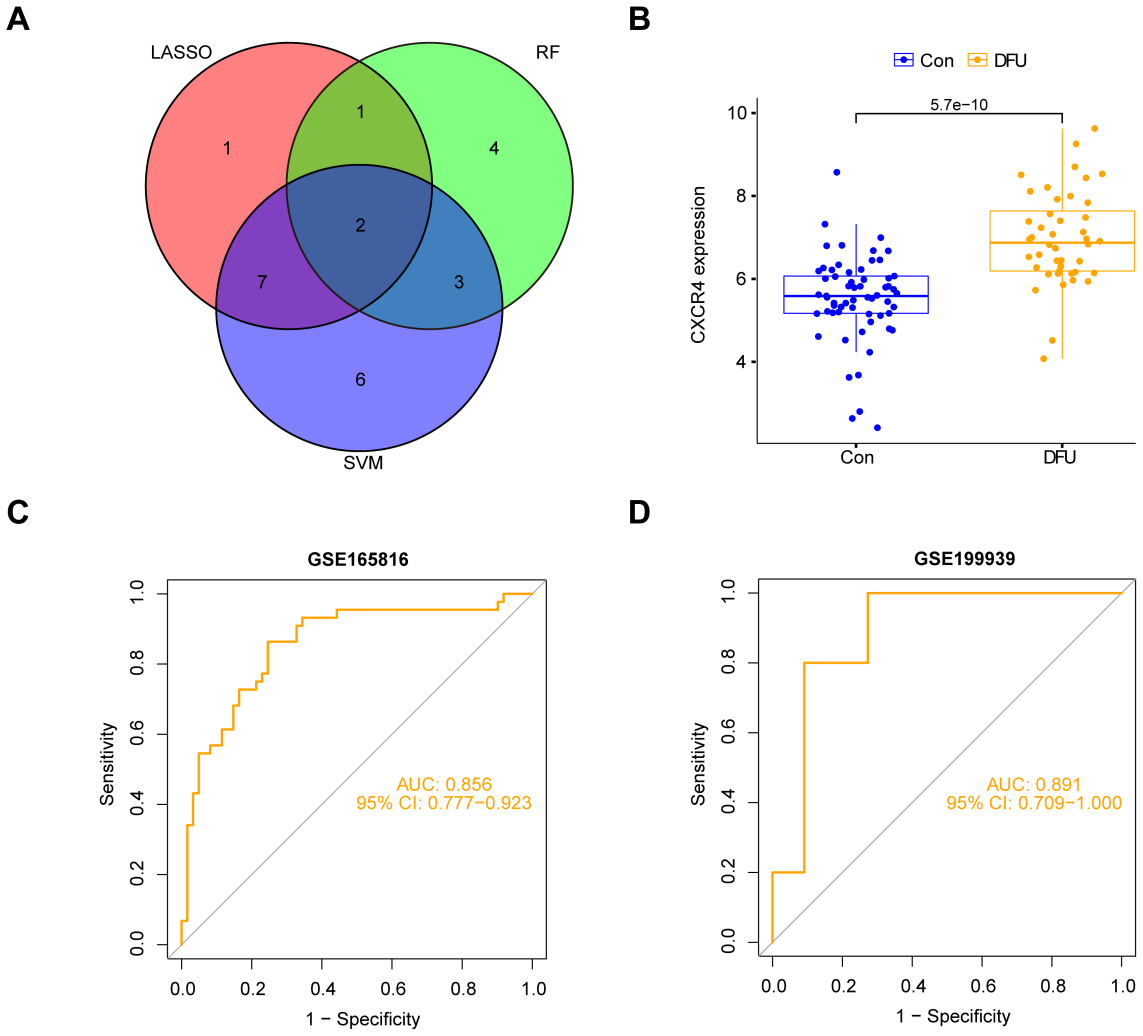
Identification and validation of CXCR4 as a key feature gene for diabetic foot ulcers (DFU). **(A)** Venn diagram shows the intersection of genes from three machine learning models (LASSO, SVM, Random Forest), identifying CXCR4. **(B)** The group of patients with diabetic foot ulcers showed a significant upregulation of CXCR4 expression compared with the control group. **(C)** ROC curve analysis based on dataset GSE165816, confirming CXCR4’s strong diagnostic potential for DFU. **(D)** ROC curve analysis based on dataset GSE199939.

We integrated the genes screened by the three machine learning models (LASSO, SVM, and Random Forest) and took their intersection to identify the diabetic foot ulcer feature genes with the most diagnostic and research value. With this approach, we finally targeted a key gene, CXCR4 (**Figure 7A**). The group of patients with diabetic foot ulcers showed a significant upregulation of CXCR4 expression compared with the control group (**Figure 7B**). Researchers further performed a ROC (subject operating characteristic) curve analysis to assess the diagnostic efficacy of CXCR4. With this analysis, we quantified the predictive performance of CXCR4 and calculated the AUC (area under the curve) value as a measure of diagnostic efficacy. The results of the analysis based on dataset GSE165816 showed that CXCR4 had an AUC value of 0.856(95%CI:0.777-0.923), demonstrating a strong diagnostic ability and indicating its potential as a reliable diagnostic marker for diabetic foot ulcers (**Figure 7C**). Additionally, we performed external validation using the GSE199939 dataset. The results showed that the AUC value for CXCR4 was 0.891(95%CI:0.709-1.000), further confirming its diagnostic value in diabetic foot ulcers(**Figure 7D**).

### 3.7 Functional enrichment analysis of CXCR4

**Figure 8:**
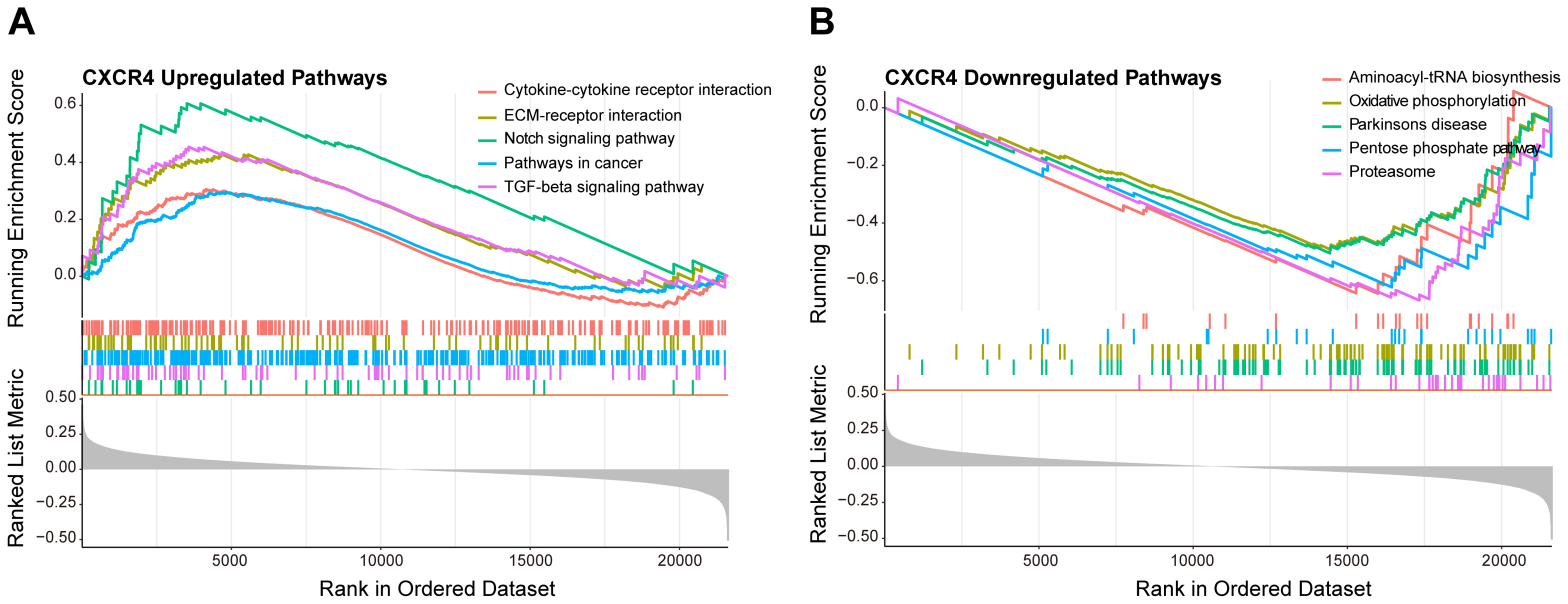
GSEA of CXCR4 in diabetic foot ulcers (DFU). **(A)** A GSEA investigation of the top 5 upregulated pathways. **(B)** An analysis of the top 5 downregulated pathways.

To deeply explore the biological role of CXCR4 in diabetic foot ulcers, we performed gene set enrichment analysis (GSEA) to identify important molecular pathways and biological processes involved in this gene. The GSEA results showed that CXCR4 was significantly involved in several upregulated pathways (**Figure 8A**), including cytokine-cytokine receptor interaction, extracellular matrix-receptor interaction, notch signaling pathway, and TGF-beta signaling pathway. Notably, although CXCR4 is the specific receptor for the chemokine CXCL12, the chemokine signaling pathway was not significantly enriched in this study (p.adj = 0.13 > 0.05). This suggests that CXCR4 may exert its effects through alternative pathways in diabetic foot ulcers (DFU). In addition, CXCR4 was involved in a number of downregulated pathways(**Figure 8B**), mainly including aminoacyl-tRNA biosynthesis, oxidative phosphorylation, pentose phosphate pathway and proteasome. The enrichment of these downregulated pathways indicated an imbalance of metabolic regulation and mitochondrial energy synthesis, as well as suppression of antioxidant responses in diabetic foot ulcers, further revealing its potential function in the disease process. The GSEA results indicated that CXCR4 were significantly enriched in pathways related to immune regulation and tissue repair. This finding underscores the critical role of CXCR4 in the pathogenesis of diabetic foot ulcers (DFUs), particularly in the regulation of inflammation and wound healing. The enrichment of these pathways further supports CXCR4 as a promising therapeutic target for promoting tissue repair in DFUs.

## 4 Discussion

In this study, we investigated the critical role of CXCR4 in diabetic foot ulcers by integrating bioinformatics and machine learning approaches. CXCR4, as a key gene that regulates inflammatory response, tissue repair, and cell migration, has been widely studied and recognized for its role in various chronic diseases. In our study, CXCR4 was found to be significantly upregulated in the tissues of diabetic foot ulcer patients, which is consistent with the high expression of CXCR4 observed in inflammatory diseases in other studies (26). This upregulated expression suggests that CXCR4 may play an important regulatory role in the chronic inflammation (27) and tissue damage repair in diabetic foot ulcers.

Gene set enrichment analysis (GSEA) on the GSE165816 dataset identified key biological pathways closely associated with CXCR4, including cytokine-cytokine receptor interaction, extracellular matrix-receptor interaction, notch signaling pathway, and TGF-beta signaling pathway. These pathways showed significant upregulation in diabetic foot ulcers, reflecting the important molecular roles of inflammation and immunology regulation in the disease. GO and KEGG enrichment analyses (28) further revealed the role of differentially expressed genes (DEGs) in several key pathways, including oxidative phosphorylation, phagosome, synaptic vesicle cycle, and pathways of neurodegeneration. These pathways are closely related to inflammatory responses and also play key roles in tissue repair. Diabetic foot ulcer patients often exhibit slow healing and impaired tissue regeneration (29), and the high expression of CXCR4 may be a crucial factor triggering these symptoms (30). Similar studies have also suggested that the role of CXCR4 in chronic wound healing may be mediated through the regulation of cytokine signaling pathways (31), thereby promoting or inhibiting cell migration and tissue repair (32).

Additionally, the GSEA results showed that low CXCR4 expression was closely associated with the down-regulation of metabolic pathways, such as pentose phosphate pathway (33) and oxidative phosphorylation (34). This finding is consistent with existing studies, indicating that impaired metabolic function in diabetic foot ulcer patients (35) may increase their susceptibility to infection and injury. Impaired metabolic function and antioxidant function are thought to be one of the reasons for the prolonged persistence of diabetic foot ulcers (36), as metabolic imbalance aggravates the condition by reducing tissue resistance to oxidative stress and infection (37).

In terms of machine learning analysis, CXCR4 was identified as a key gene for diabetic foot ulcers through LASSO, SVM, and random forest modeling, which aligns with the multi-model screening strategies used in cancer research and demonstrated good predictive accuracy in this study. The ROC curve analysis for CXCR4 revealed its high predictive accuracy in the early diagnosis of diabetic foot ulcers, suggesting that CXCR4 could become an important biomarker for early detection in clinical practice.

However, this study has some limitations. First, the limited sample size based on transcriptomic data may affect the generalizability of the results. Second, due to data heterogeneity, further validation of the findings is required. Other scholars have also pointed out that results from bioinformatics analyses need further validation in *in vivo* and *in vitro* experiments to clarify the specific mechanisms of CXCR4’s role in diabetic foot ulcers. Future research can experimentally verify the specific functions of CXCR4 in cell migration, inflammation regulation, and metabolic homeostasis, laying the foundation for clinical applications of this gene.

This study confirms the central role of CXCR4 in diabetic foot ulcers, providing a new molecular target and a potential basis for personalized treatment. With more validation studies, CXCR4 is expected to become a new target for the treatment of diabetic foot ulcers, offering more effective intervention strategies for patient healing.

## 5 Conclusion

In this study, we revealed the critical role of CXCR4 in diabetic foot ulcers by bioinformatics and machine learning methods, suggesting its important influence in inflammation regulation, tissue repair and metabolic homeostasis. The up-regulation of CXCR4 may exacerbate the chronic inflammatory response and tissue damage in diabetic foot ulcers, making it a potential diagnostic and therapeutic target. Our findings not only deepen the understanding of the molecular mechanisms of diabetic foot ulcers, but also provide a possible direction for precision intervention in this disease.

## Data Availability

The datasets presented in this study can be found in online repositories. The names of the repository/repositories and accession number(s) can be found in the article/**Supplementary Material**.
