# Supplementary material for: Utilizing bioinformatics and machine learning to identify CXCR4 gene-related therapeutic targets in diabetic foot ulcers
Source: Front Endocrinol (Lausanne). 2025 Feb 7;16:1520845. doi: 10.3389/fendo.2025.1520845 (PMC11842251; doi:10.3389/fendo.2025.1520845)
Supplement: Supplementary file 1 [file DataSheet1.zip › 1520845Supplementary files/07差异基因的GO富集分析/go.d3f24d25e5508754/CC_emapplot.pdf]

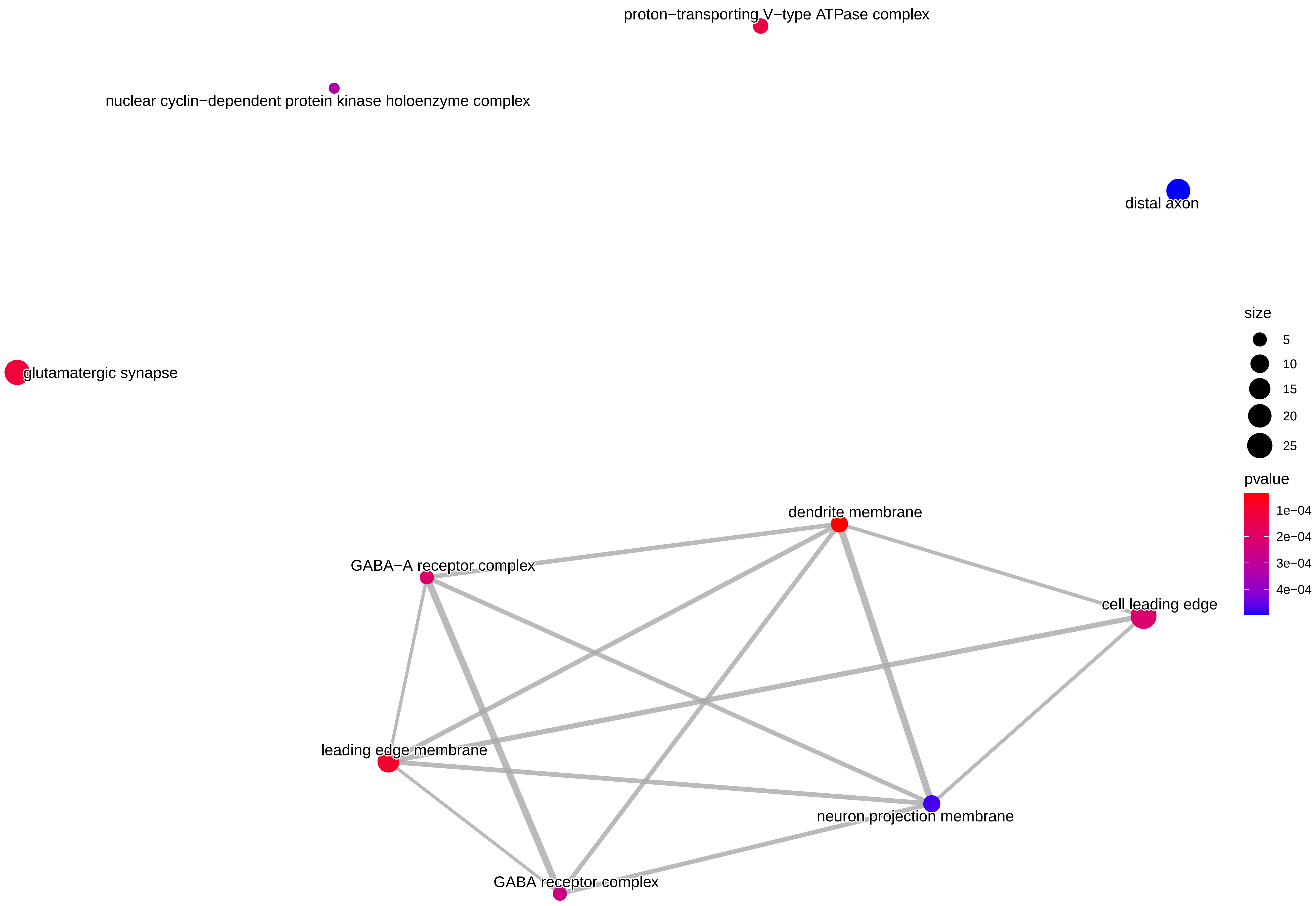
proton−transporting V−type ATPase complex
nuclear cyclin−dependent protein kinase holoenzyme complex
distal axon
glutamatergic synapse
dendrite membrane
GABA−A receptor complex
cell leading edge
leading edge membrane
neuron projection membrane
GABA receptor complex
size
5
10
15
20
25
pvalue
1e−04
2e−04
3e−04
4e−04
