# Supplementary material for: Utilizing bioinformatics and machine learning to identify CXCR4 gene-related therapeutic targets in diabetic foot ulcers
Source: Front Endocrinol (Lausanne). 2025 Feb 7;16:1520845. doi: 10.3389/fendo.2025.1520845 (PMC11842251; doi:10.3389/fendo.2025.1520845)
Supplement: Supplementary file 1 [file DataSheet1.zip › 1520845Supplementary files/08差异基因的KEGG富集分析/pathway.d3f24d25e5508754/Pathway_cnetplot.pdf]

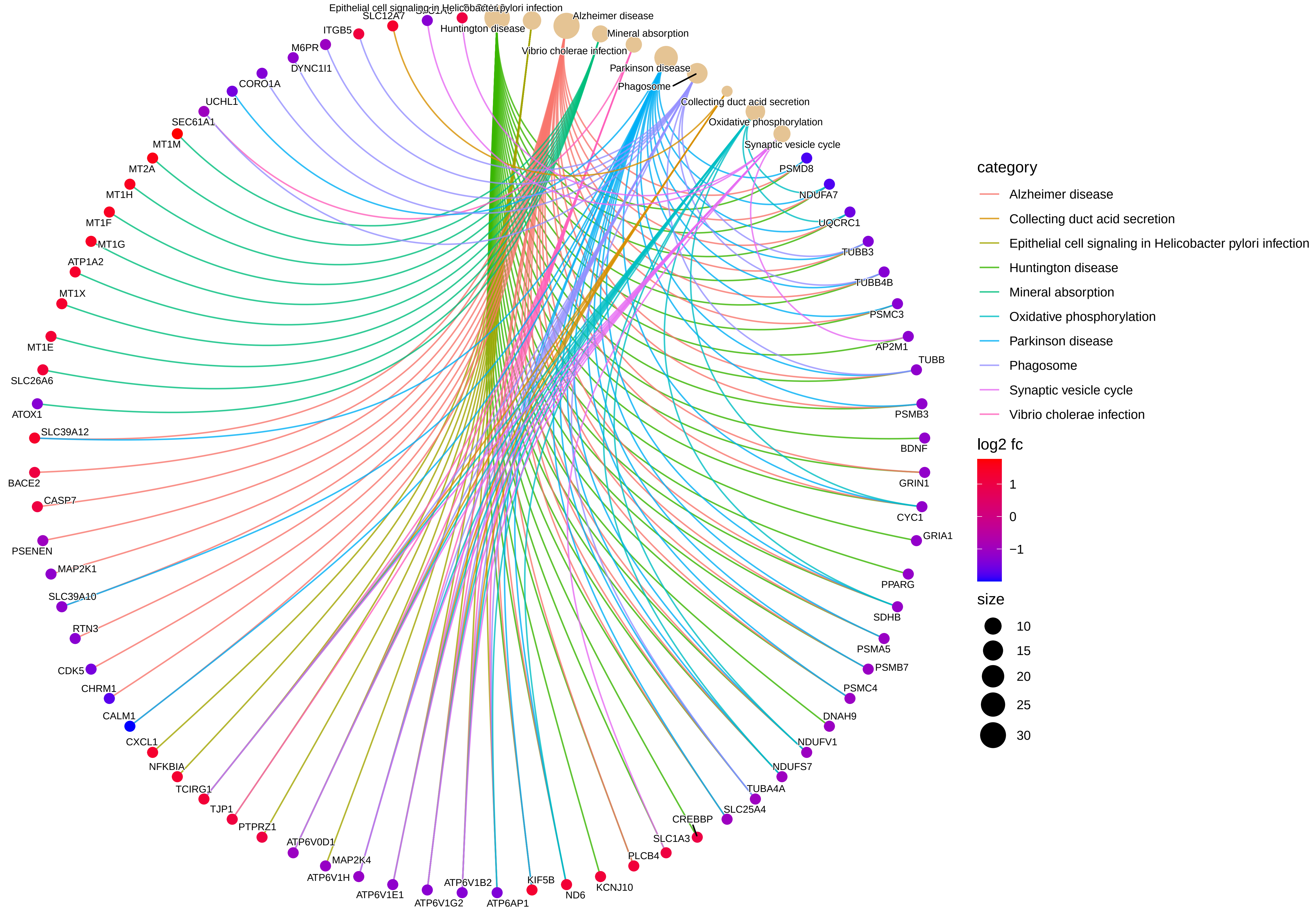

Epithelial cell signaling in Helicobacter pylori infection
Alzheimer disease
Huntington disease
Mineral absorption
Vibrio cholerae infection
Parkinson disease
Phagosome
Collecting duct acid secretion
Oxidative phosphorylation
Synaptic vesicle cycle
SLC12A7
ITGB5
M6PR
DYNC1I1
CORO1A
UCHL1
SEC61A1
MT1M
MT2A
MT1H
MT1F
MT1G
ATP1A2
MT1X
MT1E
SLC26A6
ATOX1
SLC39A12
BACE2
CASP7
PSENEN
MAP2K1
SLC39A10
RTN3
CDK5
CHRM1
CALM1
CXCL1
NFKBIA
TCIRG1
TJP1
PTPRZ1
ATP6V0D1
MAP2K4
ATP6V1H
ATP6V1E1
ATP6V1G2
ATP6V1B2
ATP6AP1
KIF5B
ND6
KCNJ10
PLCB4
SLC1A3
CREBBP
SLC25A4
TUBA4A
NDUFS7
NDUFV1
DNAH9
PSMC4
PSMB7
PSMA5
SDHB
PPARG
GRIA1
CYC1
GRIN1
BDNF
PSMB3
TUBB
AP2M1
PSMC3
TUBB4B
TUBB3
UQCRC1
NDUFA7
PSMD8
category
Alzheimer disease
Collecting duct acid secretion
Epithelial cell signaling in Helicobacter pylori infection
Huntington disease
Mineral absorption
Oxidative phosphorylation
Parkinson disease
Phagosome
Synaptic vesicle cycle
Vibrio cholerae infection
log2 fc
1
0
−1
size
10
15
20
25
30
