# Supplementary figures and images for: Utilizing bioinformatics and machine learning to identify CXCR4 gene-related therapeutic targets in diabetic foot ulcers
Source: Front Endocrinol (Lausanne). 2025 Feb 7;16:1520845. doi: 10.3389/fendo.2025.1520845 (PMC11842251; doi:10.3389/fendo.2025.1520845)

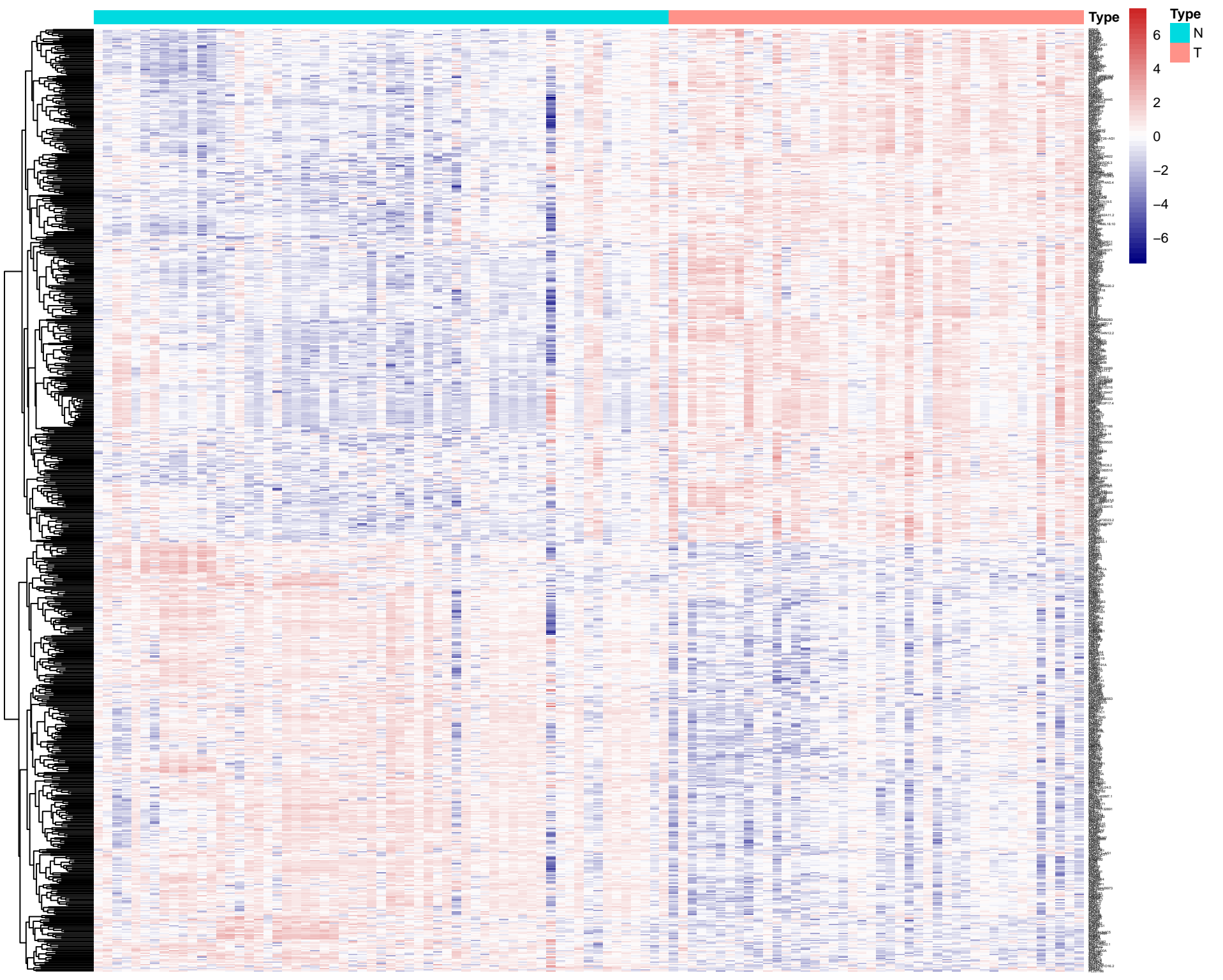

Supplement: Supplementary file 1 [file DataSheet1.zip › 1520845Supplementary files/04差异分析/GEO_heatmap.pdf]

Volcano Plot

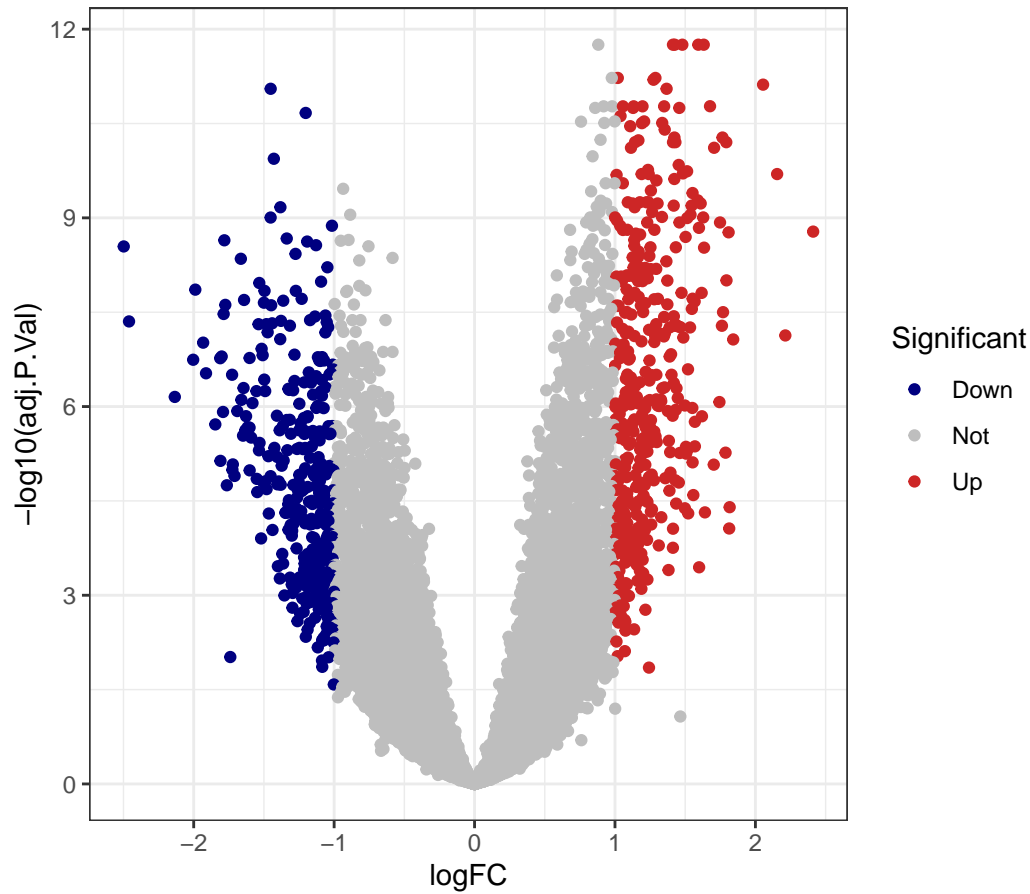

Supplement: Supplementary file 1 [file DataSheet1.zip › 1520845Supplementary files/04差异分析/GEO_vol.pdf]

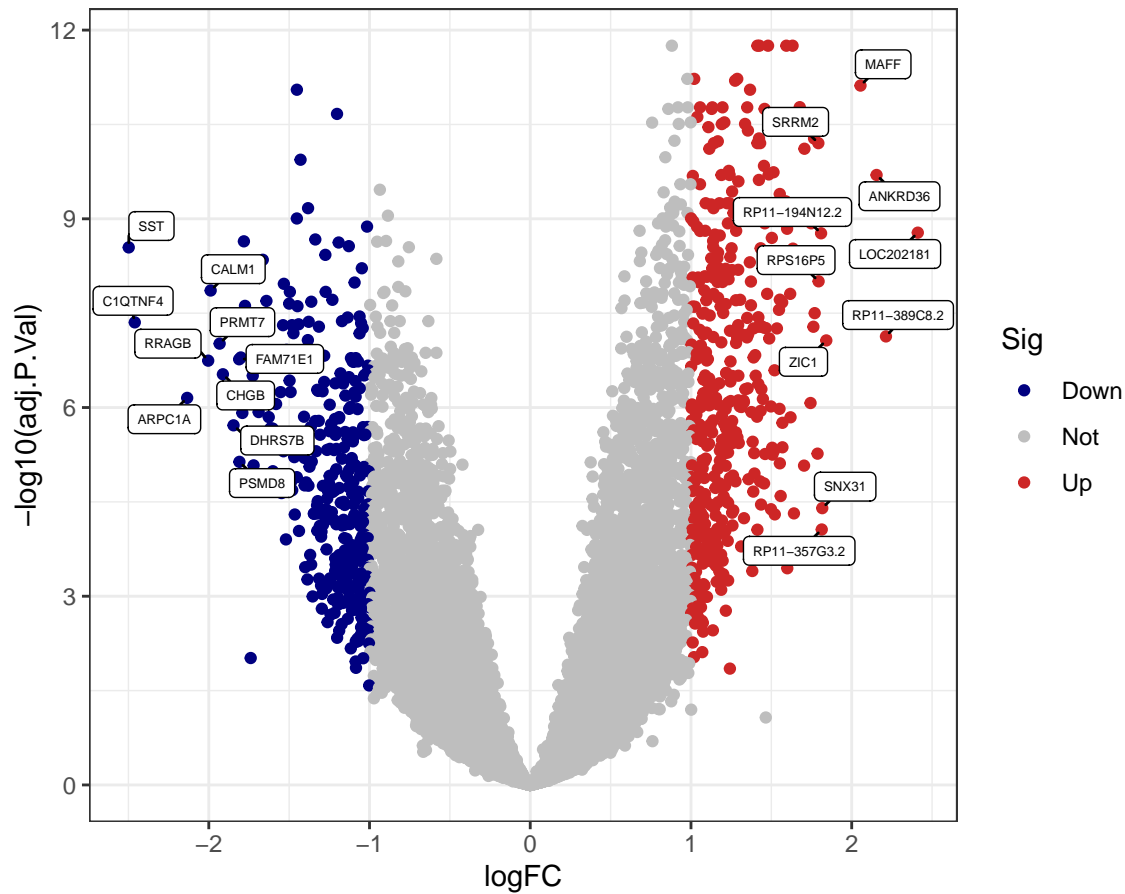

Supplement: Supplementary file 1 [file DataSheet1.zip › 1520845Supplementary files/05火山图标注基因名称/vol.pdf]

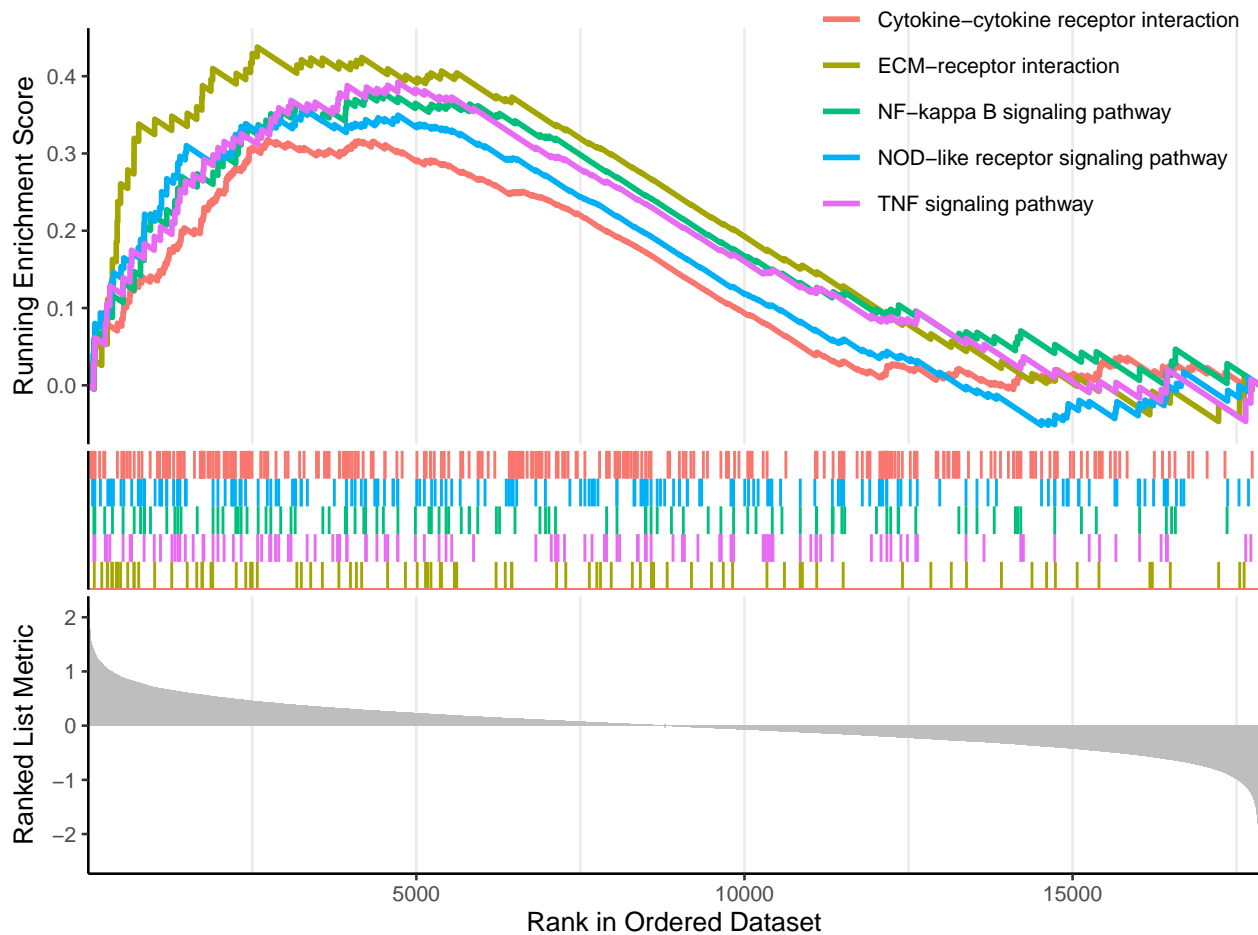

Supplement: Supplementary file 1 [file DataSheet1.zip › 1520845Supplementary files/06GSEA分析/上调.pdf]

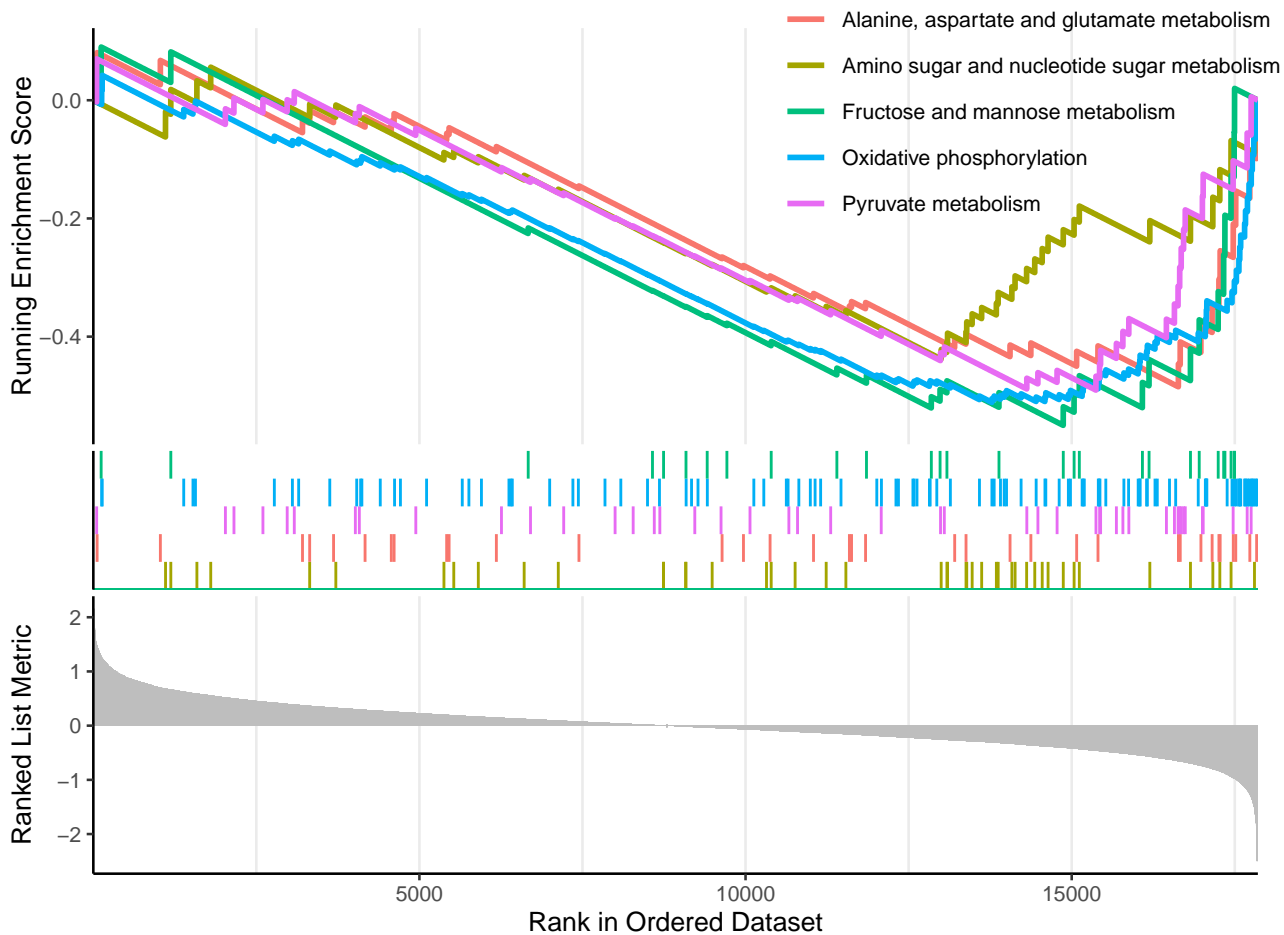

Supplement: Supplementary file 1 [file DataSheet1.zip › 1520845Supplementary files/06GSEA分析/下调.pdf]

## Biological Process

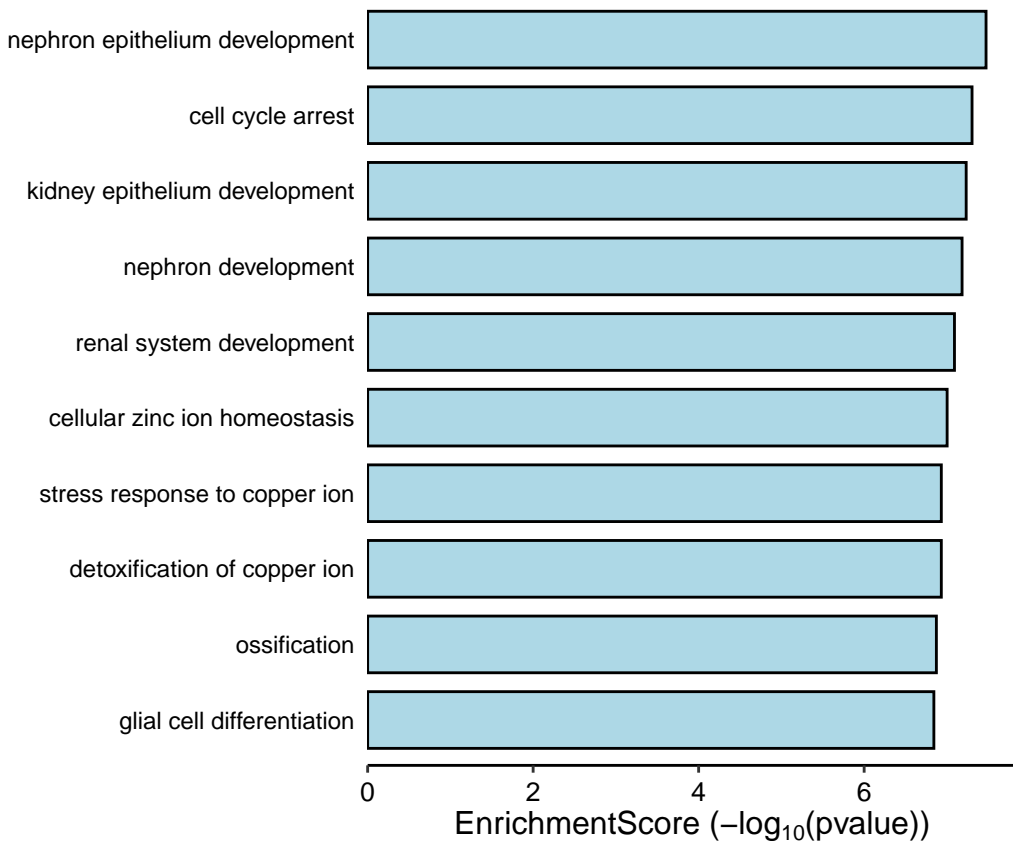

Supplement: Supplementary file 1 [file DataSheet1.zip › 1520845Supplementary files/07差异基因的GO富集分析/go.d3f24d25e5508754/BP_Enrichment_Score_barplot.pdf]

## Biological Process

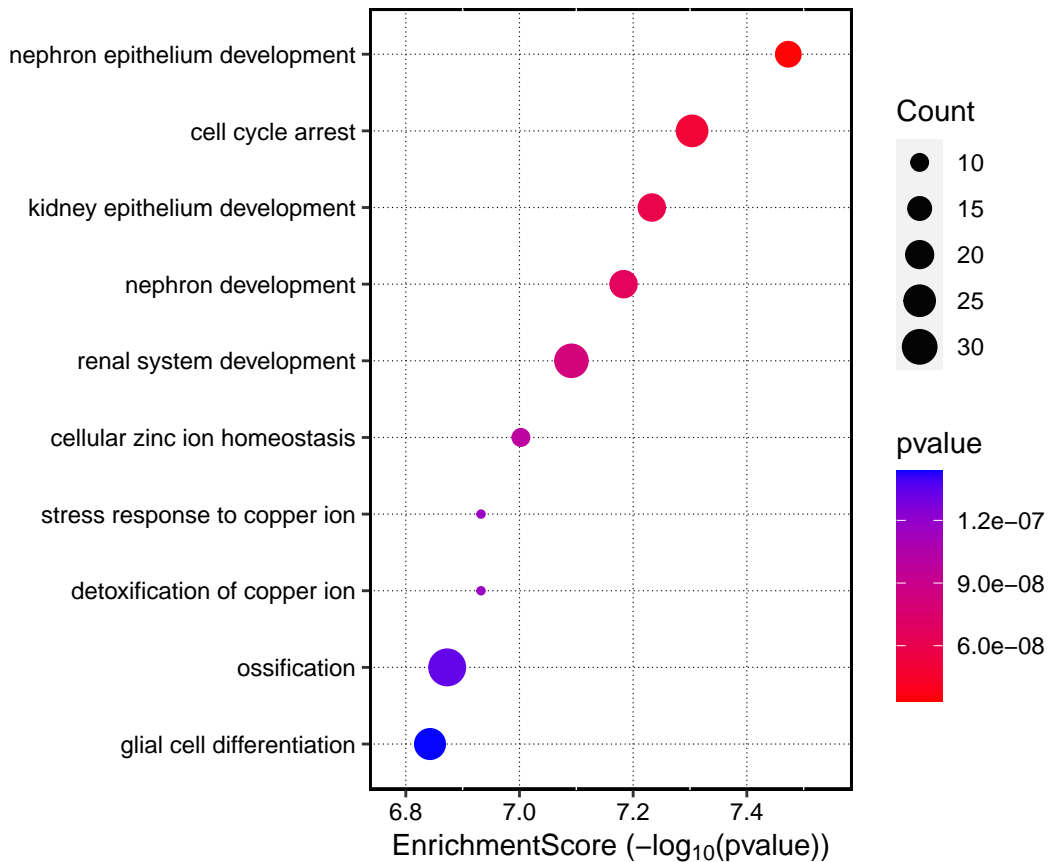

Supplement: Supplementary file 1 [file DataSheet1.zip › 1520845Supplementary files/07差异基因的GO富集分析/go.d3f24d25e5508754/BP_Enrichment_Score_dotplot.pdf]

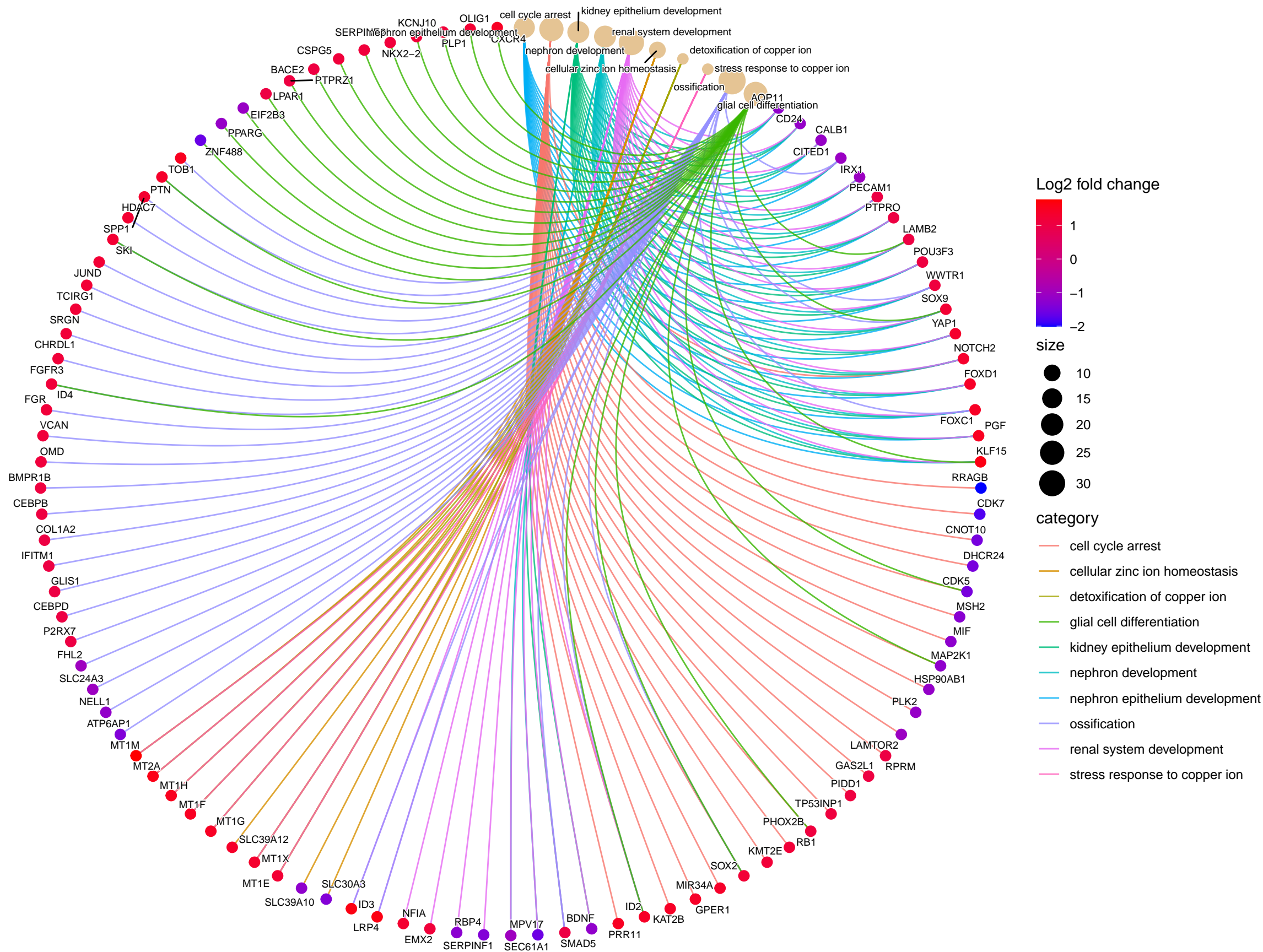

Supplement: Supplementary file 1 [file DataSheet1.zip › 1520845Supplementary files/07差异基因的GO富集分析/go.d3f24d25e5508754/BP_cnetplot.pdf]

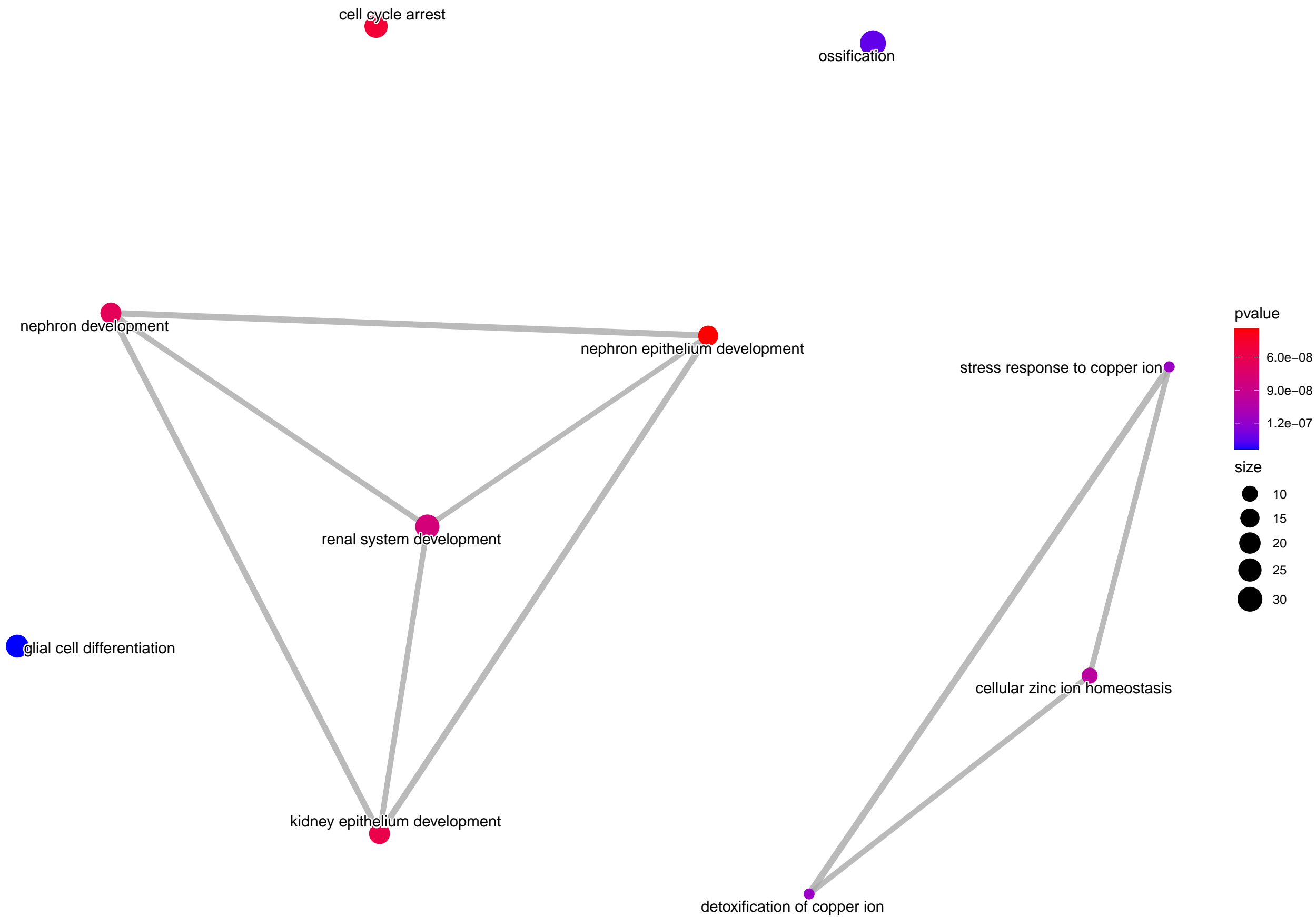

Supplement: Supplementary file 1 [file DataSheet1.zip › 1520845Supplementary files/07差异基因的GO富集分析/go.d3f24d25e5508754/BP_emapplot.pdf]

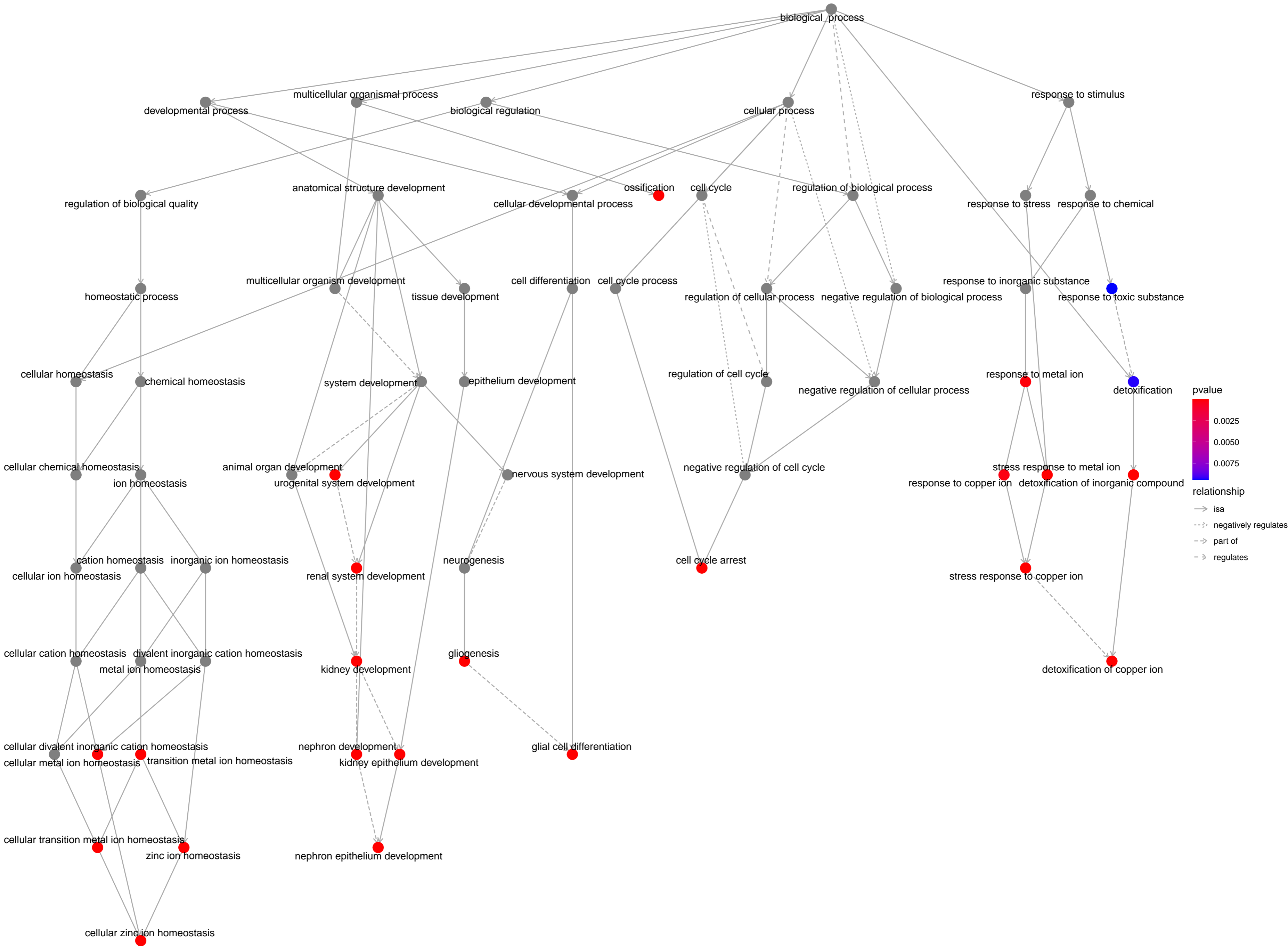

Supplement: Supplementary file 1 [file DataSheet1.zip › 1520845Supplementary files/07差异基因的GO富集分析/go.d3f24d25e5508754/BP_goplot.pdf]

# Cellular Component

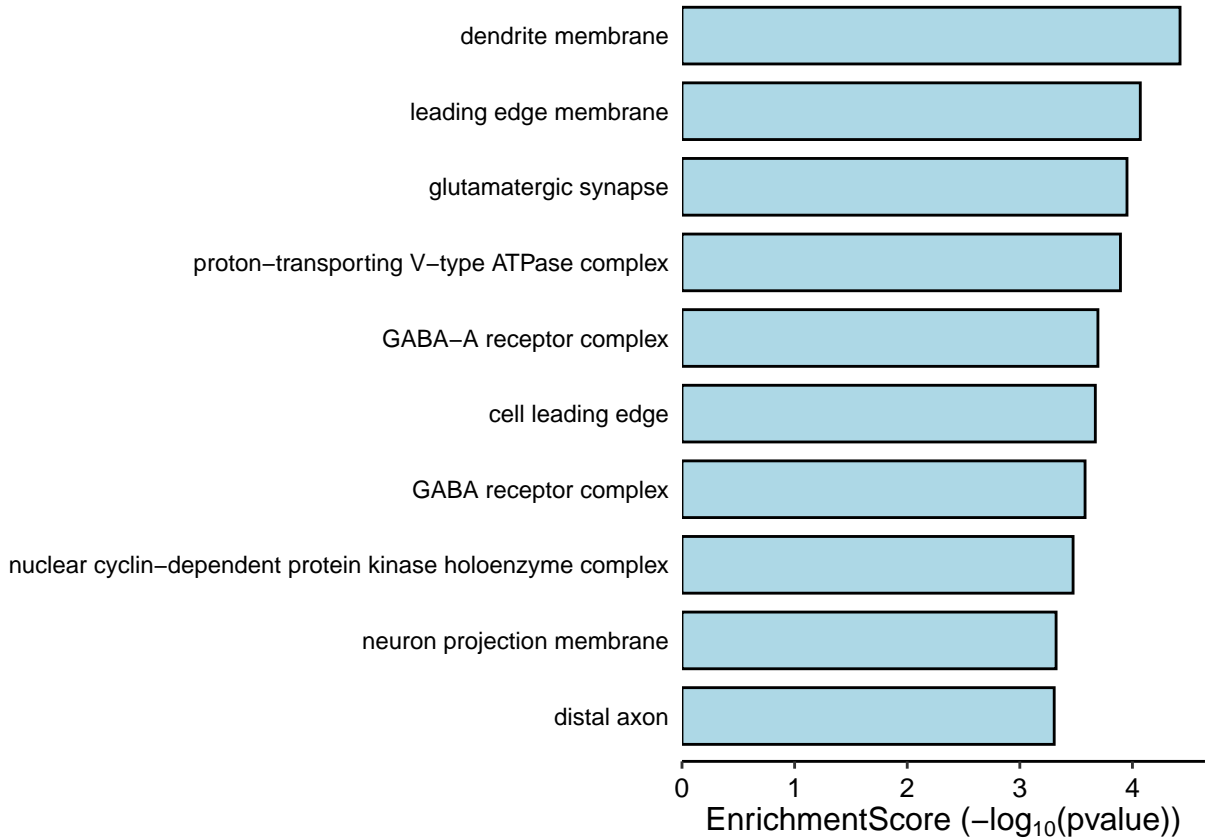

Supplement: Supplementary file 1 [file DataSheet1.zip › 1520845Supplementary files/07差异基因的GO富集分析/go.d3f24d25e5508754/CC_Enrichment_Score_barplot.pdf]

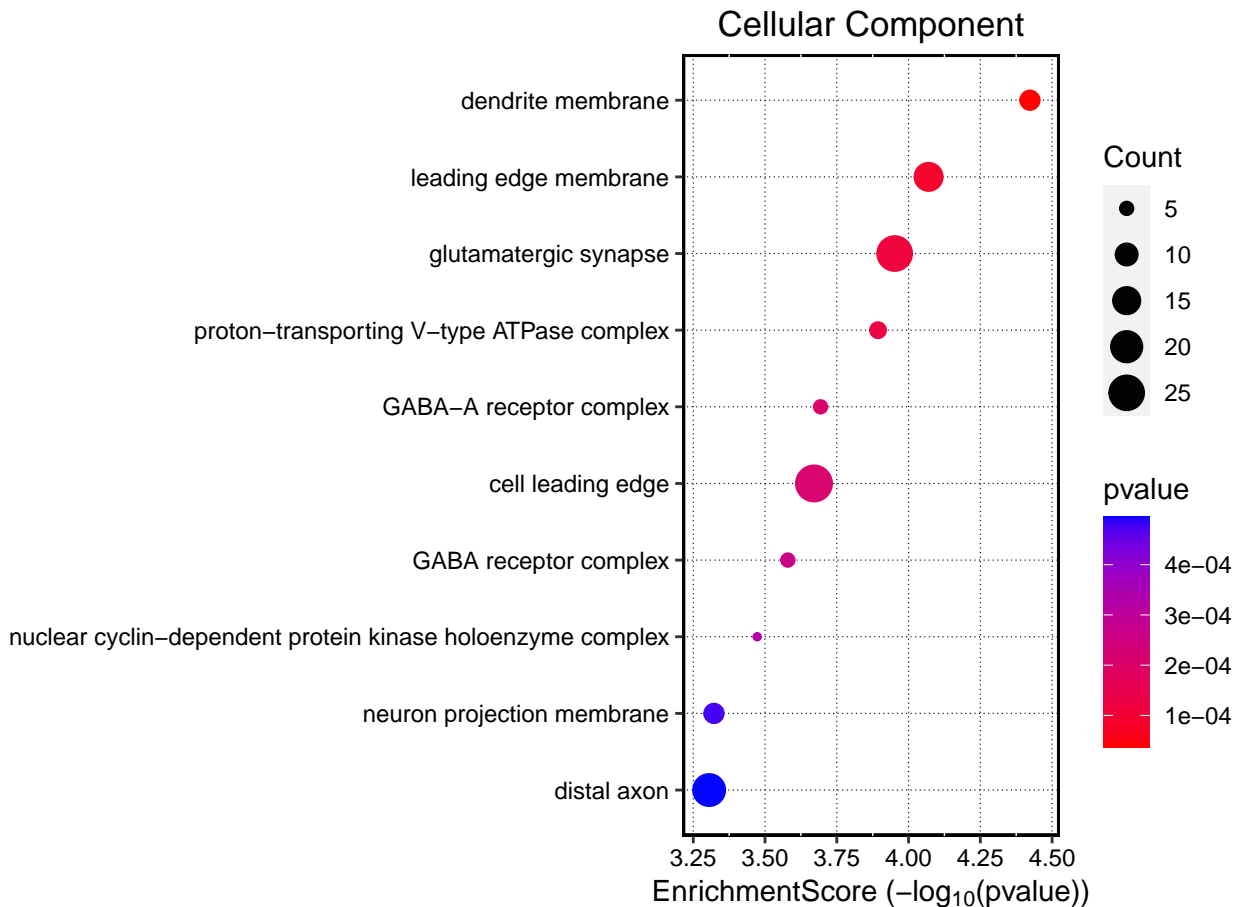

Supplement: Supplementary file 1 [file DataSheet1.zip › 1520845Supplementary files/07差异基因的GO富集分析/go.d3f24d25e5508754/CC_Enrichment_Score_dotplot.pdf]

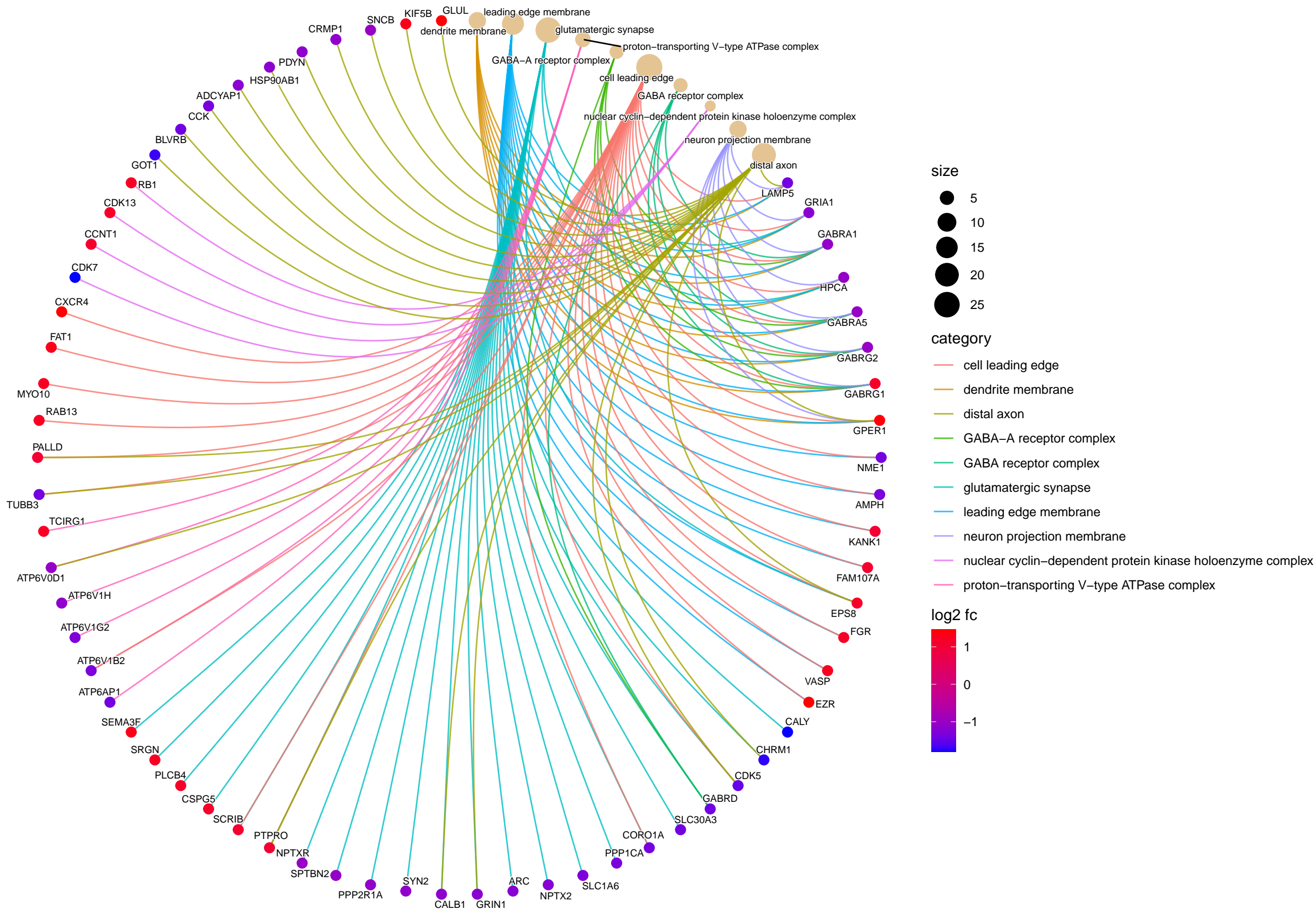

Supplement: Supplementary file 1 [file DataSheet1.zip › 1520845Supplementary files/07差异基因的GO富集分析/go.d3f24d25e5508754/CC_cnetplot.pdf]

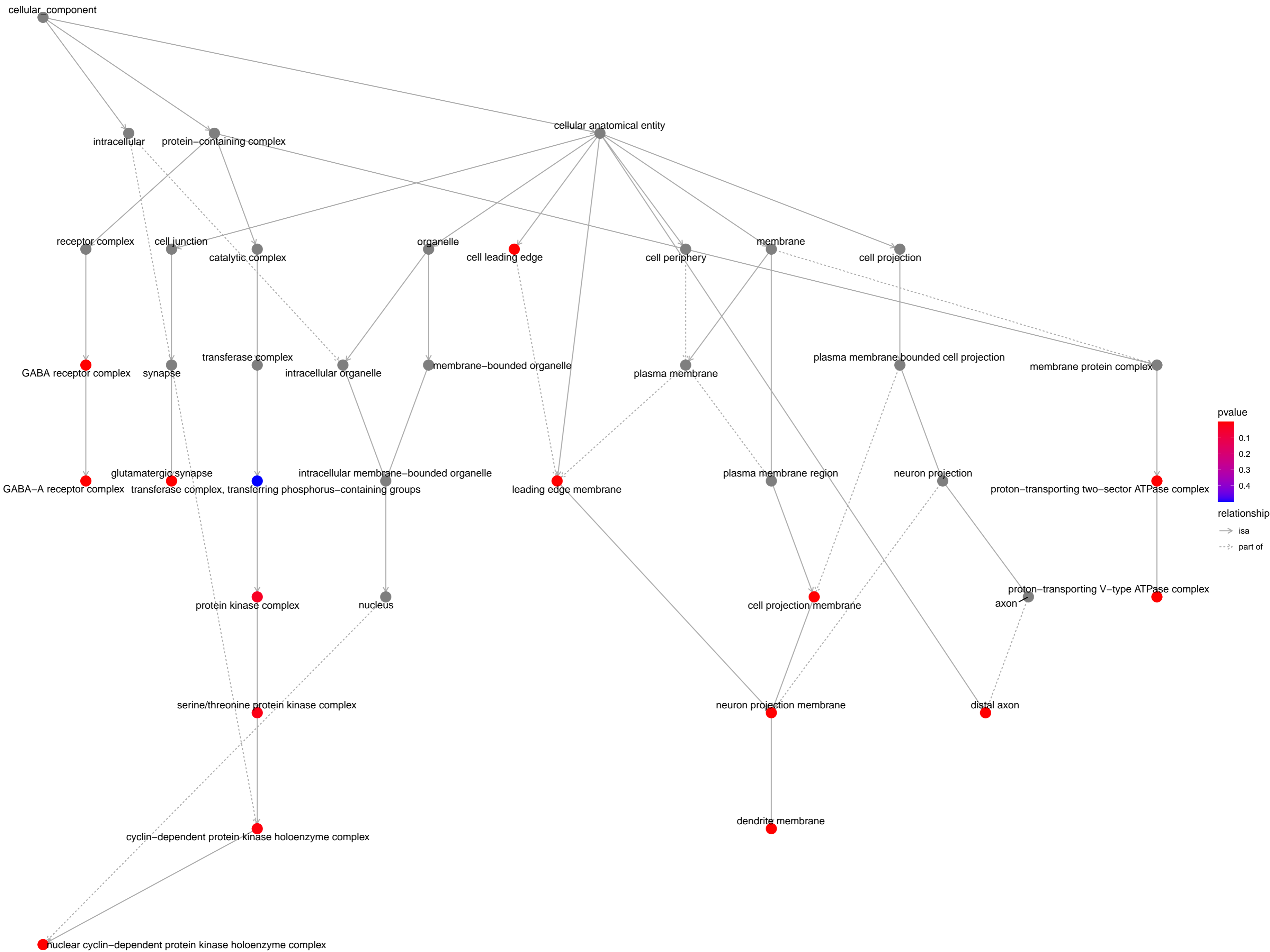

Supplement: Supplementary file 1 [file DataSheet1.zip › 1520845Supplementary files/07差异基因的GO富集分析/go.d3f24d25e5508754/CC_goplot.pdf]

# GO Results of Three Ontologies

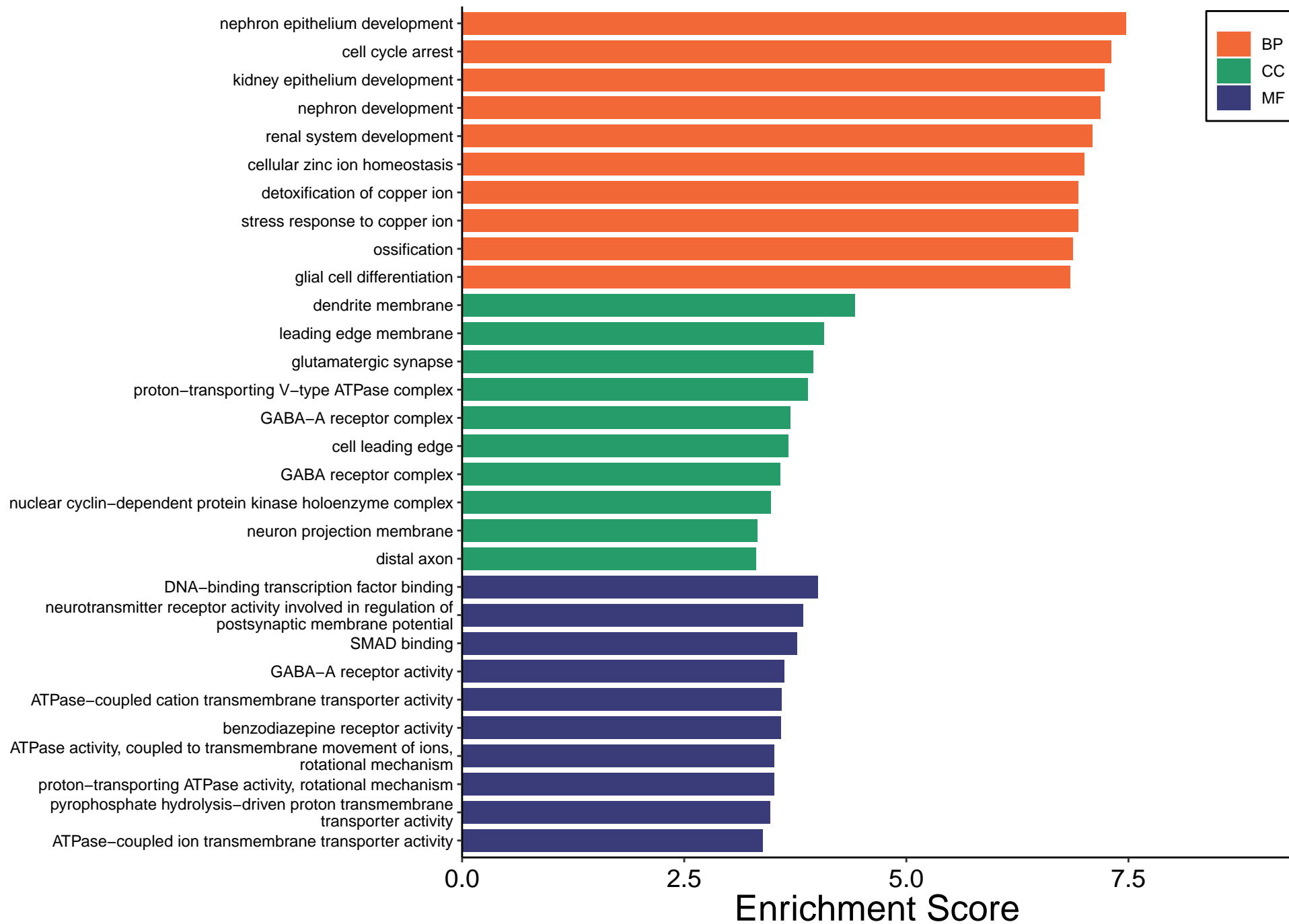

Supplement: Supplementary file 1 [file DataSheet1.zip › 1520845Supplementary files/07差异基因的GO富集分析/go.d3f24d25e5508754/GO_Three_Ontologies.pdf]

## Molecular Function

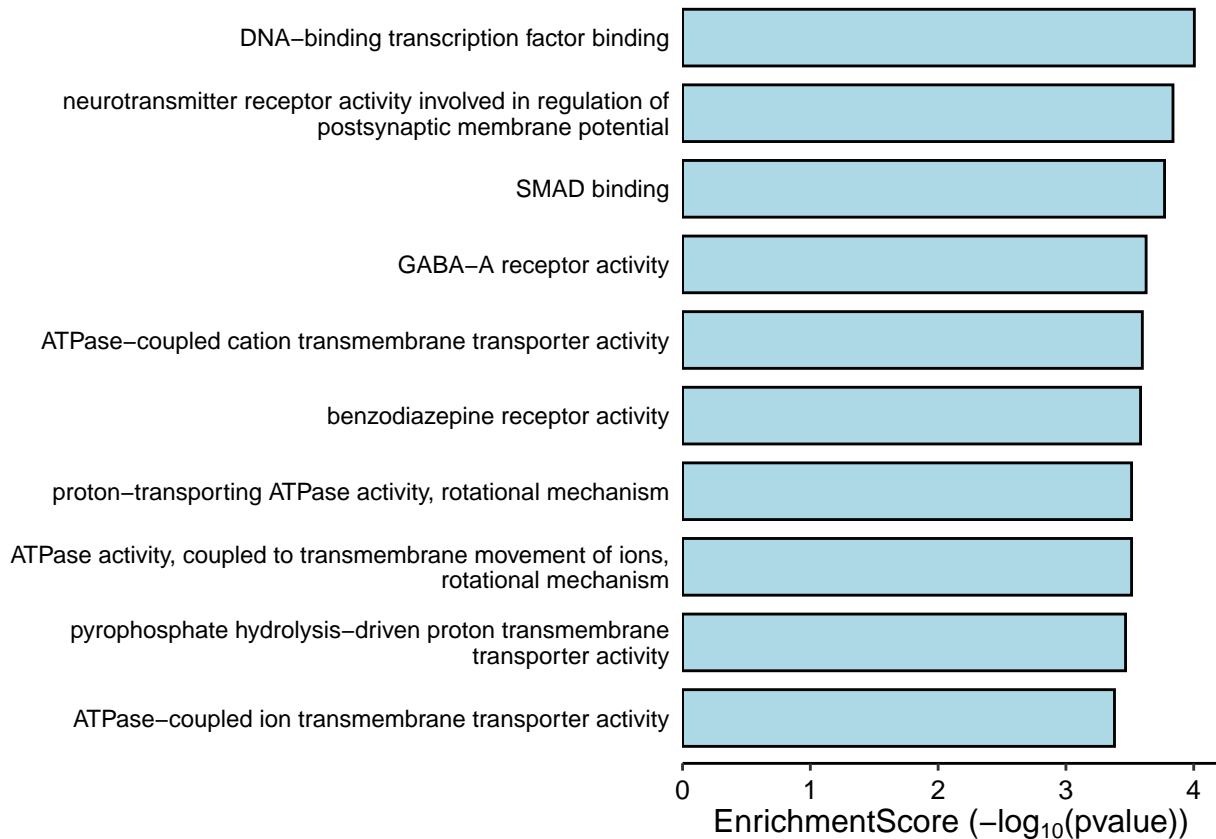

Supplement: Supplementary file 1 [file DataSheet1.zip › 1520845Supplementary files/07差异基因的GO富集分析/go.d3f24d25e5508754/MF_Enrichment_Score_barplot.pdf]

# Molecular Function

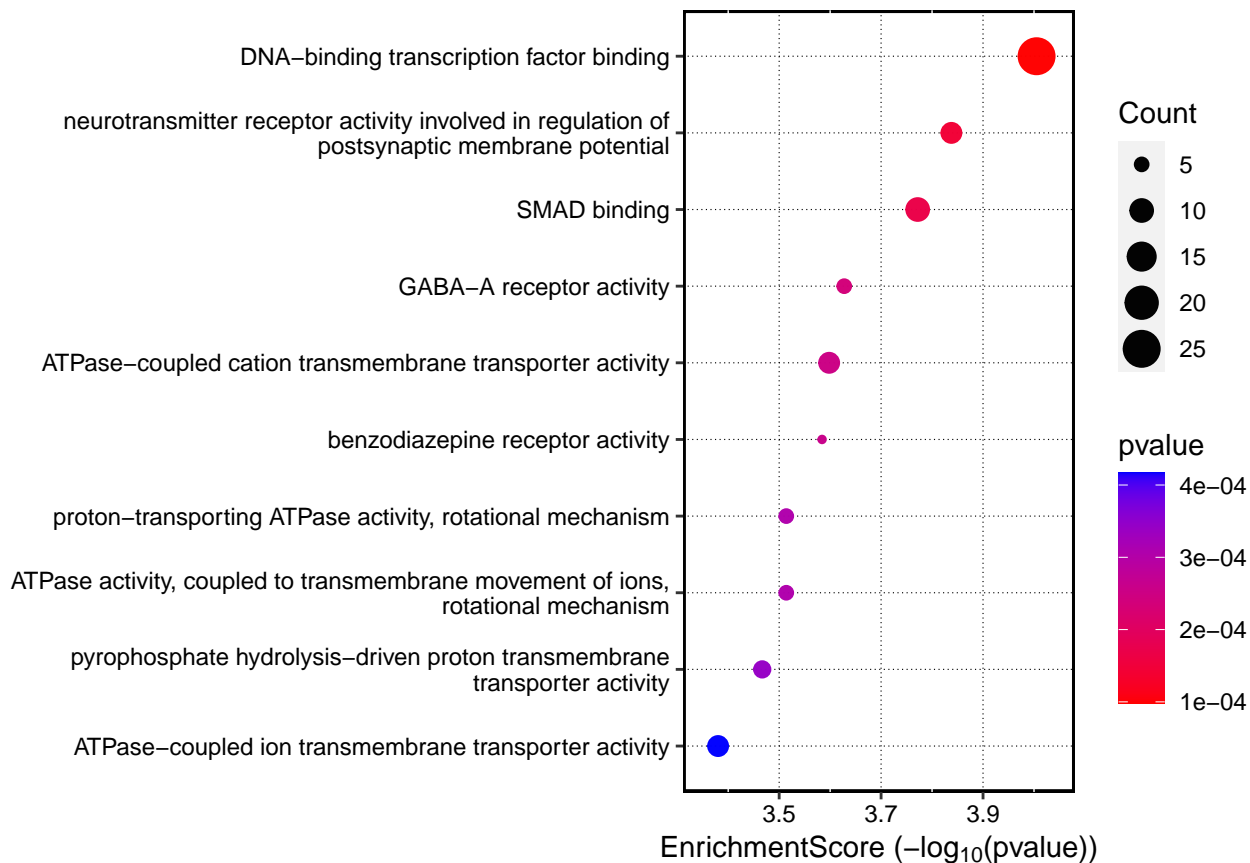

Supplement: Supplementary file 1 [file DataSheet1.zip › 1520845Supplementary files/07差异基因的GO富集分析/go.d3f24d25e5508754/MF_Enrichment_Score_dotplot.pdf]

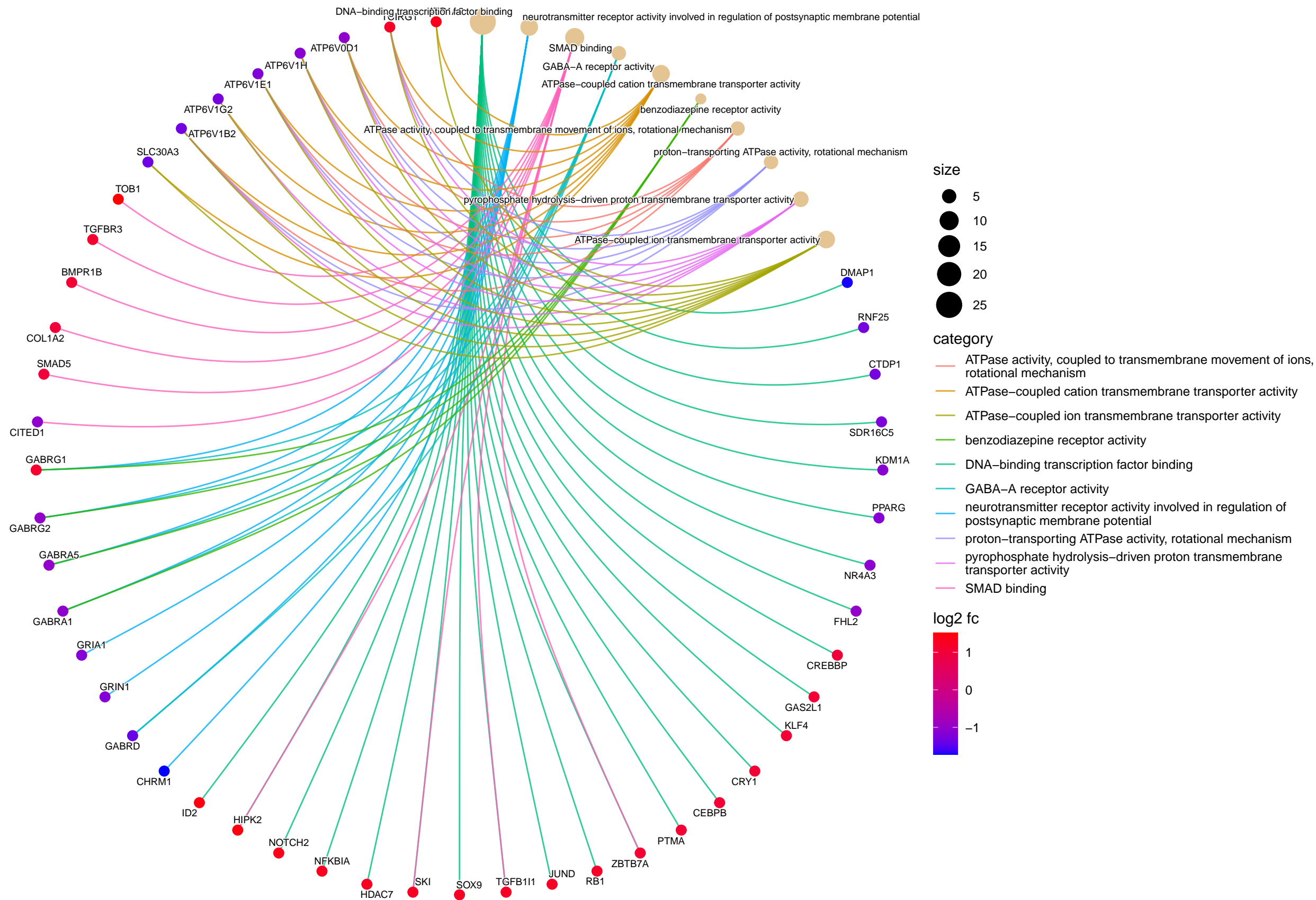

Supplement: Supplementary file 1 [file DataSheet1.zip › 1520845Supplementary files/07差异基因的GO富集分析/go.d3f24d25e5508754/MF_cnetplot.pdf]

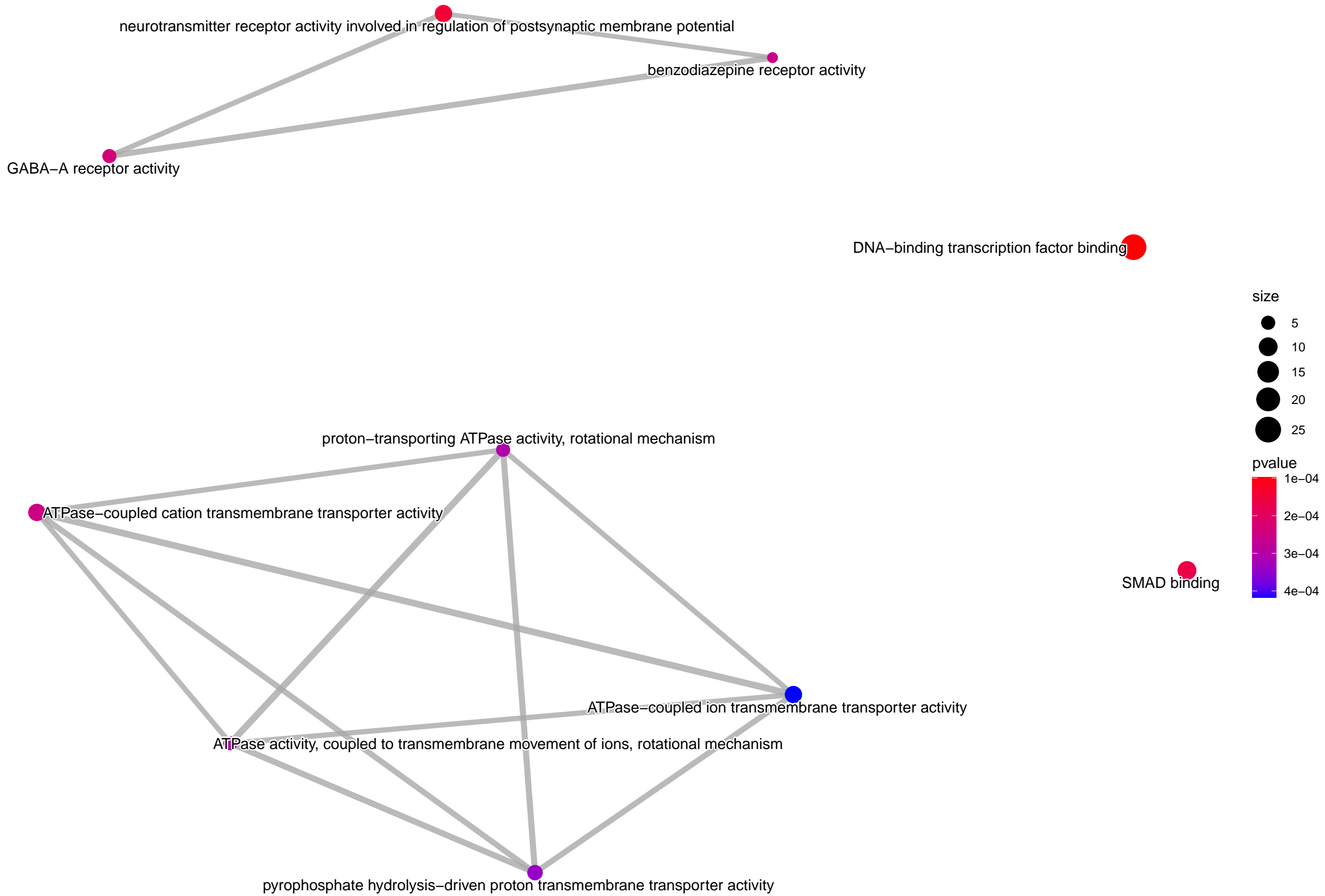

Supplement: Supplementary file 1 [file DataSheet1.zip › 1520845Supplementary files/07差异基因的GO富集分析/go.d3f24d25e5508754/MF_emapplot.pdf]

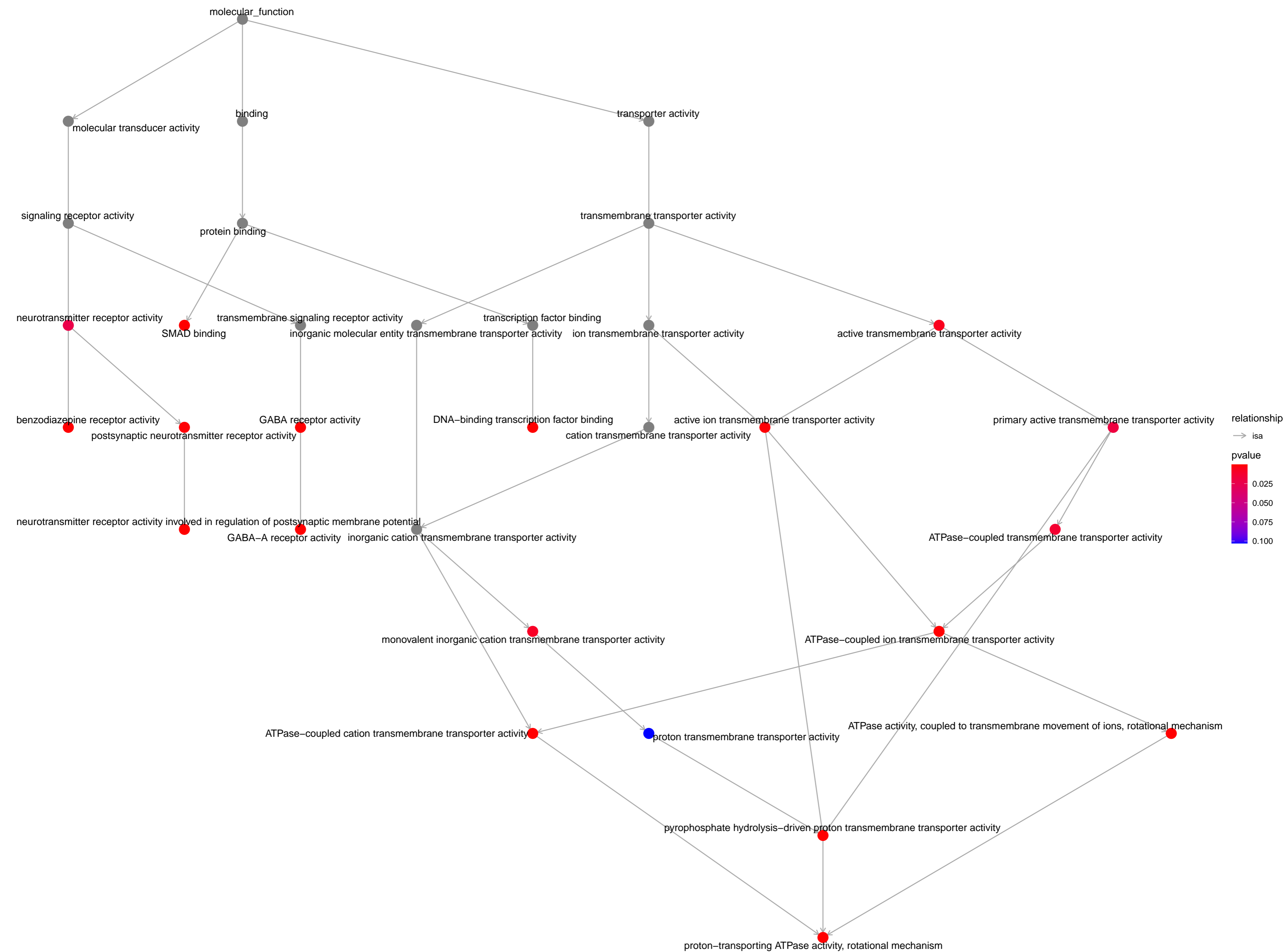

Supplement: Supplementary file 1 [file DataSheet1.zip › 1520845Supplementary files/07差异基因的GO富集分析/go.d3f24d25e5508754/MF_goplot.pdf]

# Pathway Analysis

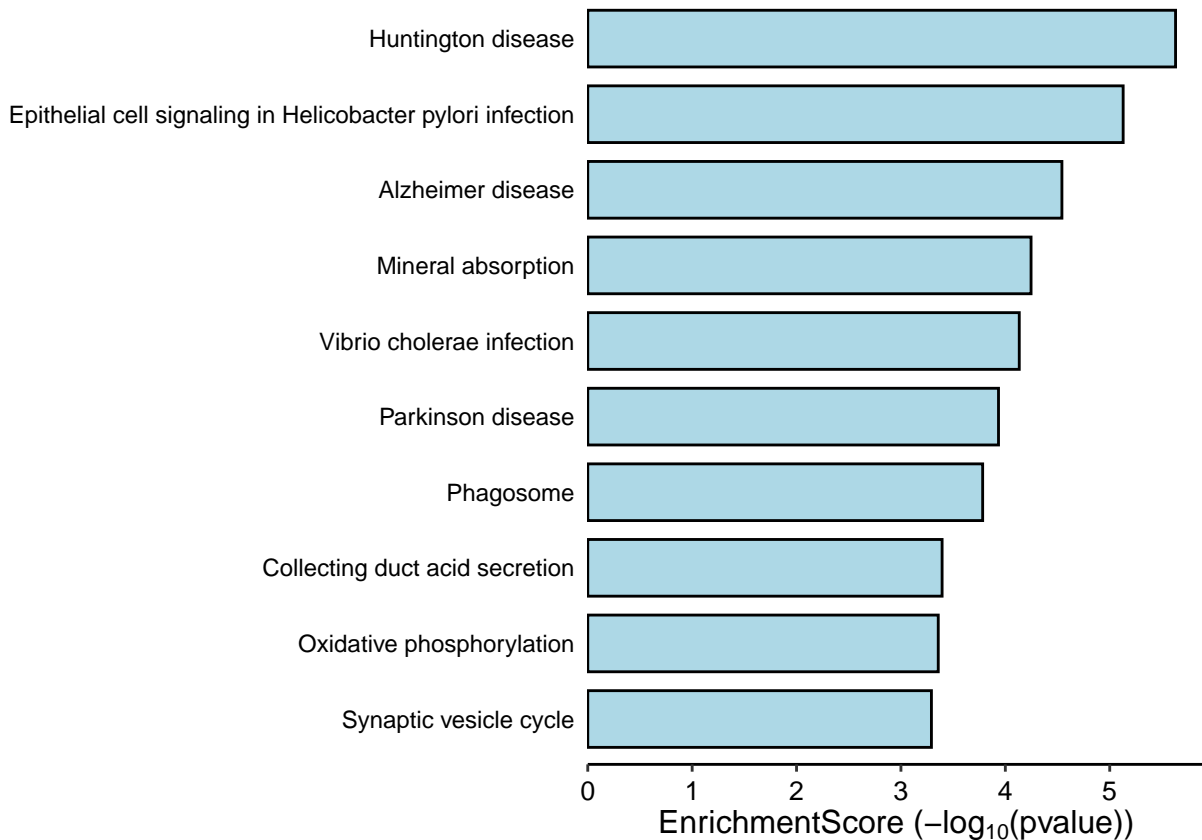

Supplement: Supplementary file 1 [file DataSheet1.zip › 1520845Supplementary files/08差异基因的KEGG富集分析/pathway.d3f24d25e5508754/Pathway_Enrichment_Score_barplot.pdf]

## Pathway Analysis

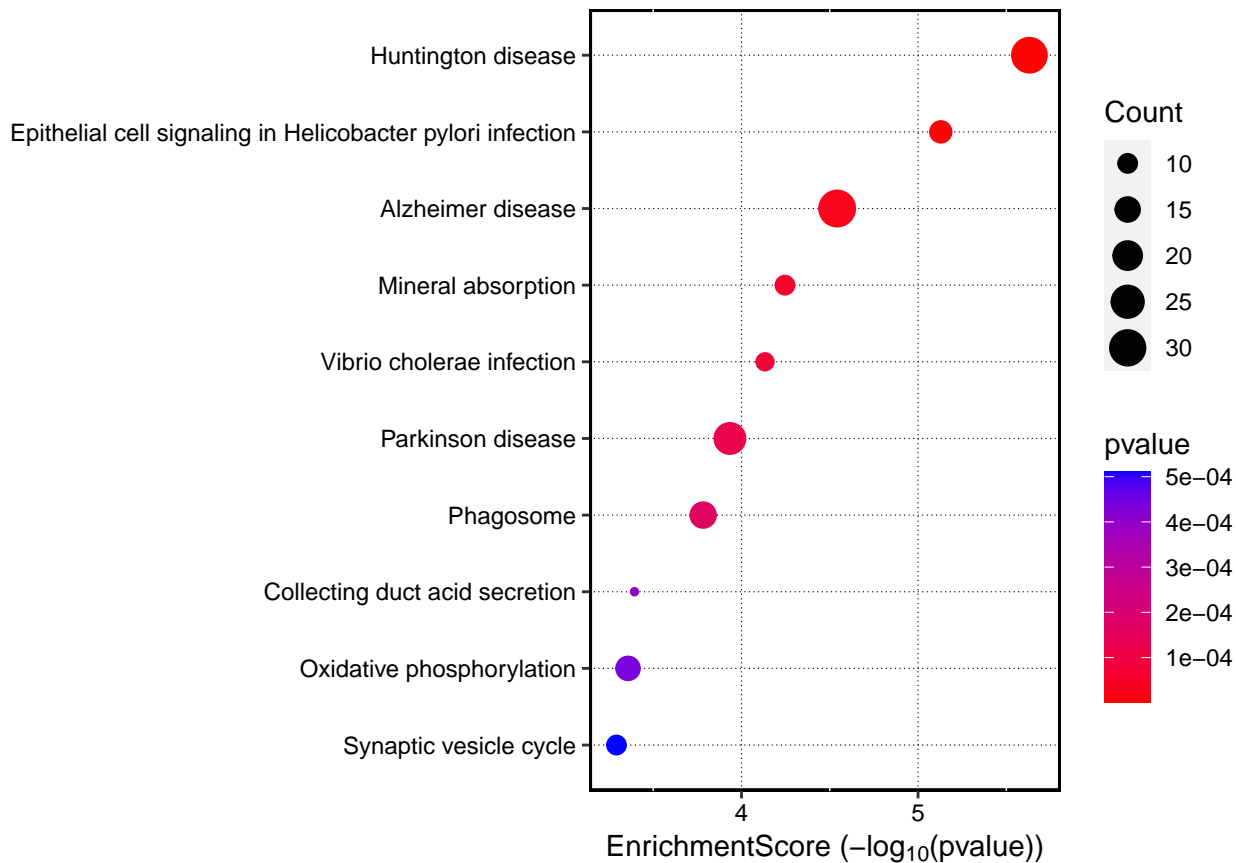

Supplement: Supplementary file 1 [file DataSheet1.zip › 1520845Supplementary files/08差异基因的KEGG富集分析/pathway.d3f24d25e5508754/Pathway_Enrichment_Score_dotplot.pdf]

● Mineral absorption

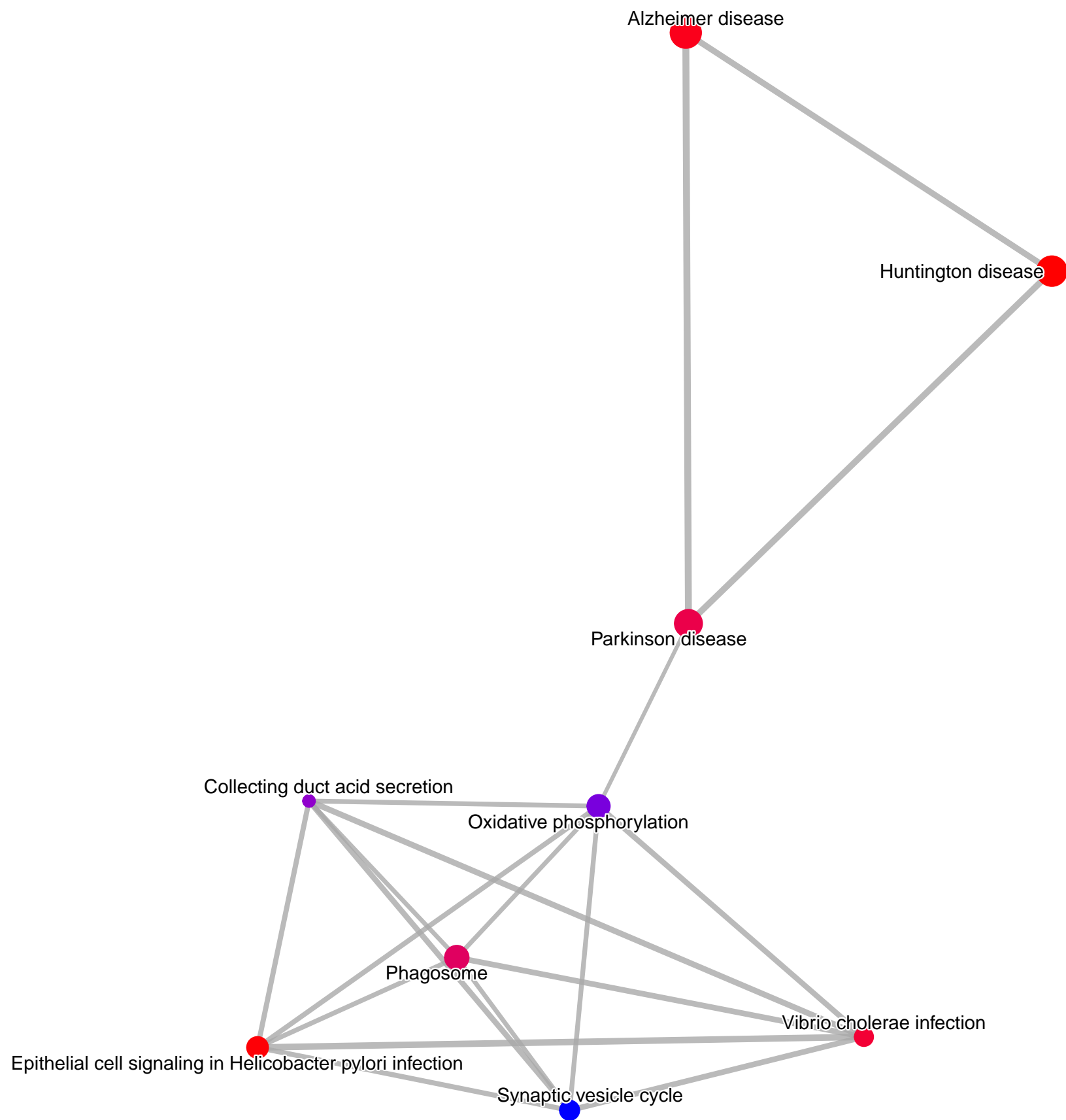

Supplement: Supplementary file 1 [file DataSheet1.zip › 1520845Supplementary files/08差异基因的KEGG富集分析/pathway.d3f24d25e5508754/Pathway_emapplot.pdf]

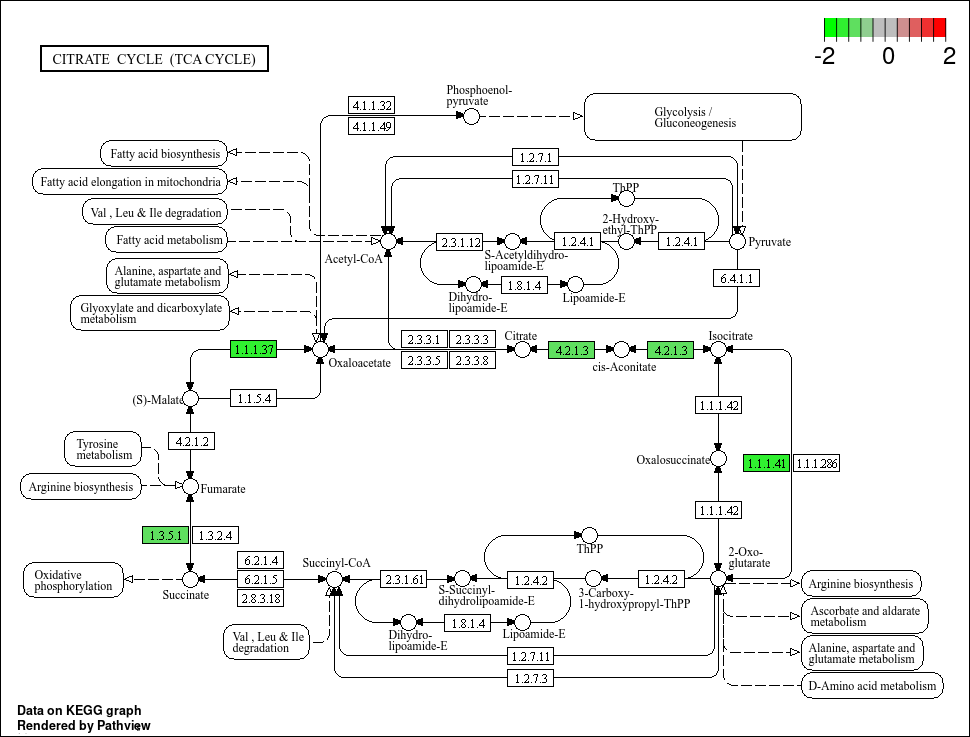

Supplement: Supplementary file 1 [file DataSheet1.zip › 1520845Supplementary files/08差异基因的KEGG富集分析/pathway.d3f24d25e5508754/hsa00020.pathview.png]

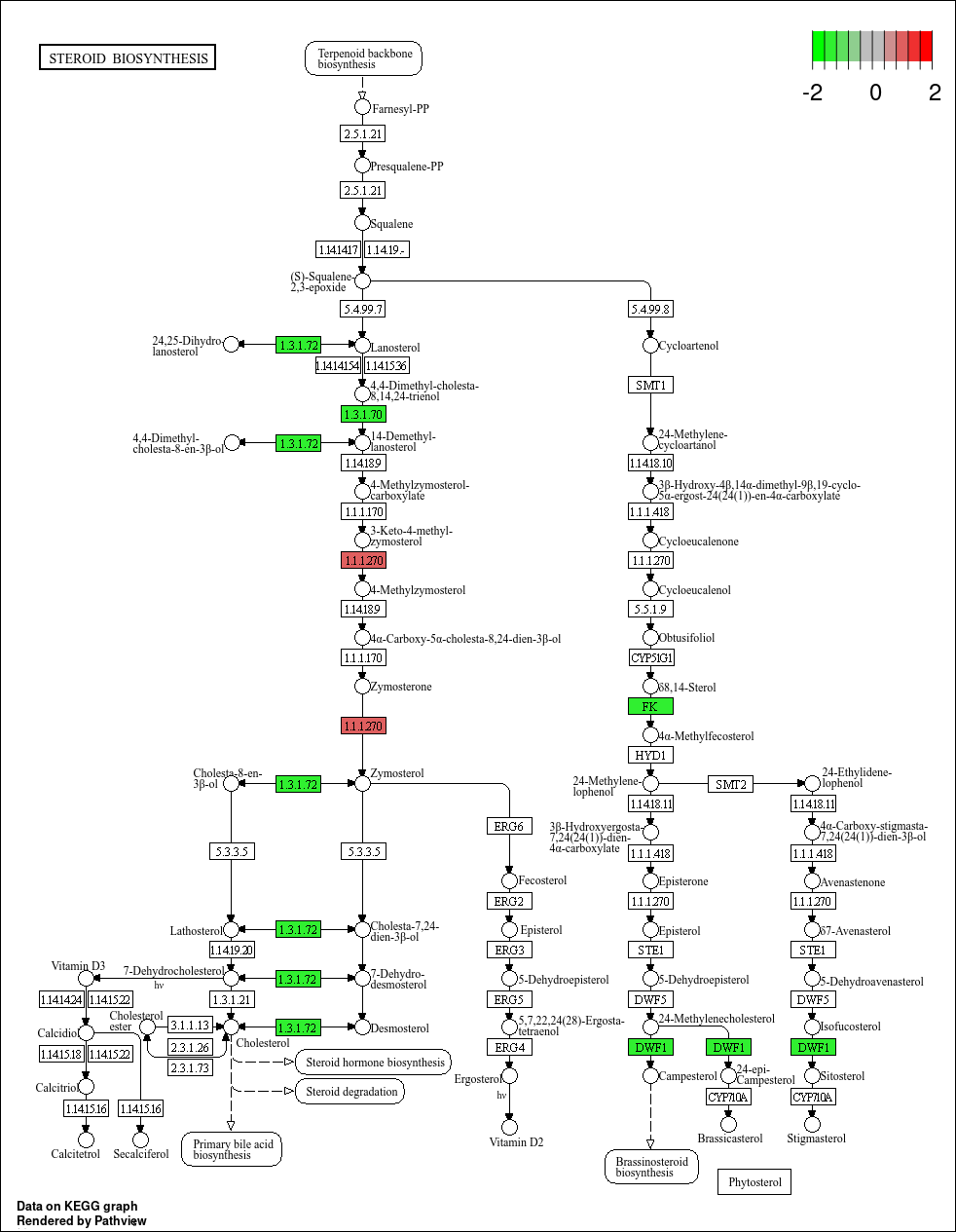

Supplement: Supplementary file 1 [file DataSheet1.zip › 1520845Supplementary files/08差异基因的KEGG富集分析/pathway.d3f24d25e5508754/hsa00100.pathview.png]

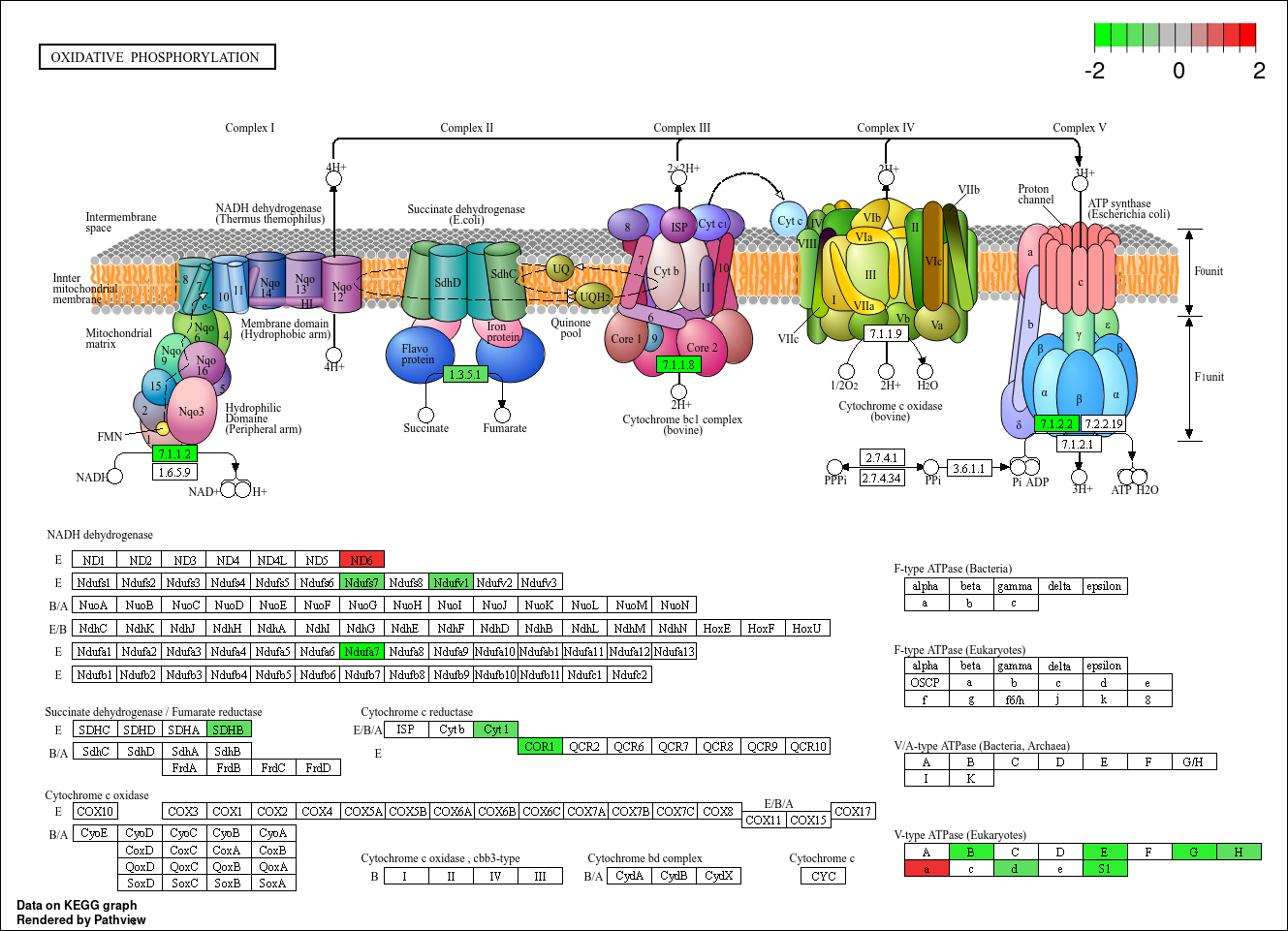

Supplement: Supplementary file 1 [file DataSheet1.zip › 1520845Supplementary files/08差异基因的KEGG富集分析/pathway.d3f24d25e5508754/hsa00190.pathview.png]

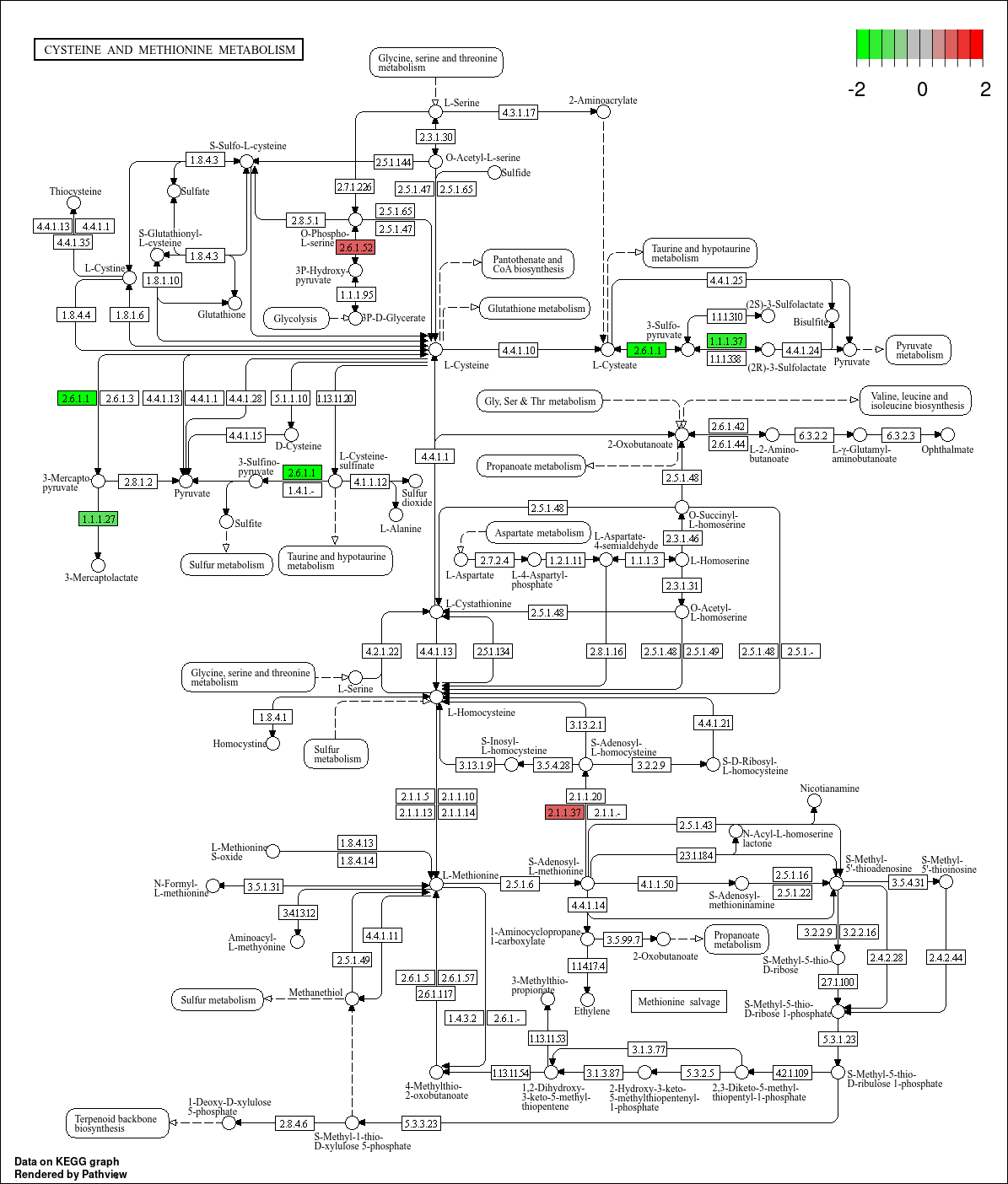

Supplement: Supplementary file 1 [file DataSheet1.zip › 1520845Supplementary files/08差异基因的KEGG富集分析/pathway.d3f24d25e5508754/hsa00270.pathview.png]

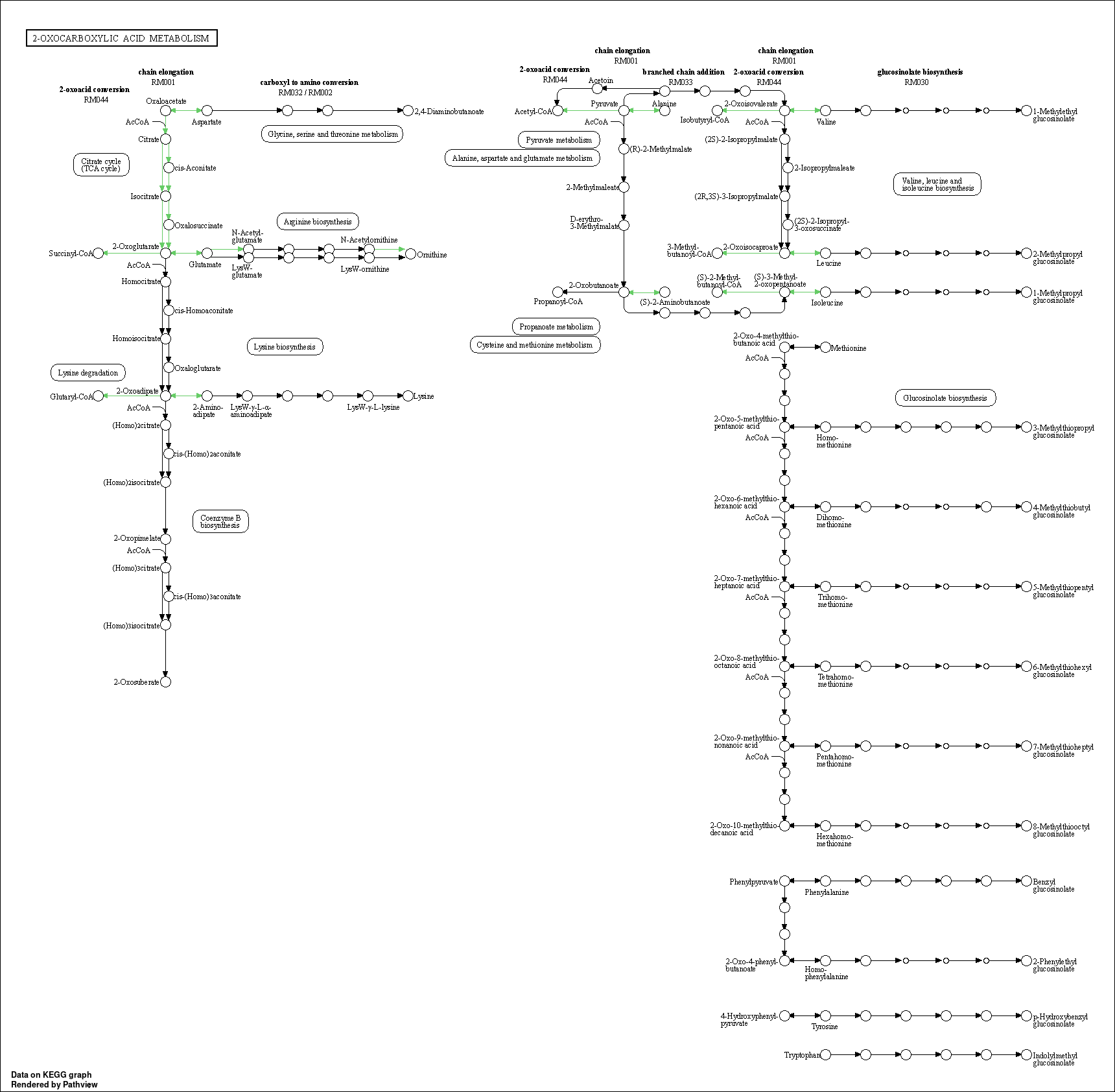

Supplement: Supplementary file 1 [file DataSheet1.zip › 1520845Supplementary files/08差异基因的KEGG富集分析/pathway.d3f24d25e5508754/hsa01210.pathview.png]

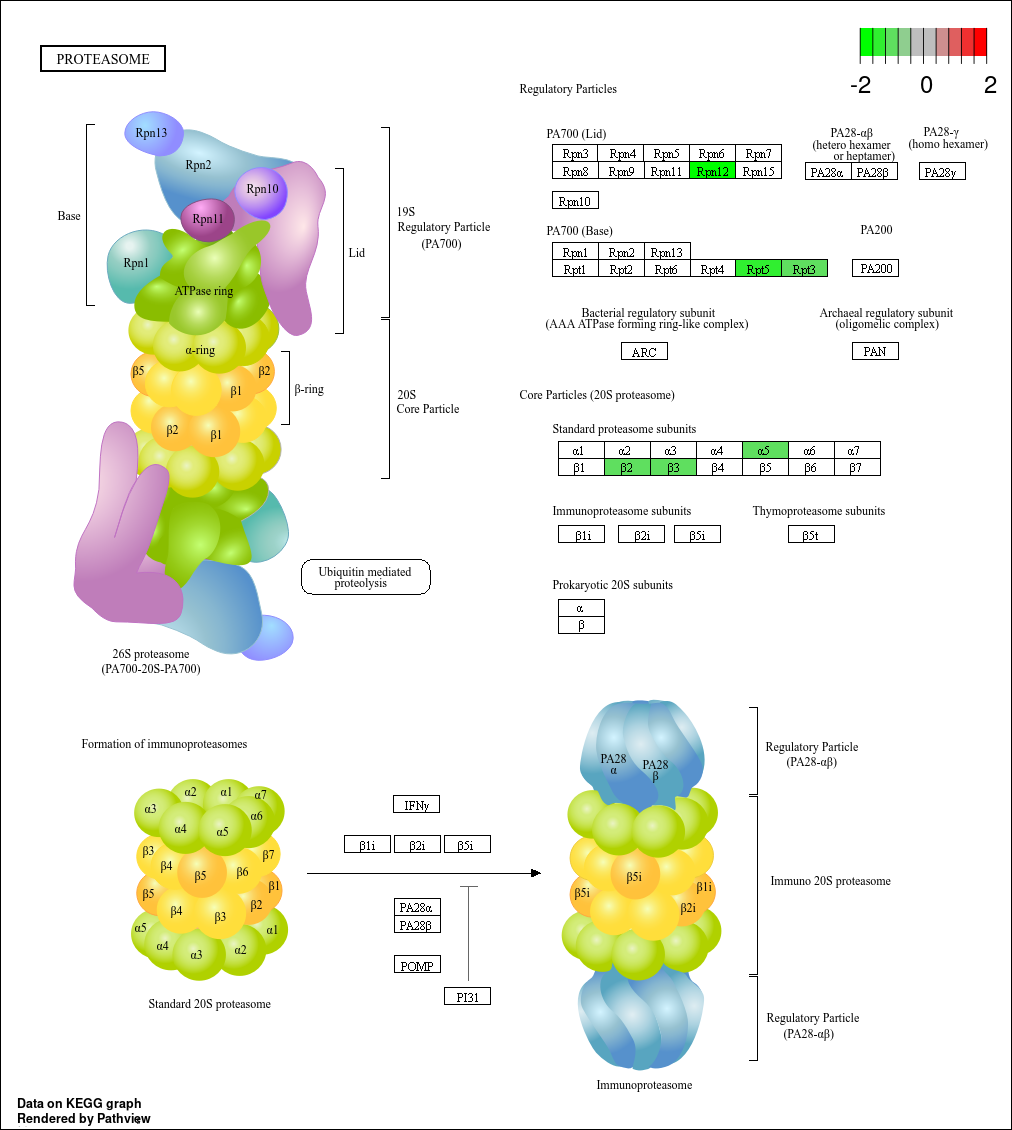

Supplement: Supplementary file 1 [file DataSheet1.zip › 1520845Supplementary files/08差异基因的KEGG富集分析/pathway.d3f24d25e5508754/hsa03050.pathview.png]

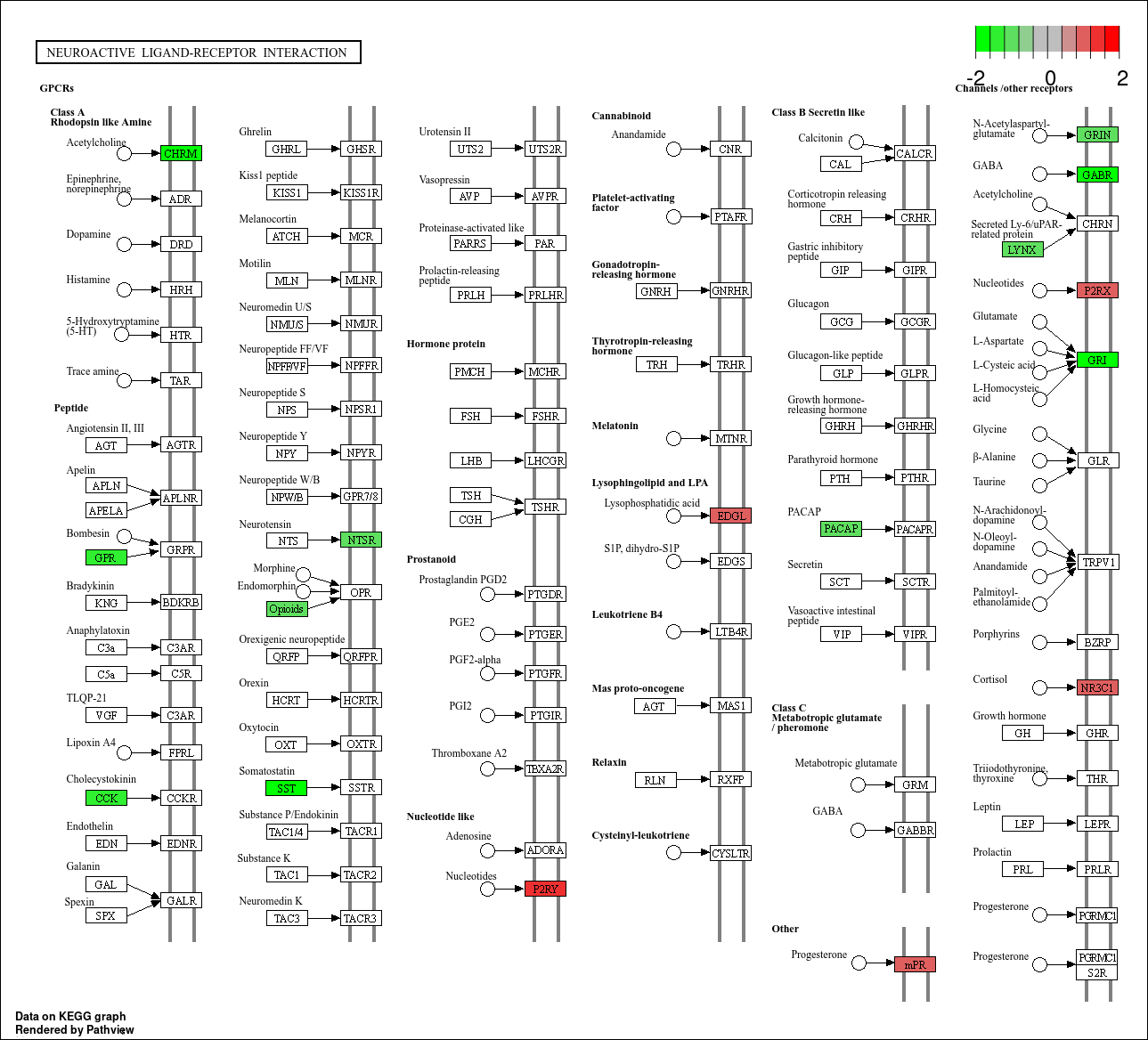

Supplement: Supplementary file 1 [file DataSheet1.zip › 1520845Supplementary files/08差异基因的KEGG富集分析/pathway.d3f24d25e5508754/hsa04080.pathview.png]

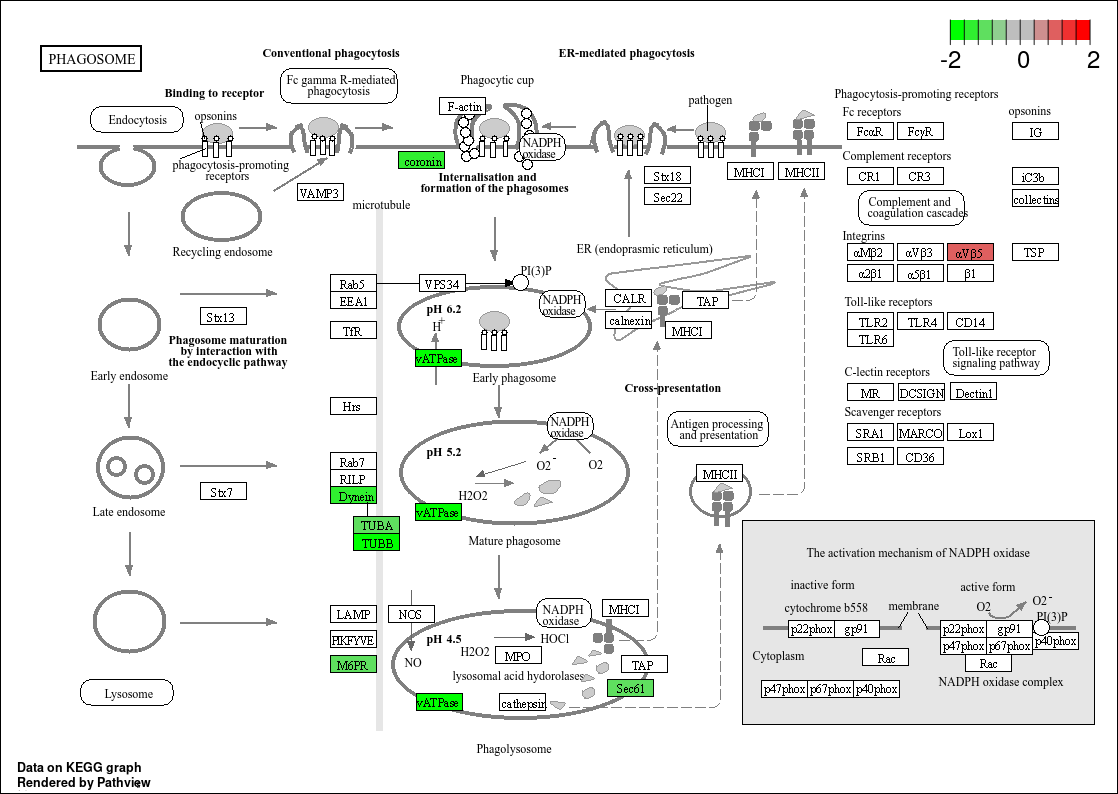

Supplement: Supplementary file 1 [file DataSheet1.zip › 1520845Supplementary files/08差异基因的KEGG富集分析/pathway.d3f24d25e5508754/hsa04145.pathview.png]

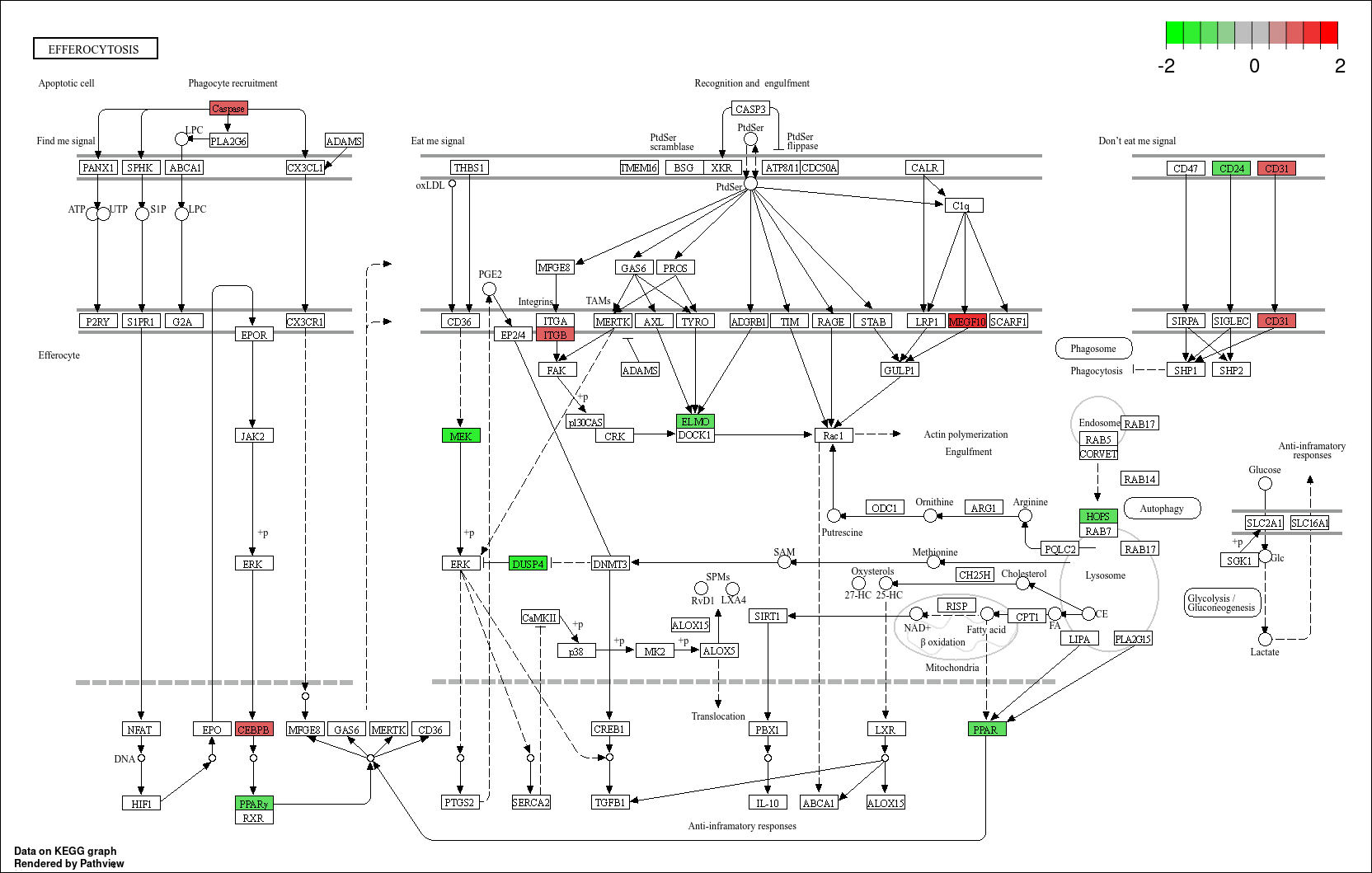

Supplement: Supplementary file 1 [file DataSheet1.zip › 1520845Supplementary files/08差异基因的KEGG富集分析/pathway.d3f24d25e5508754/hsa04148.pathview.png]

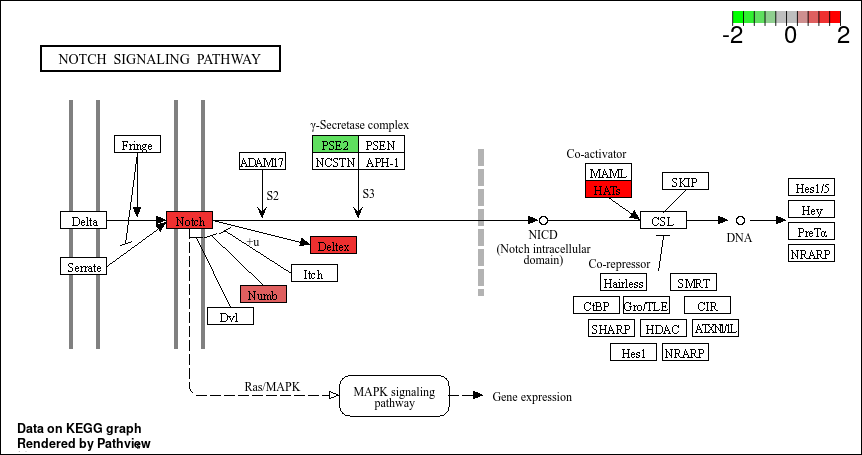

Supplement: Supplementary file 1 [file DataSheet1.zip › 1520845Supplementary files/08差异基因的KEGG富集分析/pathway.d3f24d25e5508754/hsa04330.pathview.png]

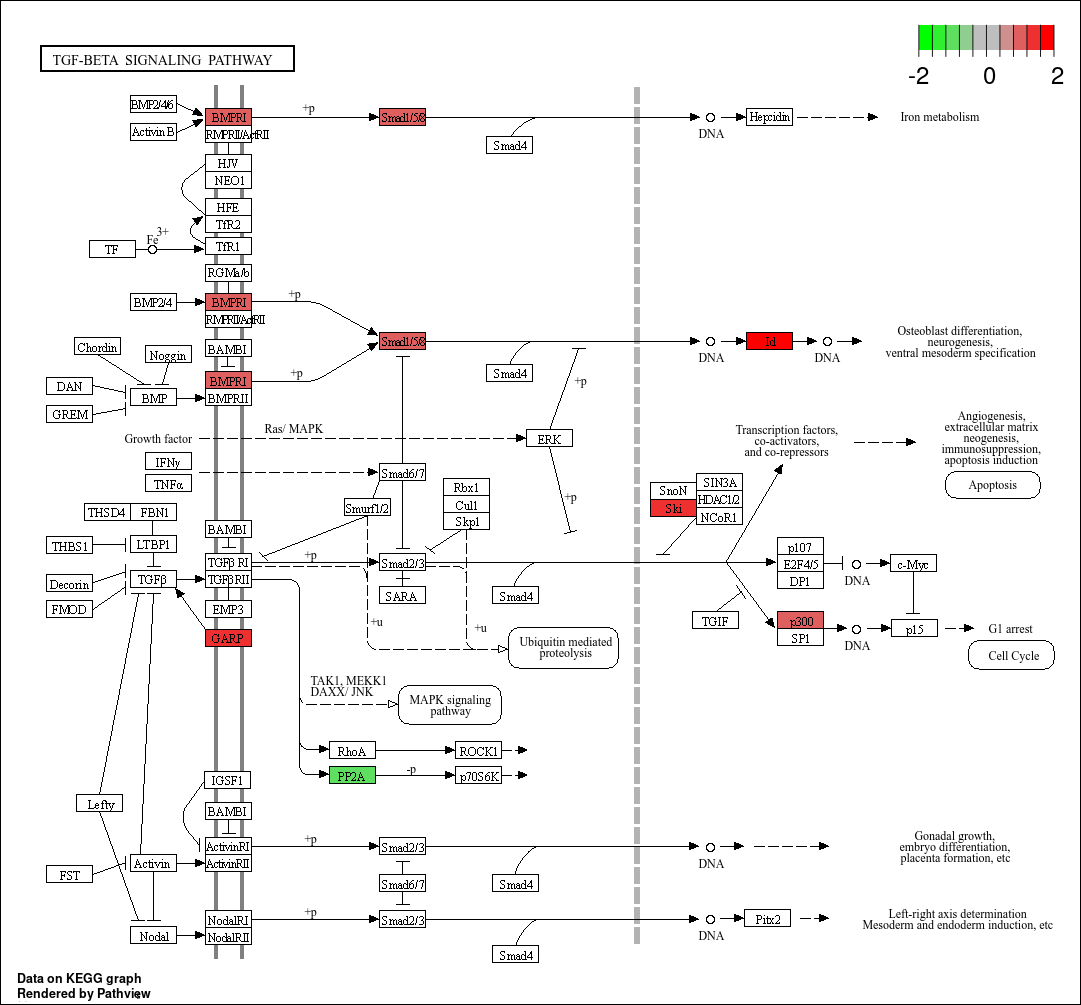

Supplement: Supplementary file 1 [file DataSheet1.zip › 1520845Supplementary files/08差异基因的KEGG富集分析/pathway.d3f24d25e5508754/hsa04350.pathview.png]

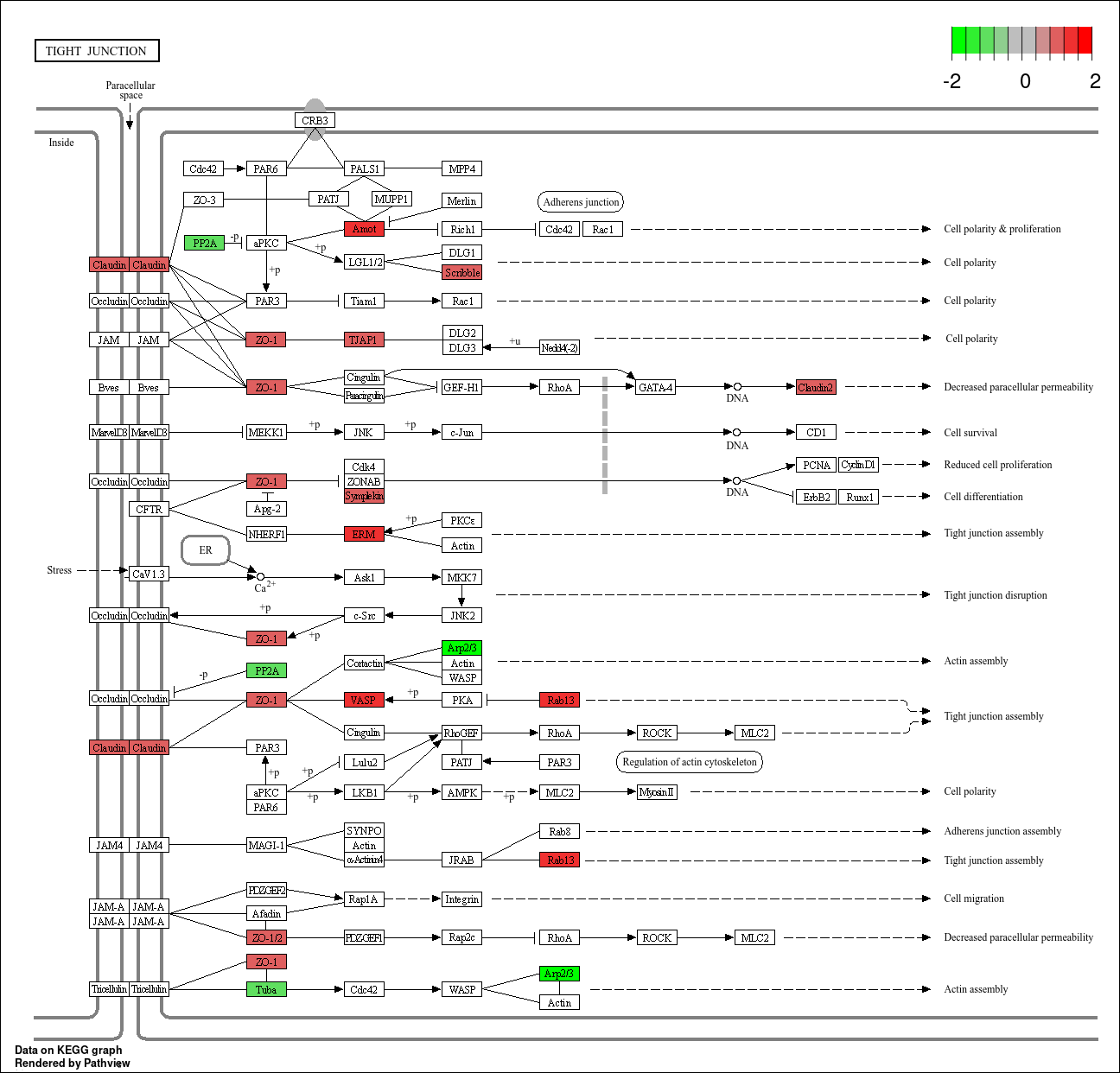

Supplement: Supplementary file 1 [file DataSheet1.zip › 1520845Supplementary files/08差异基因的KEGG富集分析/pathway.d3f24d25e5508754/hsa04530.pathview.png]

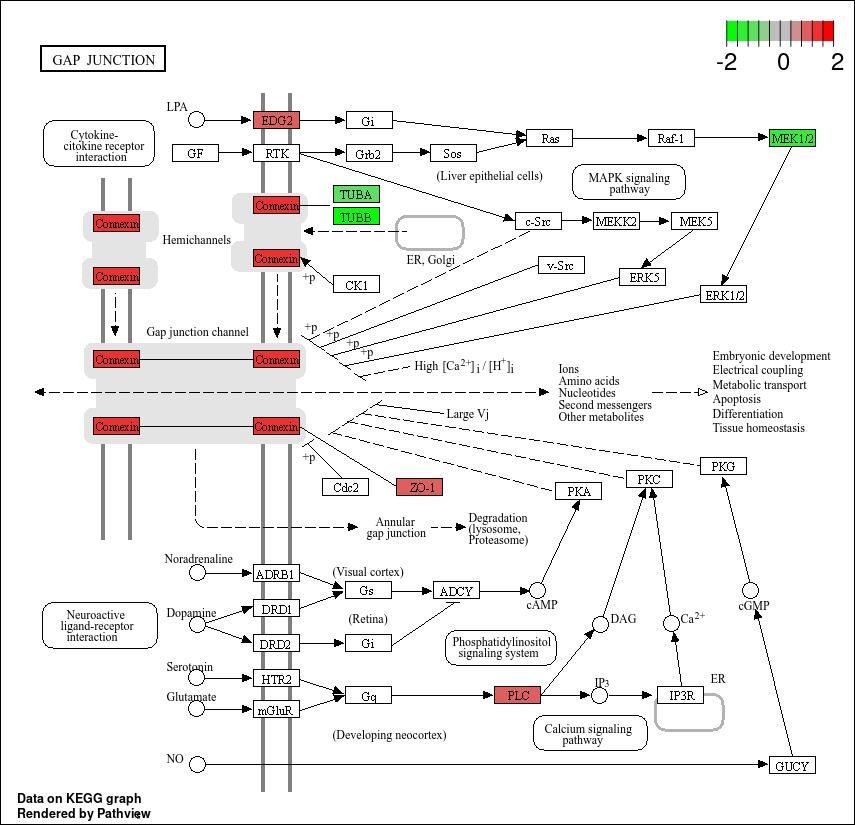

Supplement: Supplementary file 1 [file DataSheet1.zip › 1520845Supplementary files/08差异基因的KEGG富集分析/pathway.d3f24d25e5508754/hsa04540.pathview.png]

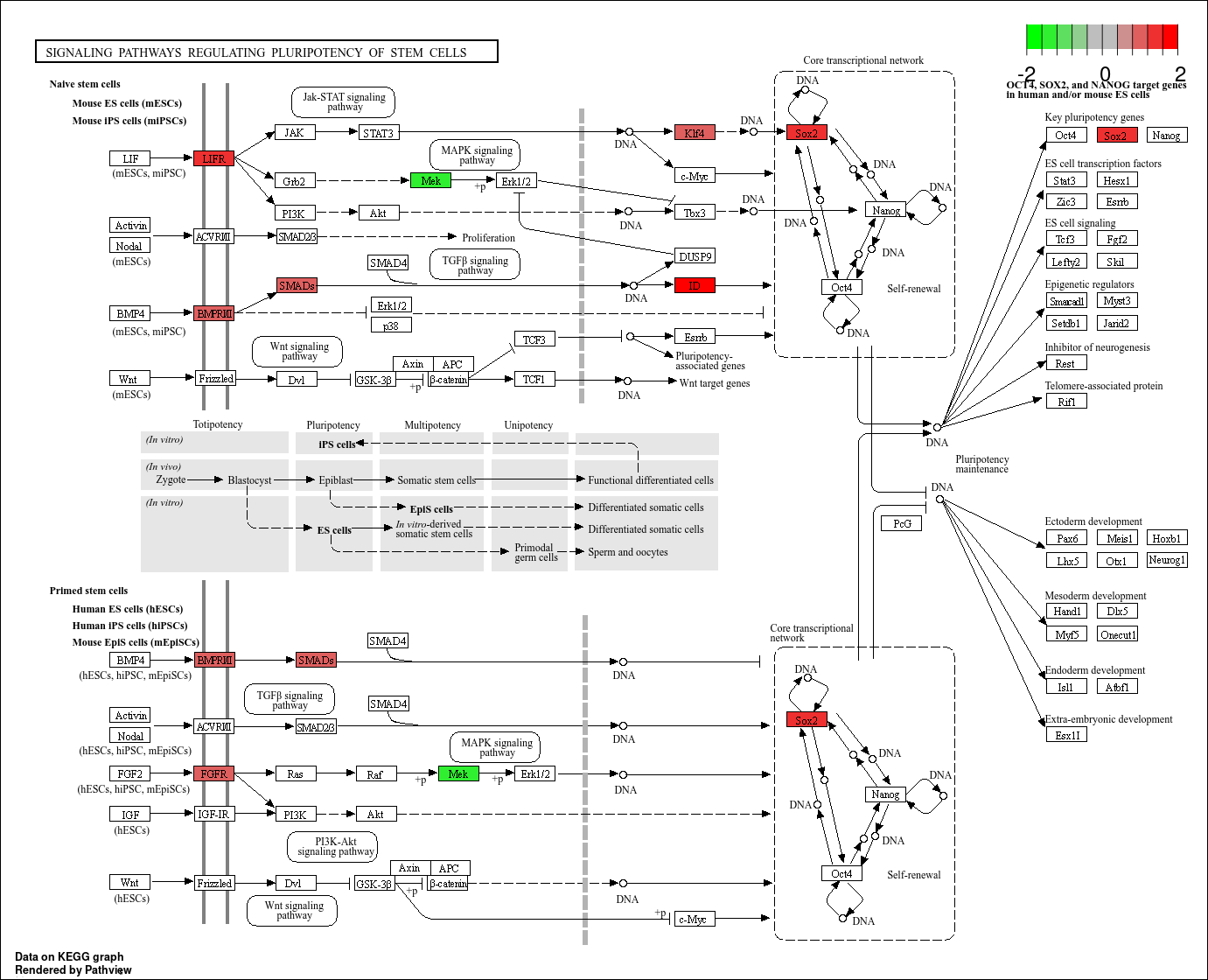

Supplement: Supplementary file 1 [file DataSheet1.zip › 1520845Supplementary files/08差异基因的KEGG富集分析/pathway.d3f24d25e5508754/hsa04550.pathview.png]

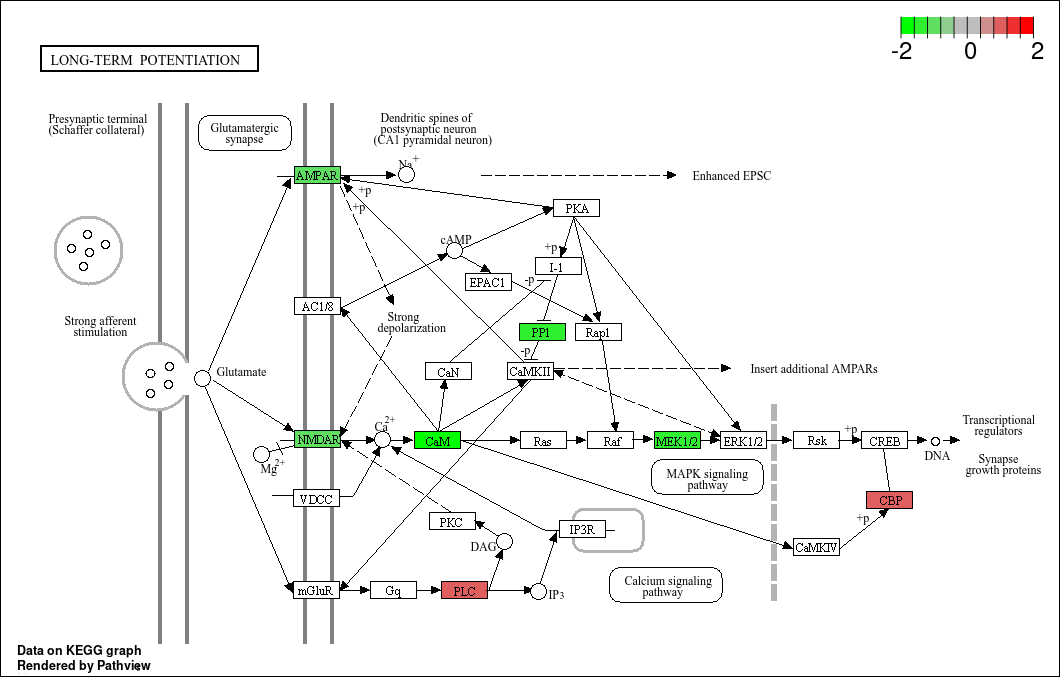

Supplement: Supplementary file 1 [file DataSheet1.zip › 1520845Supplementary files/08差异基因的KEGG富集分析/pathway.d3f24d25e5508754/hsa04720.pathview.png]

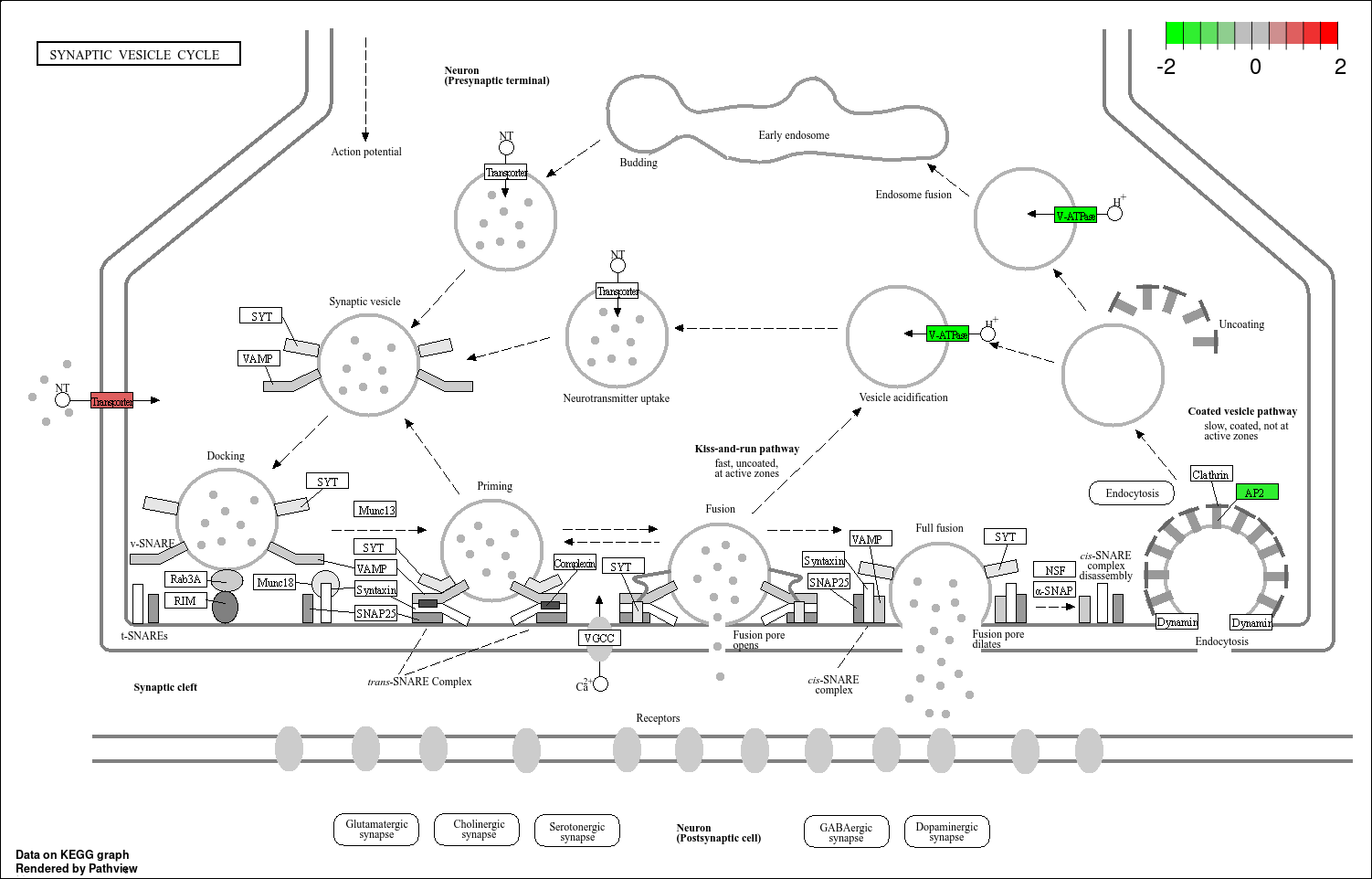

Supplement: Supplementary file 1 [file DataSheet1.zip › 1520845Supplementary files/08差异基因的KEGG富集分析/pathway.d3f24d25e5508754/hsa04721.pathview.png]

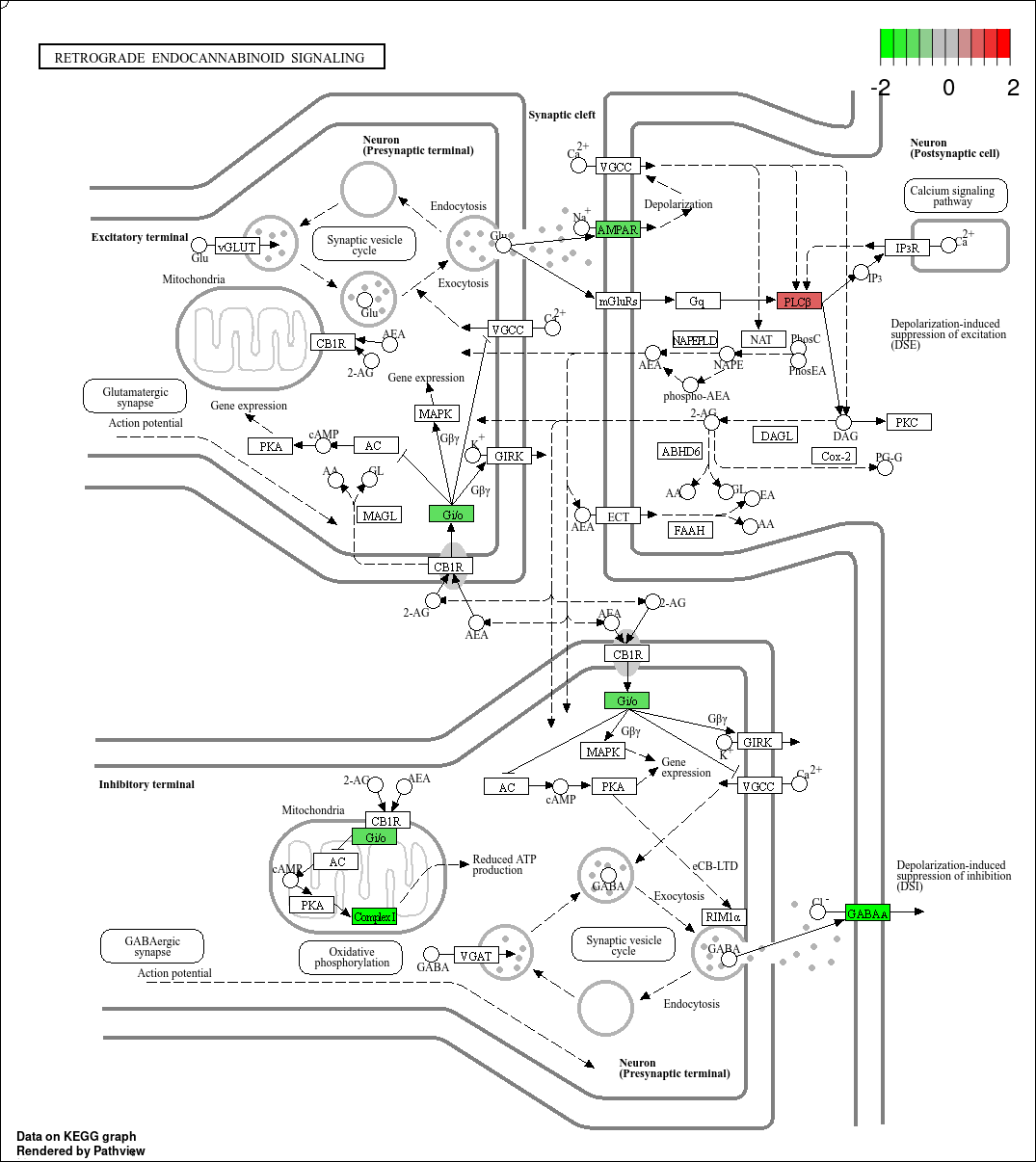

Supplement: Supplementary file 1 [file DataSheet1.zip › 1520845Supplementary files/08差异基因的KEGG富集分析/pathway.d3f24d25e5508754/hsa04723.pathview.png]

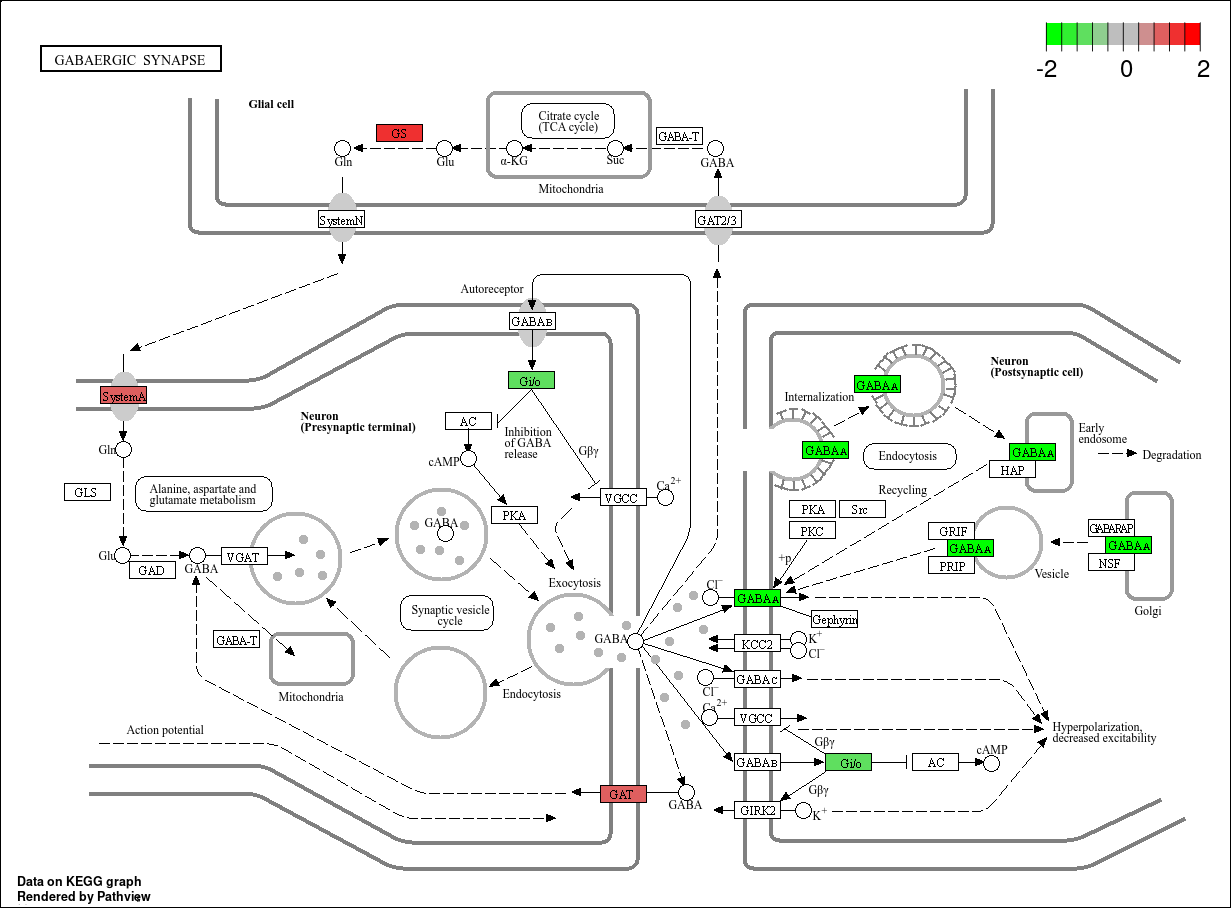

Supplement: Supplementary file 1 [file DataSheet1.zip › 1520845Supplementary files/08差异基因的KEGG富集分析/pathway.d3f24d25e5508754/hsa04727.pathview.png]

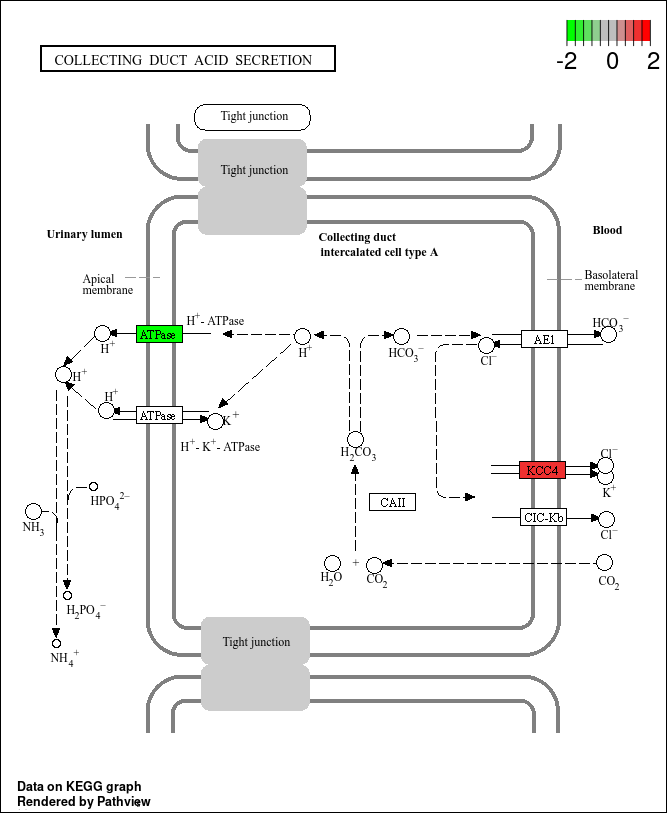

Supplement: Supplementary file 1 [file DataSheet1.zip › 1520845Supplementary files/08差异基因的KEGG富集分析/pathway.d3f24d25e5508754/hsa04966.pathview.png]

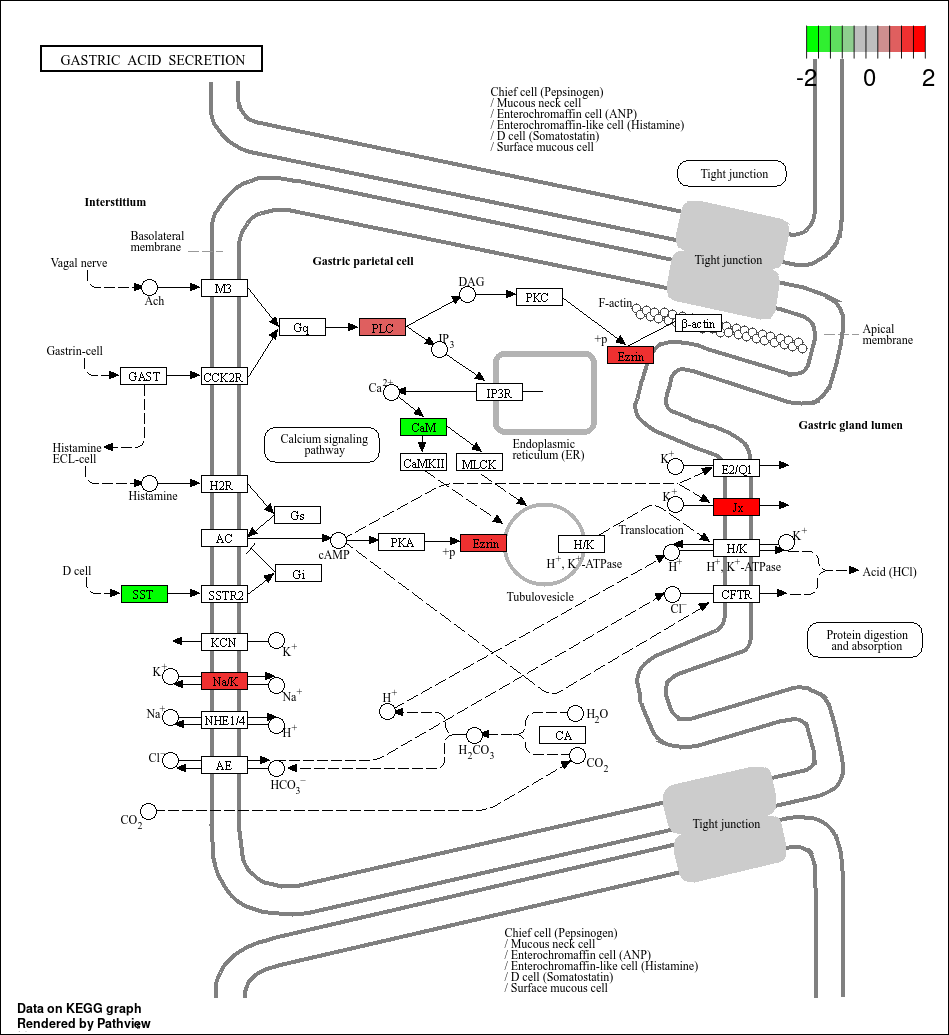

Supplement: Supplementary file 1 [file DataSheet1.zip › 1520845Supplementary files/08差异基因的KEGG富集分析/pathway.d3f24d25e5508754/hsa04971.pathview.png]

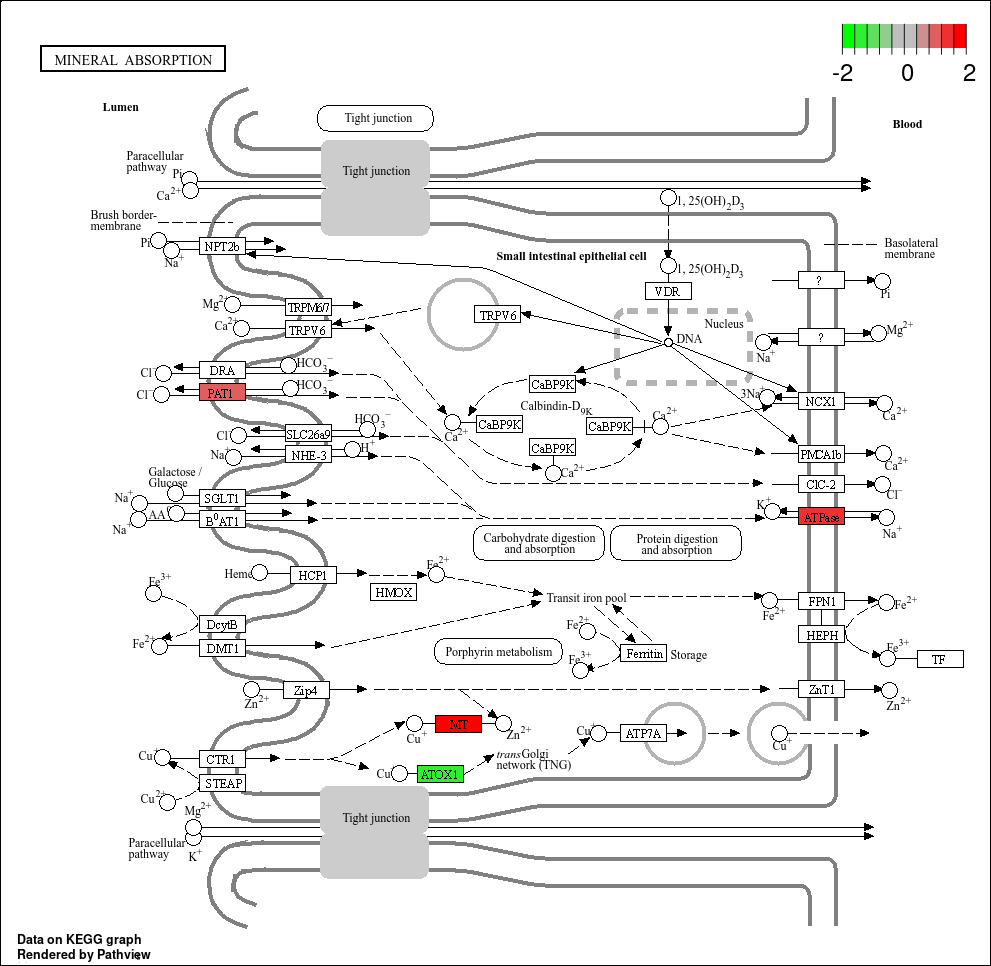

Supplement: Supplementary file 1 [file DataSheet1.zip › 1520845Supplementary files/08差异基因的KEGG富集分析/pathway.d3f24d25e5508754/hsa04978.pathview.png]

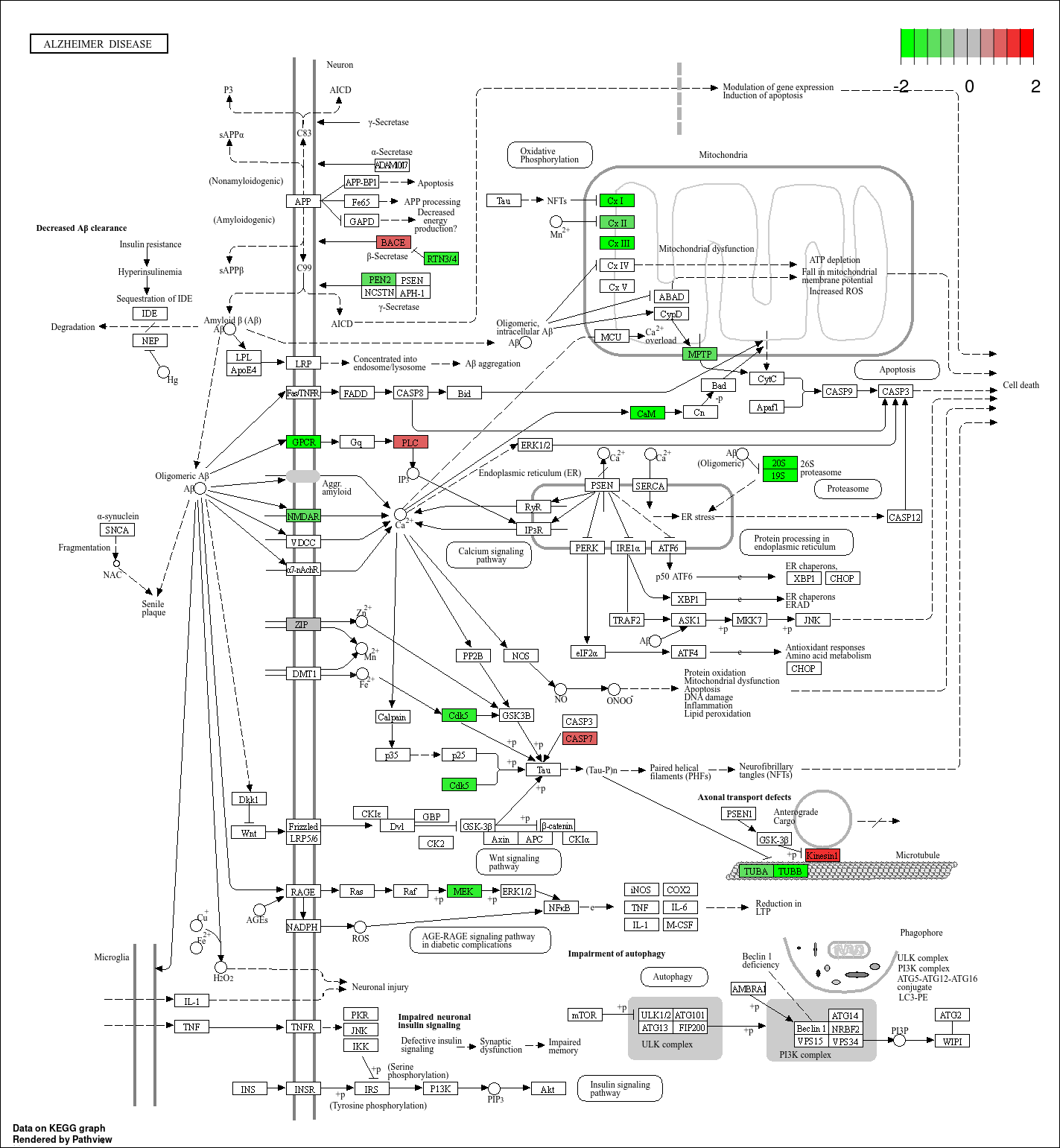

Supplement: Supplementary file 1 [file DataSheet1.zip › 1520845Supplementary files/08差异基因的KEGG富集分析/pathway.d3f24d25e5508754/hsa05010.pathview.png]

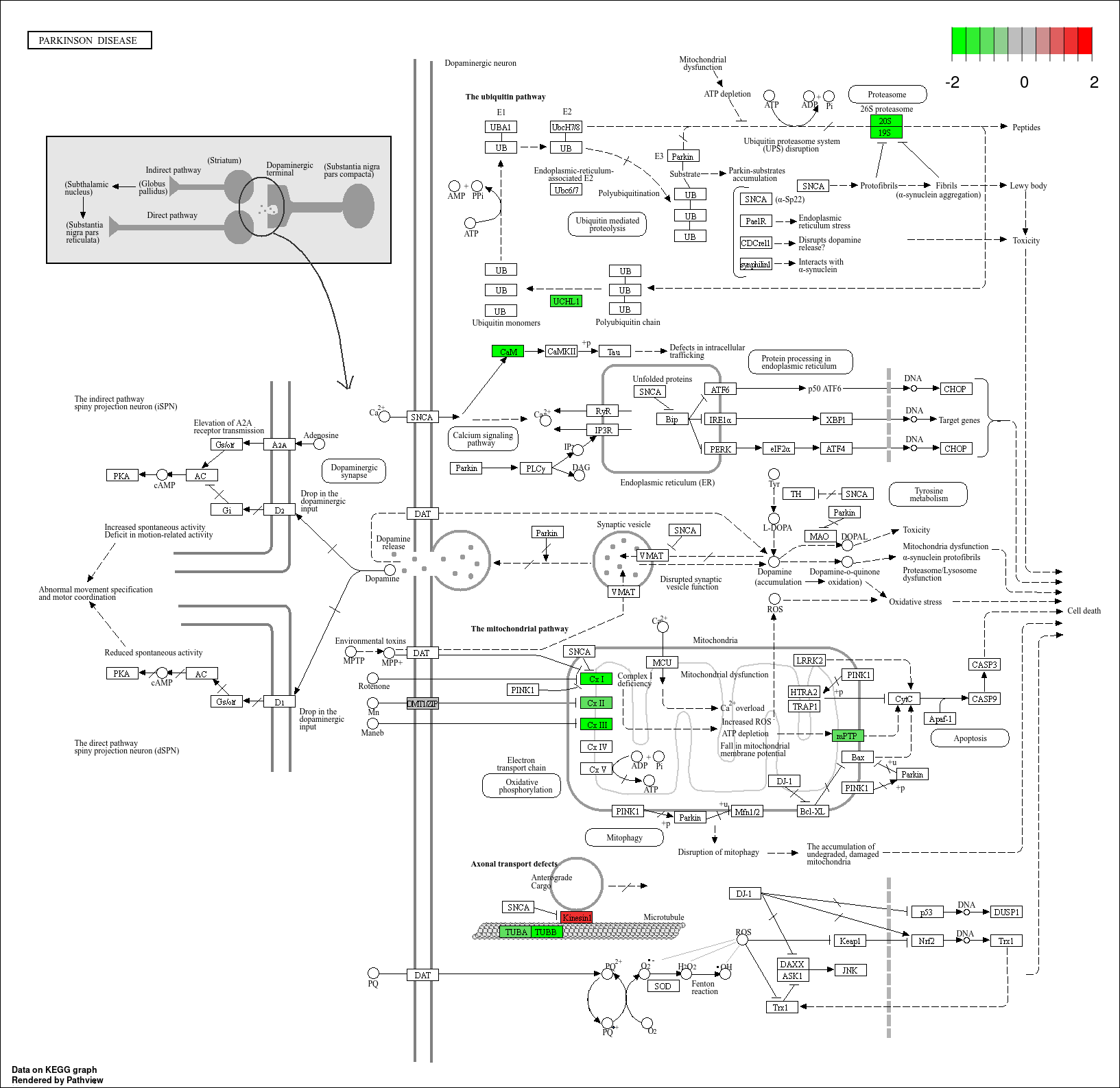

Supplement: Supplementary file 1 [file DataSheet1.zip › 1520845Supplementary files/08差异基因的KEGG富集分析/pathway.d3f24d25e5508754/hsa05012.pathview.png]

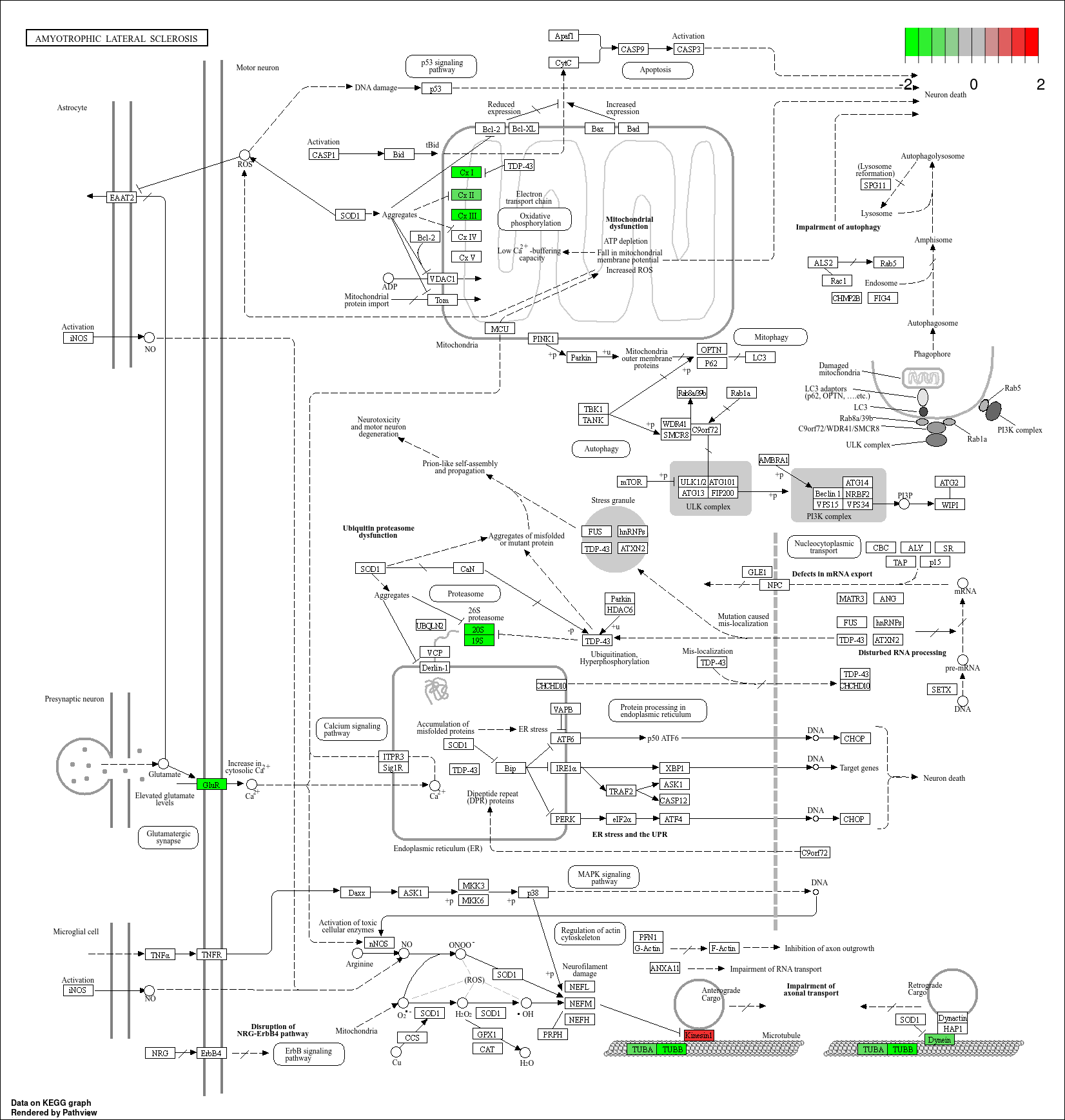

Supplement: Supplementary file 1 [file DataSheet1.zip › 1520845Supplementary files/08差异基因的KEGG富集分析/pathway.d3f24d25e5508754/hsa05014.pathview.png]

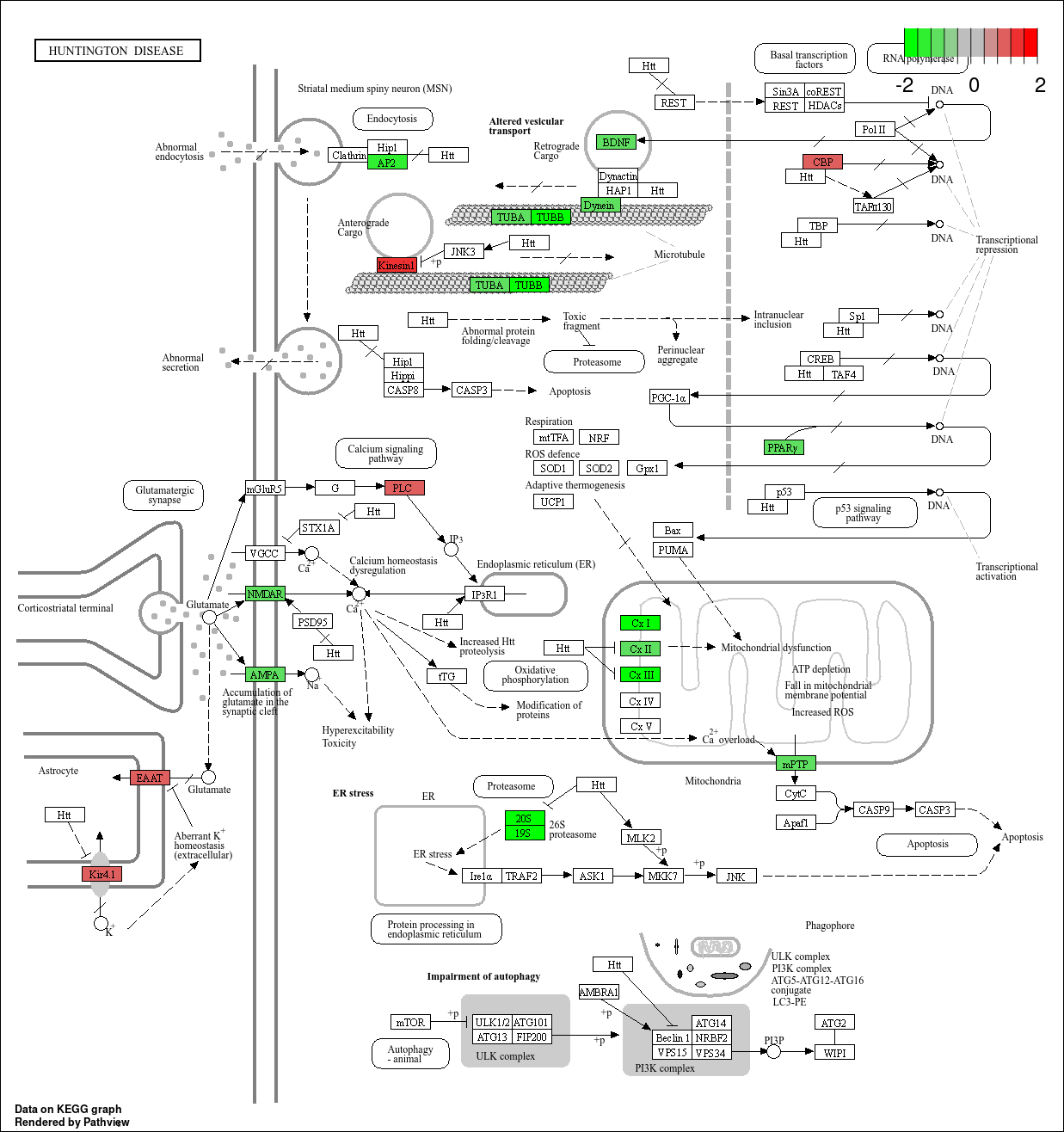

Supplement: Supplementary file 1 [file DataSheet1.zip › 1520845Supplementary files/08差异基因的KEGG富集分析/pathway.d3f24d25e5508754/hsa05016.pathview.png]

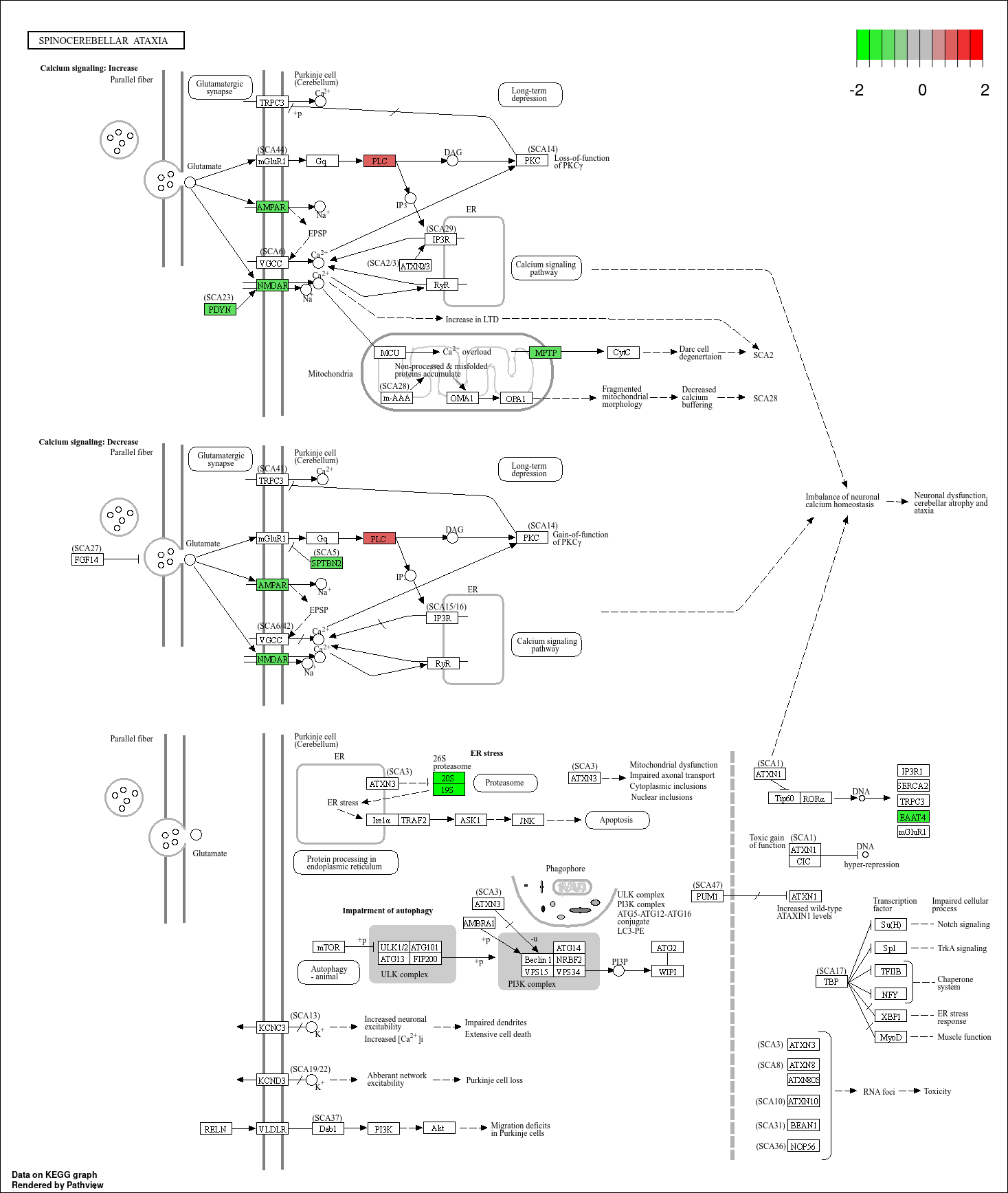

Supplement: Supplementary file 1 [file DataSheet1.zip › 1520845Supplementary files/08差异基因的KEGG富集分析/pathway.d3f24d25e5508754/hsa05017.pathview.png]

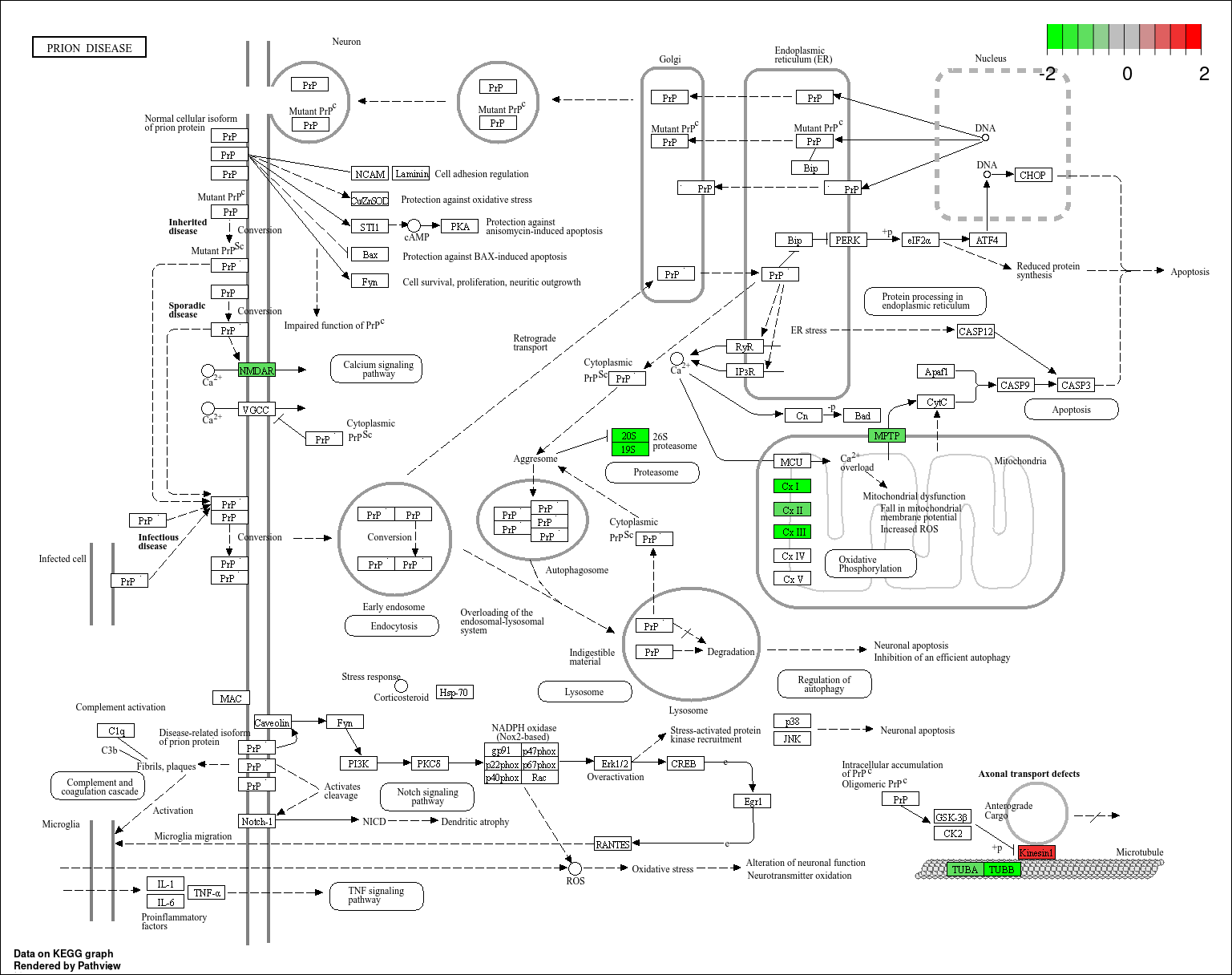

Supplement: Supplementary file 1 [file DataSheet1.zip › 1520845Supplementary files/08差异基因的KEGG富集分析/pathway.d3f24d25e5508754/hsa05020.pathview.png]

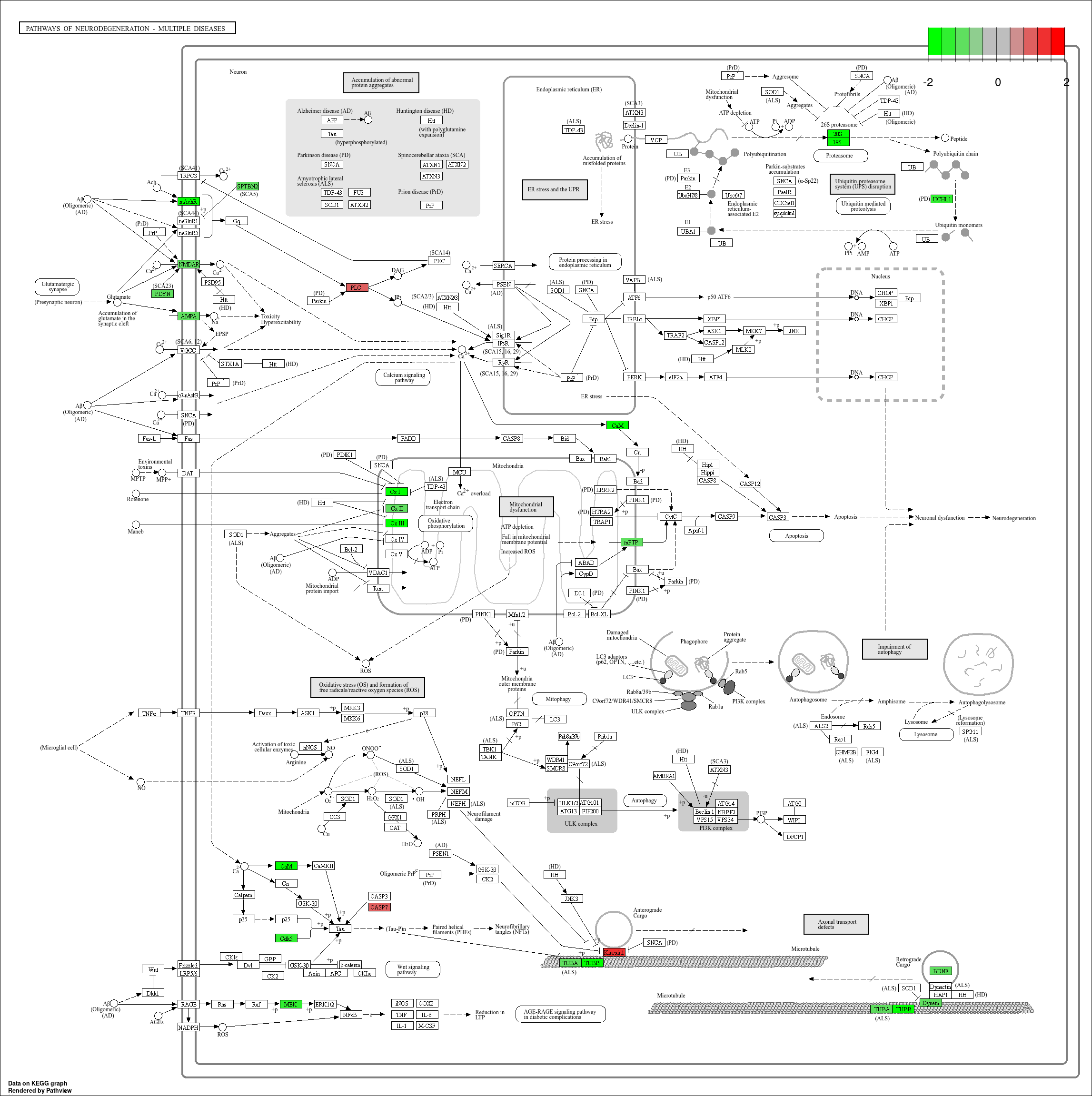

Supplement: Supplementary file 1 [file DataSheet1.zip › 1520845Supplementary files/08差异基因的KEGG富集分析/pathway.d3f24d25e5508754/hsa05022.pathview.png]

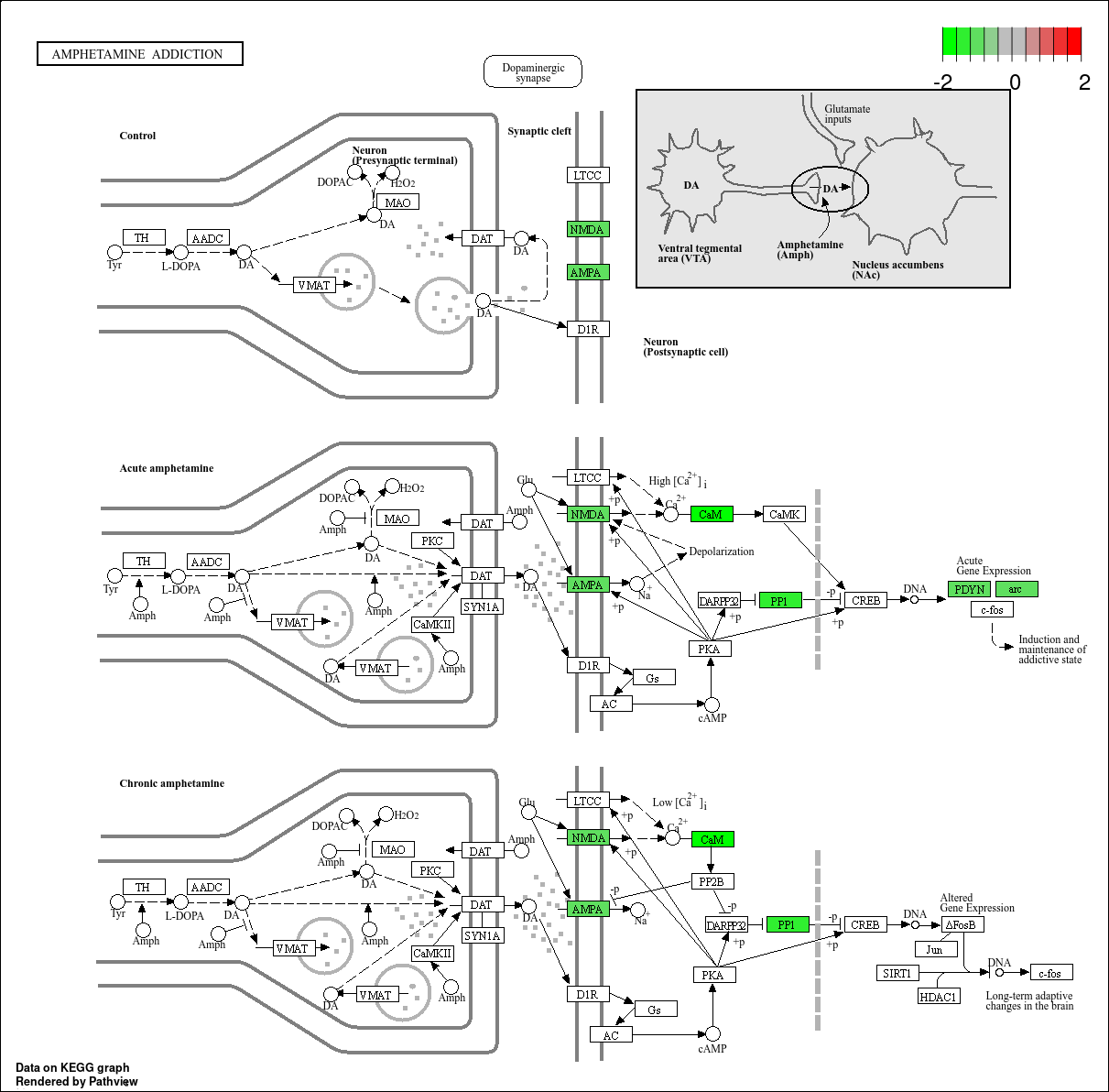

Supplement: Supplementary file 1 [file DataSheet1.zip › 1520845Supplementary files/08差异基因的KEGG富集分析/pathway.d3f24d25e5508754/hsa05031.pathview.png]

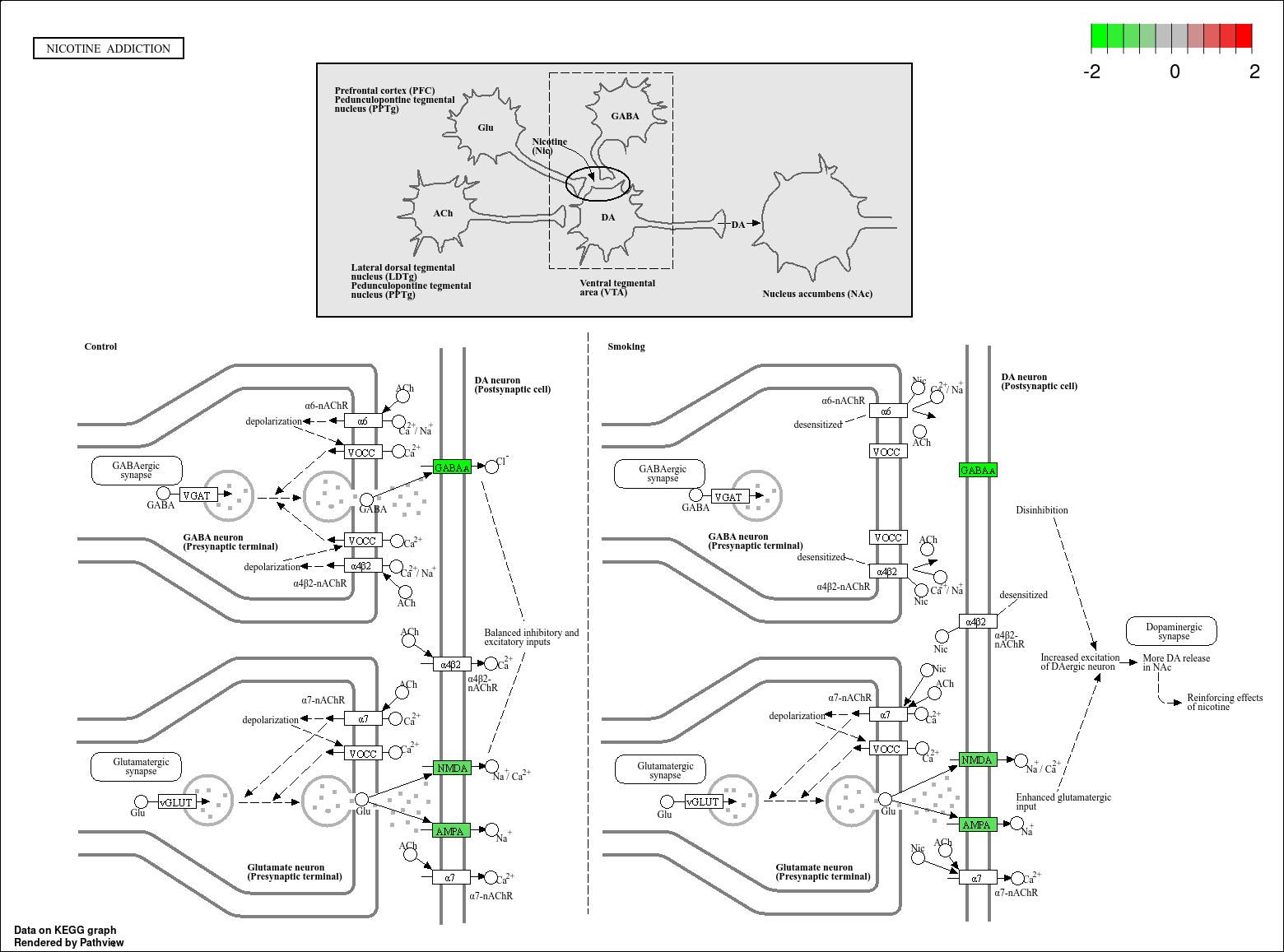

Supplement: Supplementary file 1 [file DataSheet1.zip › 1520845Supplementary files/08差异基因的KEGG富集分析/pathway.d3f24d25e5508754/hsa05033.pathview.png]

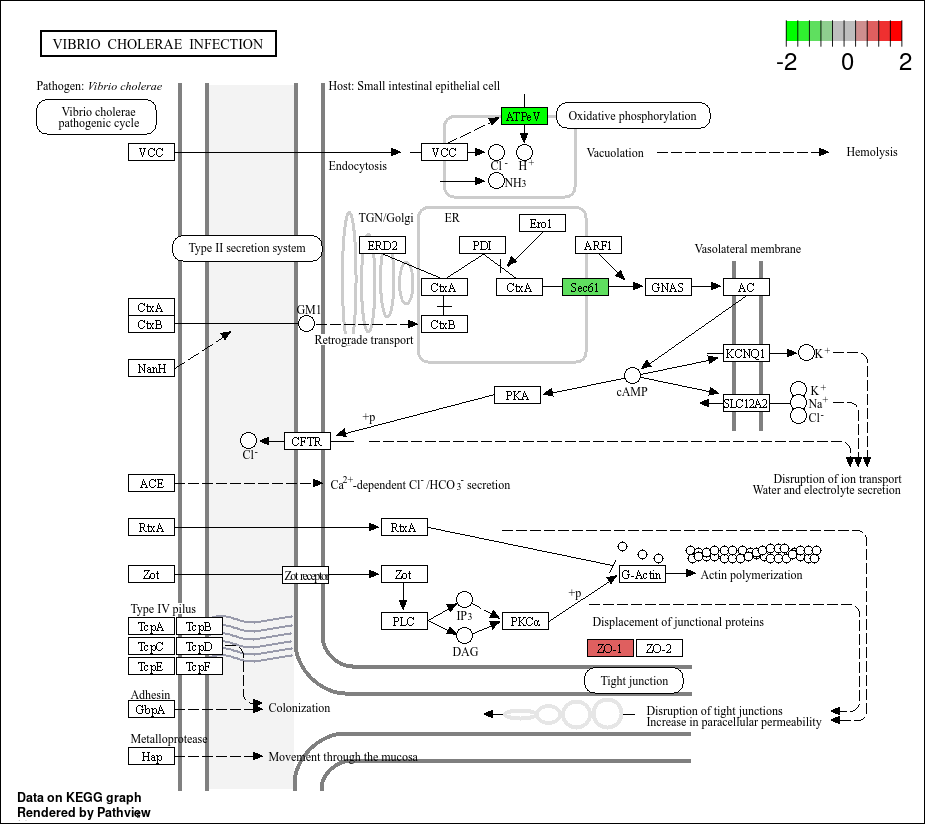

Supplement: Supplementary file 1 [file DataSheet1.zip › 1520845Supplementary files/08差异基因的KEGG富集分析/pathway.d3f24d25e5508754/hsa05110.pathview.png]

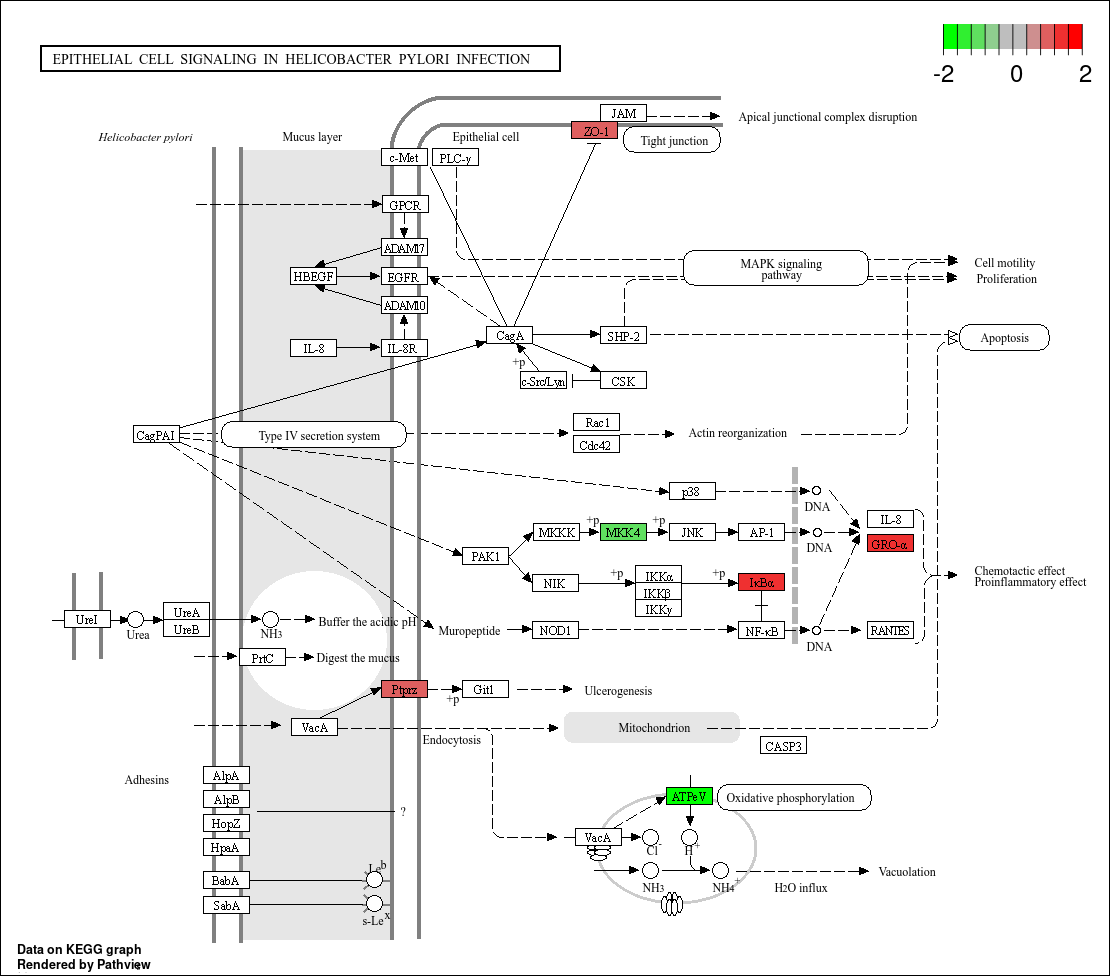

Supplement: Supplementary file 1 [file DataSheet1.zip › 1520845Supplementary files/08差异基因的KEGG富集分析/pathway.d3f24d25e5508754/hsa05120.pathview.png]

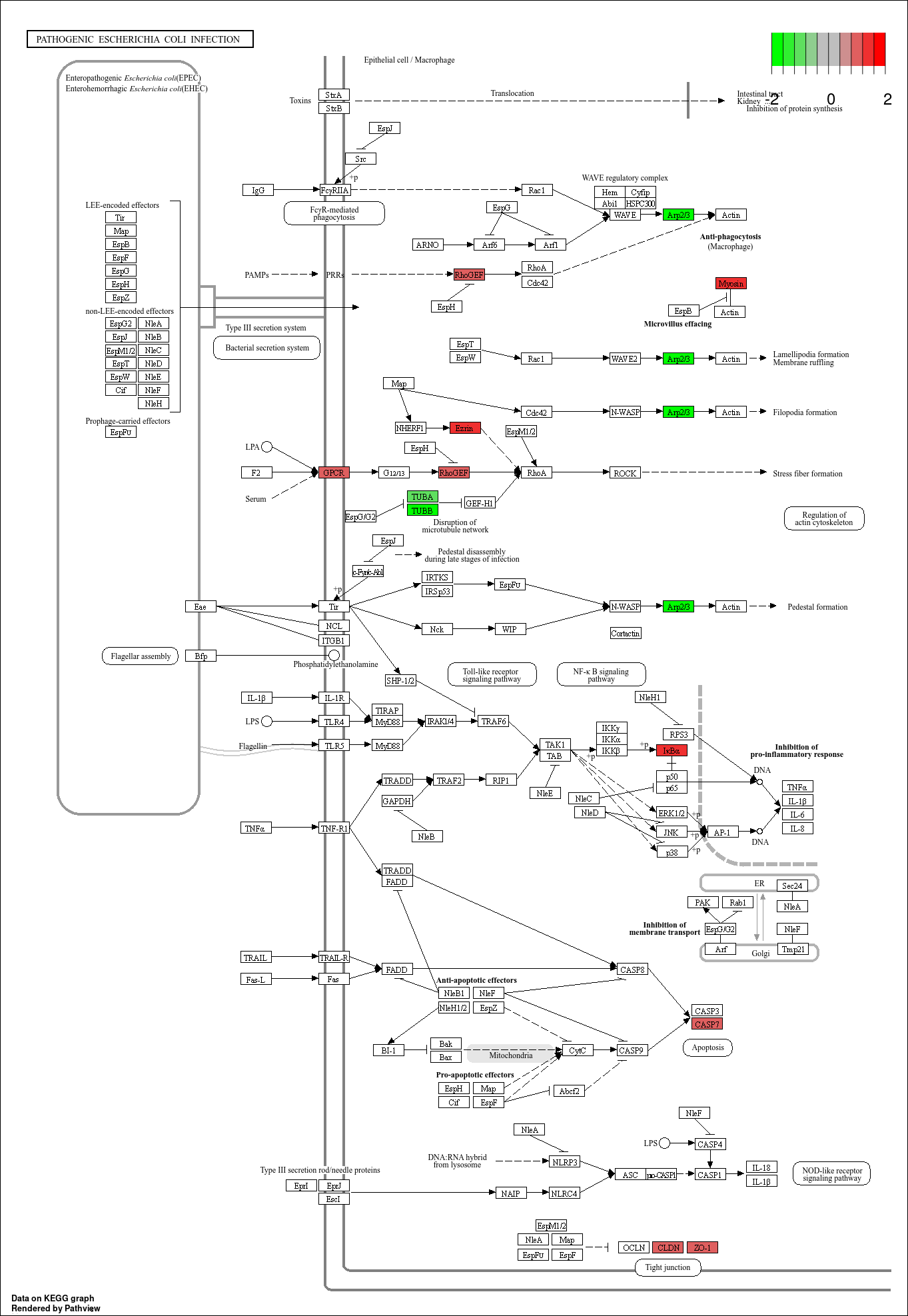

Supplement: Supplementary file 1 [file DataSheet1.zip › 1520845Supplementary files/08差异基因的KEGG富集分析/pathway.d3f24d25e5508754/hsa05130.pathview.png]

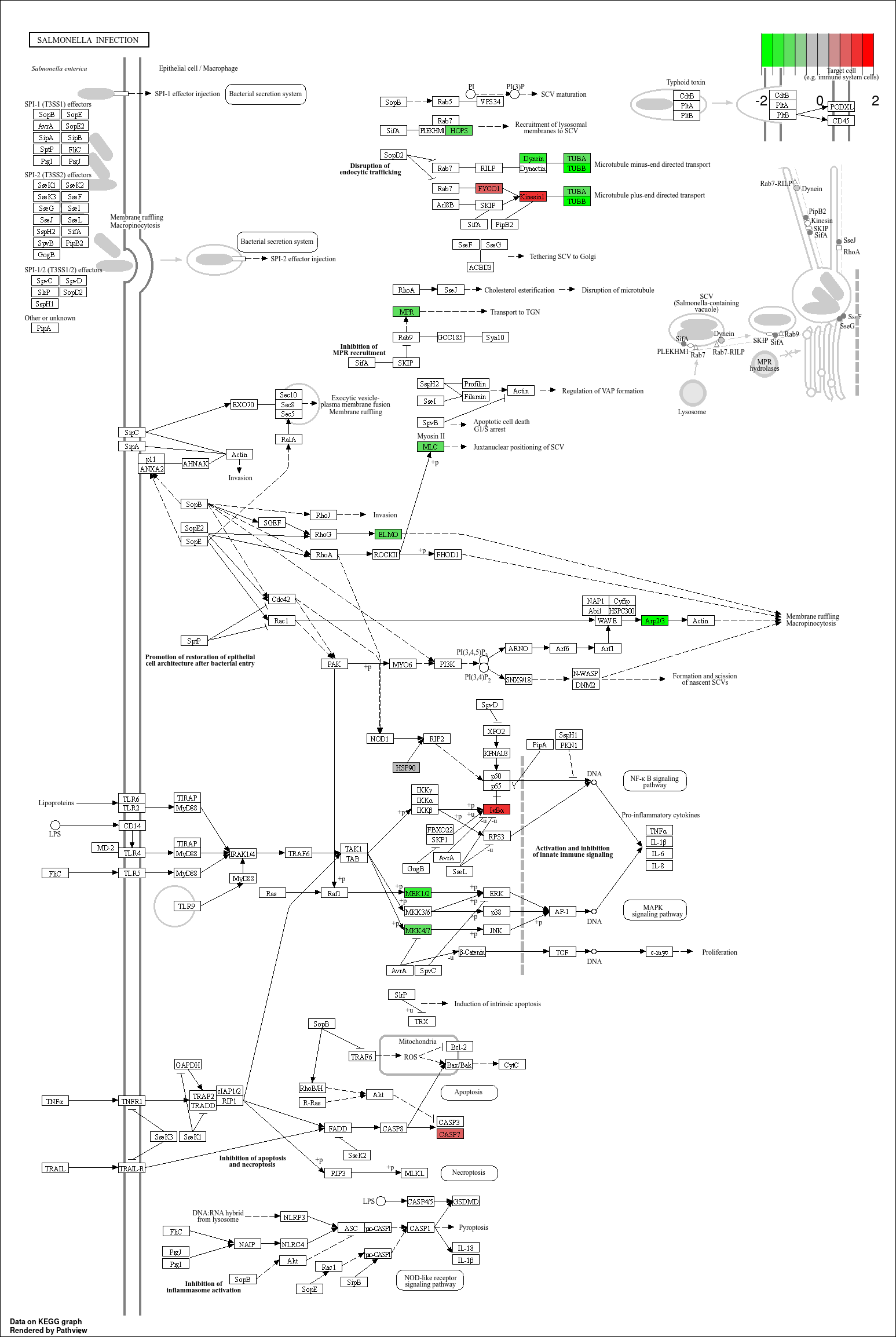

Supplement: Supplementary file 1 [file DataSheet1.zip › 1520845Supplementary files/08差异基因的KEGG富集分析/pathway.d3f24d25e5508754/hsa05132.pathview.png]

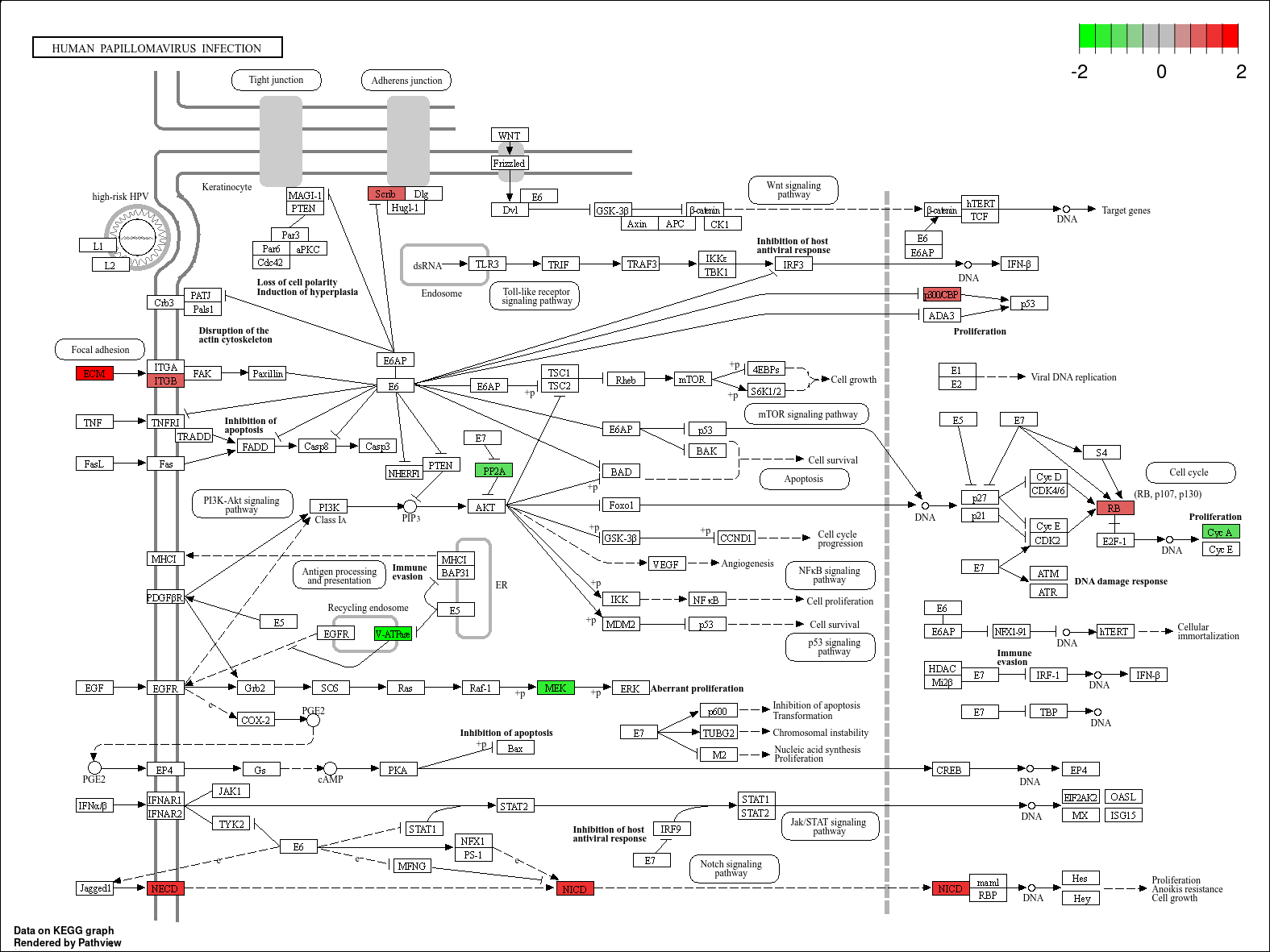

Supplement: Supplementary file 1 [file DataSheet1.zip › 1520845Supplementary files/08差异基因的KEGG富集分析/pathway.d3f24d25e5508754/hsa05165.pathview.png]

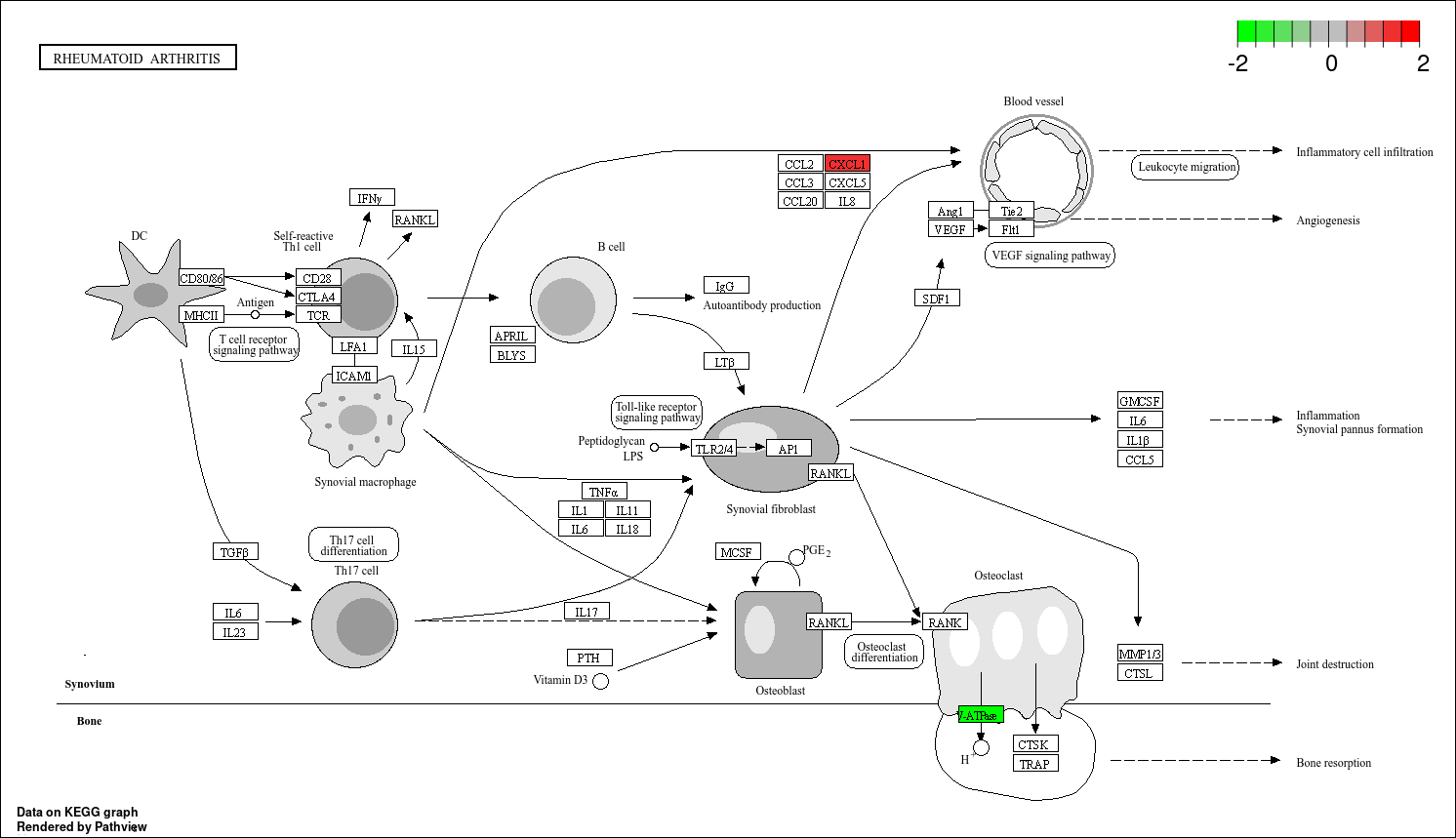

Supplement: Supplementary file 1 [file DataSheet1.zip › 1520845Supplementary files/08差异基因的KEGG富集分析/pathway.d3f24d25e5508754/hsa05323.pathview.png]

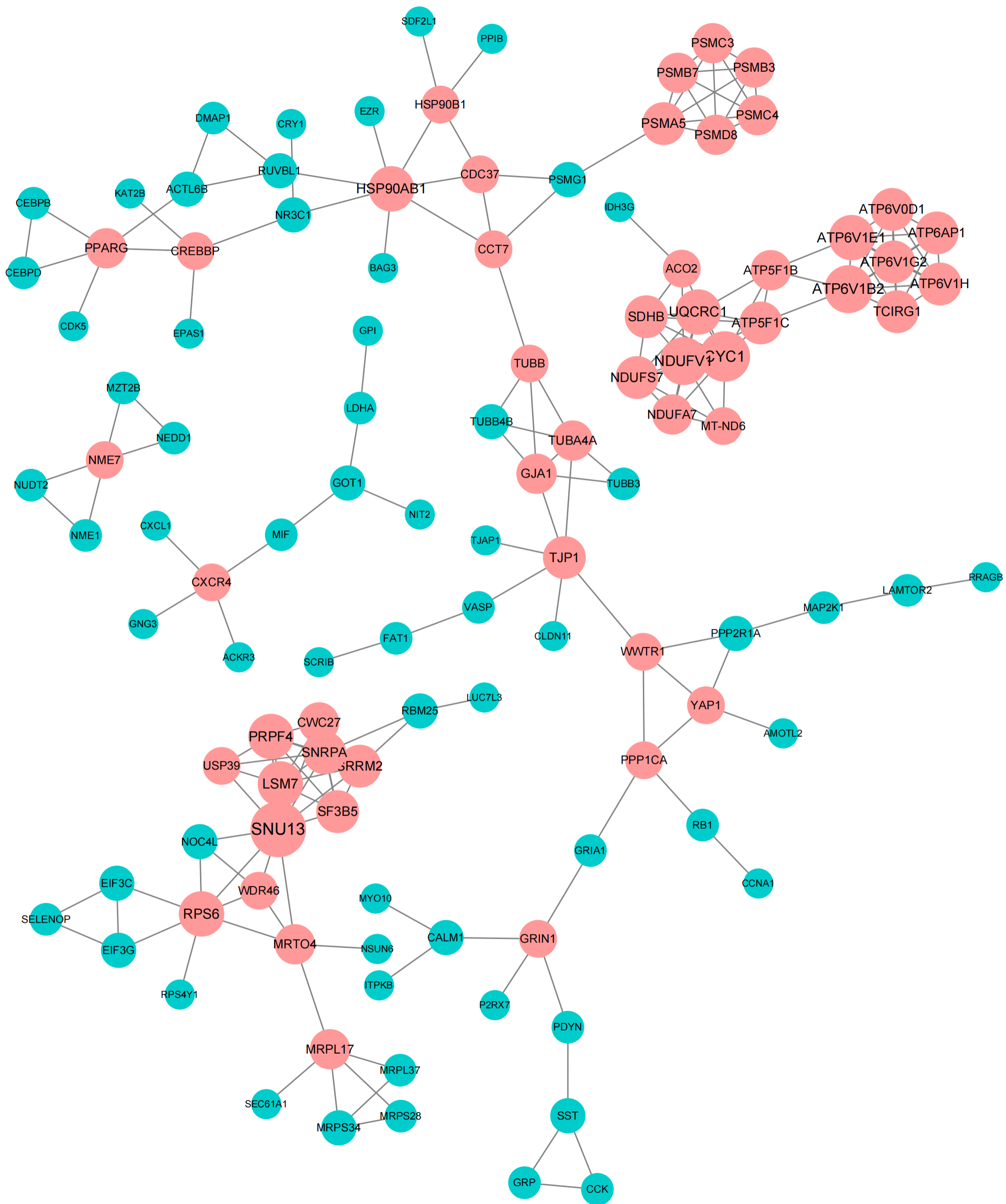

Supplement: Supplementary file 1 [file DataSheet1.zip › 1520845Supplementary files/10蛋白互作网络可视化筛选关键基因cytoscape/string_interactions_short.tsv_00(1).PDF]

## Random forest

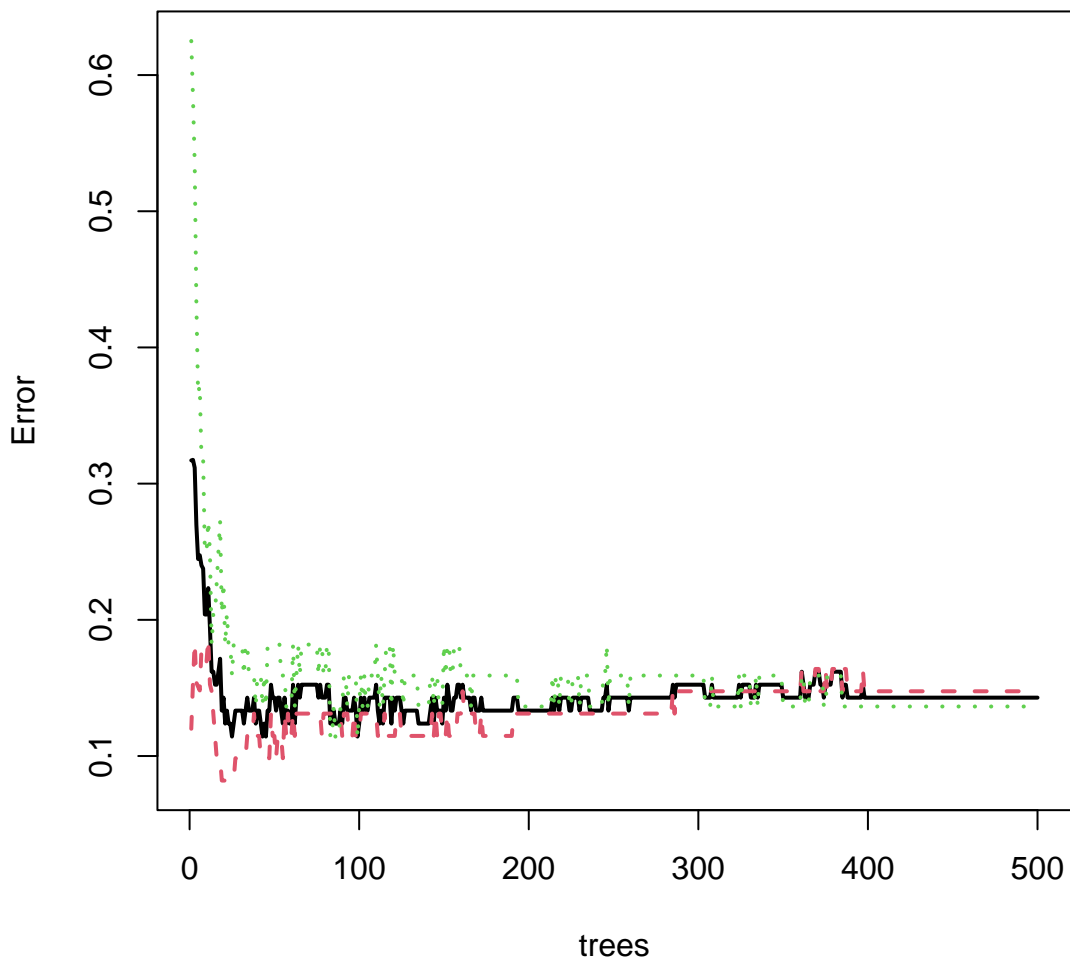

Supplement: Supplementary file 1 [file DataSheet1.zip › 1520845Supplementary files/12随机森林模型识别基因/forest.pdf]

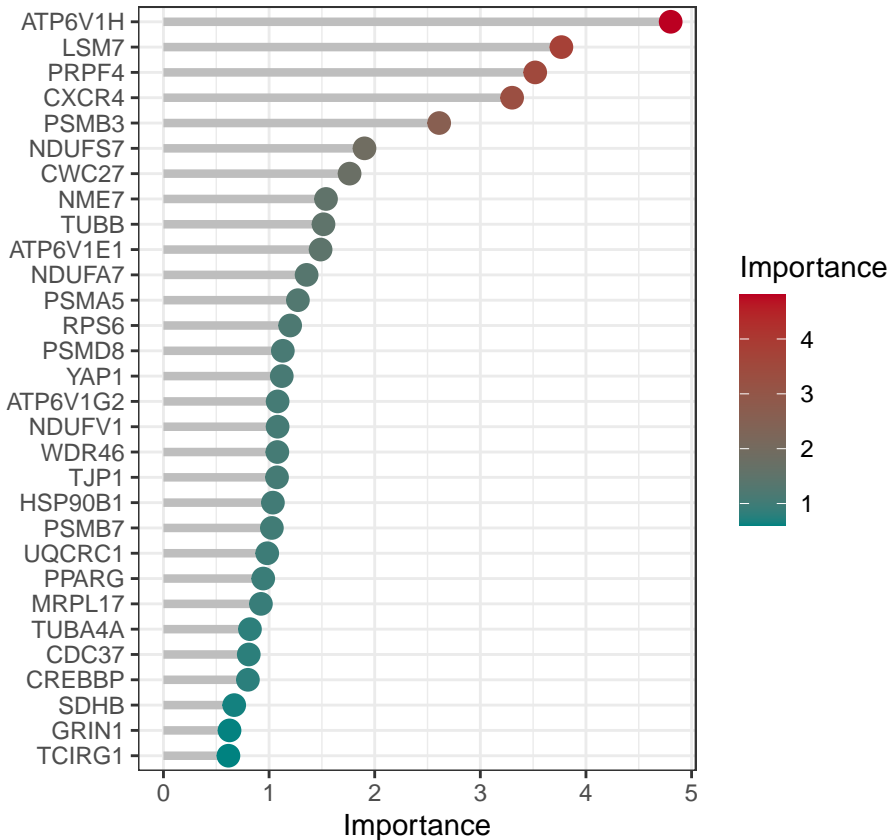

Supplement: Supplementary file 1 [file DataSheet1.zip › 1520845Supplementary files/12随机森林模型识别基因/geneImportance.pdf]

Binomial Deviance

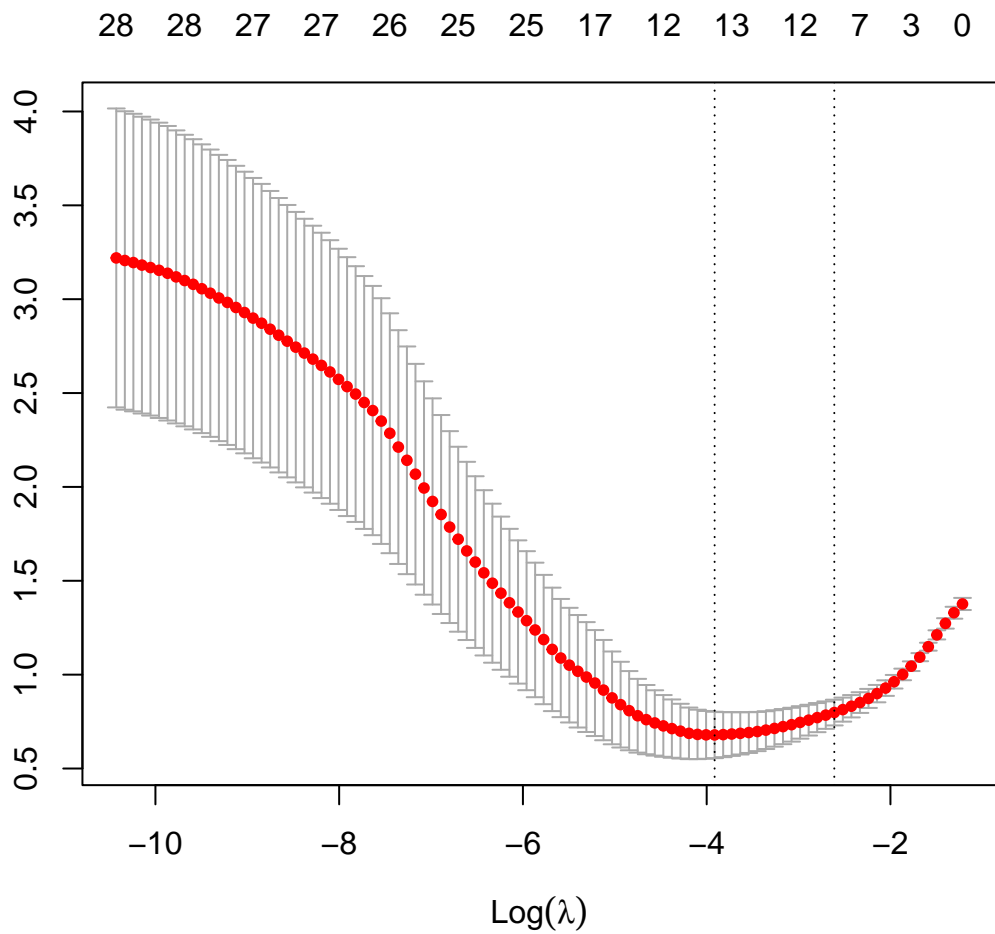

Supplement: Supplementary file 1 [file DataSheet1.zip › 1520845Supplementary files/13lasso回归识别关键基因/cvfit.pdf]

Coefficients

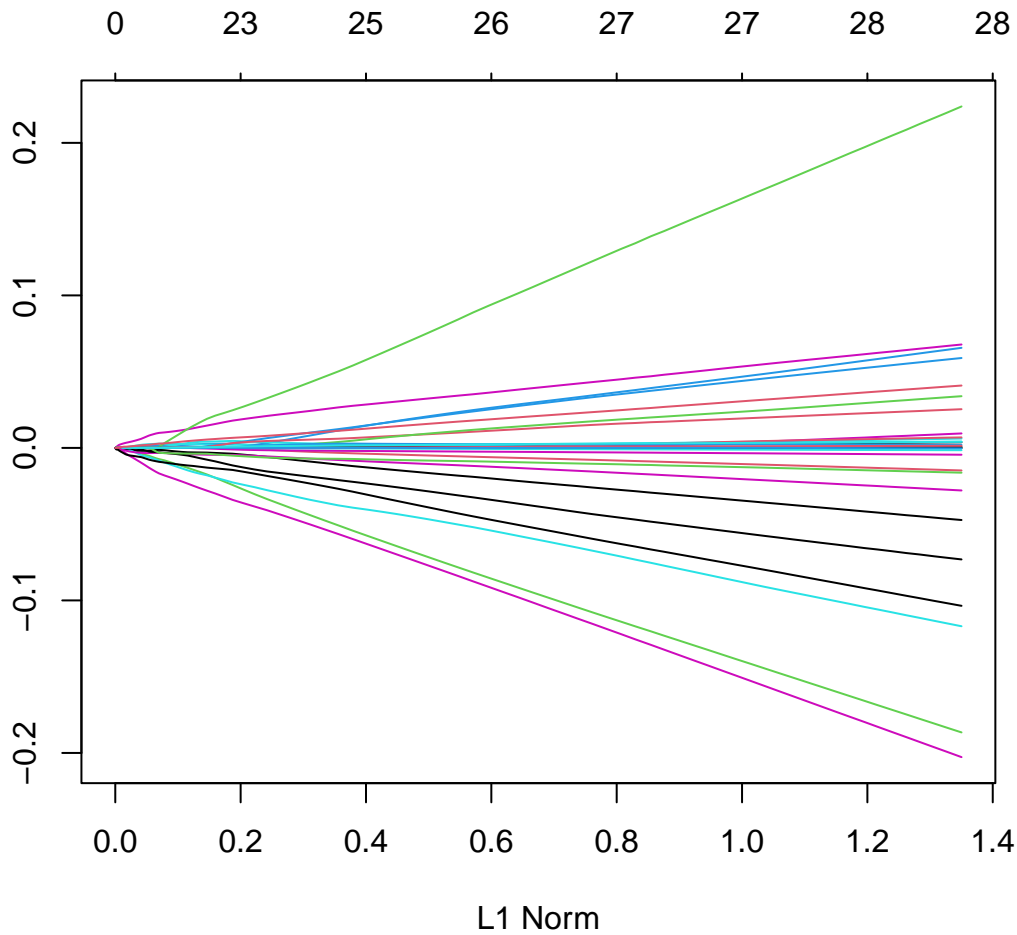

Supplement: Supplementary file 1 [file DataSheet1.zip › 1520845Supplementary files/13lasso回归识别关键基因/lasso.pdf]

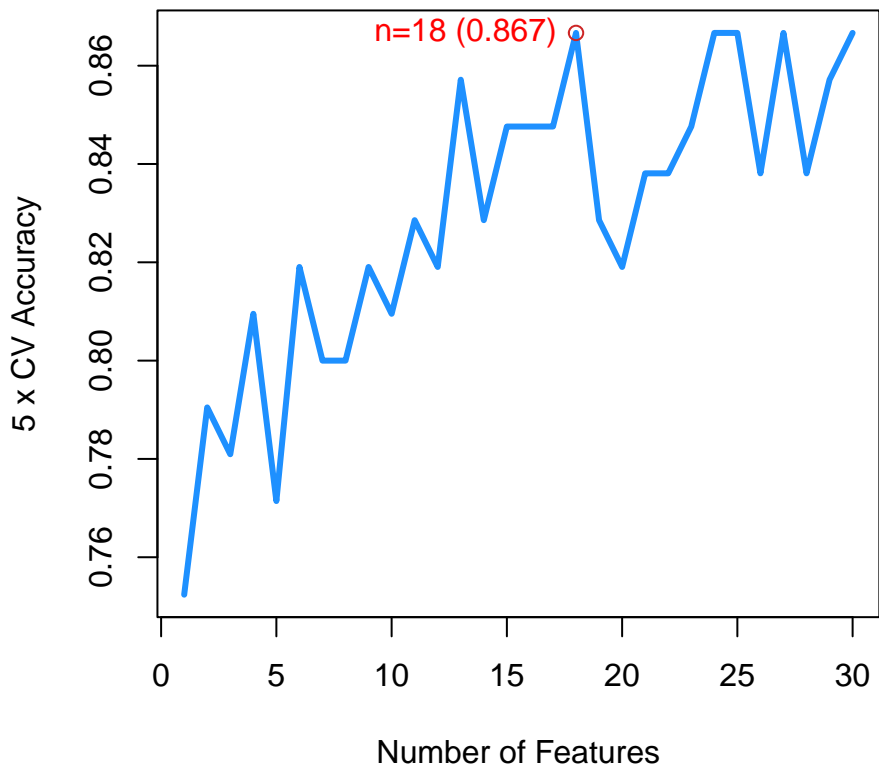

Supplement: Supplementary file 1 [file DataSheet1.zip › 1520845Supplementary files/14SVM-RFE筛选特征基因/accuracy.pdf]

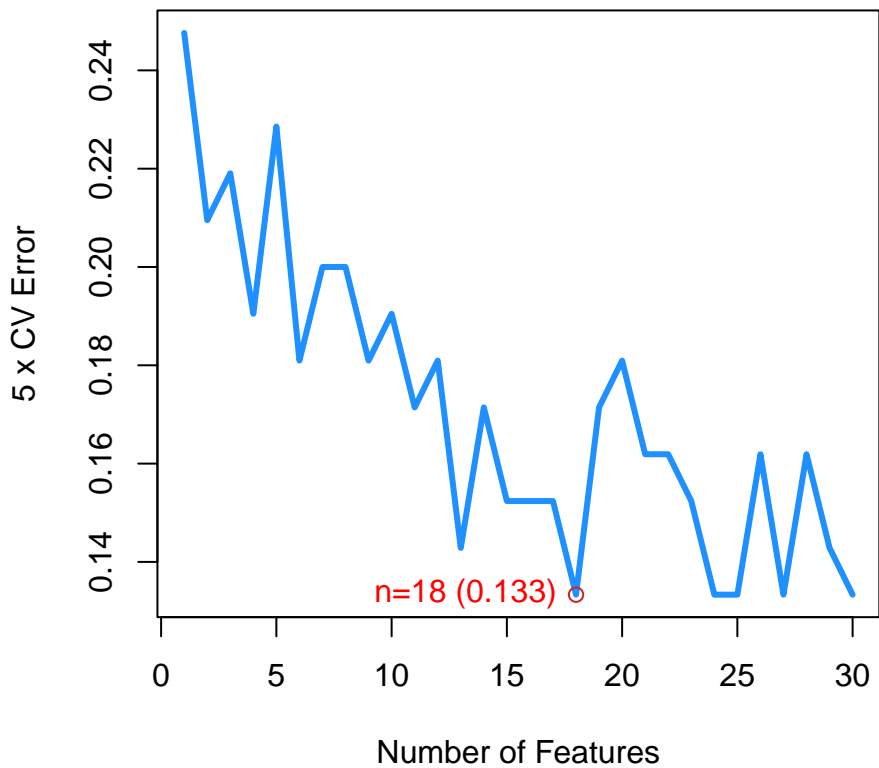

Supplement: Supplementary file 1 [file DataSheet1.zip › 1520845Supplementary files/14SVM-RFE筛选特征基因/errors.pdf]

LASSO

RF

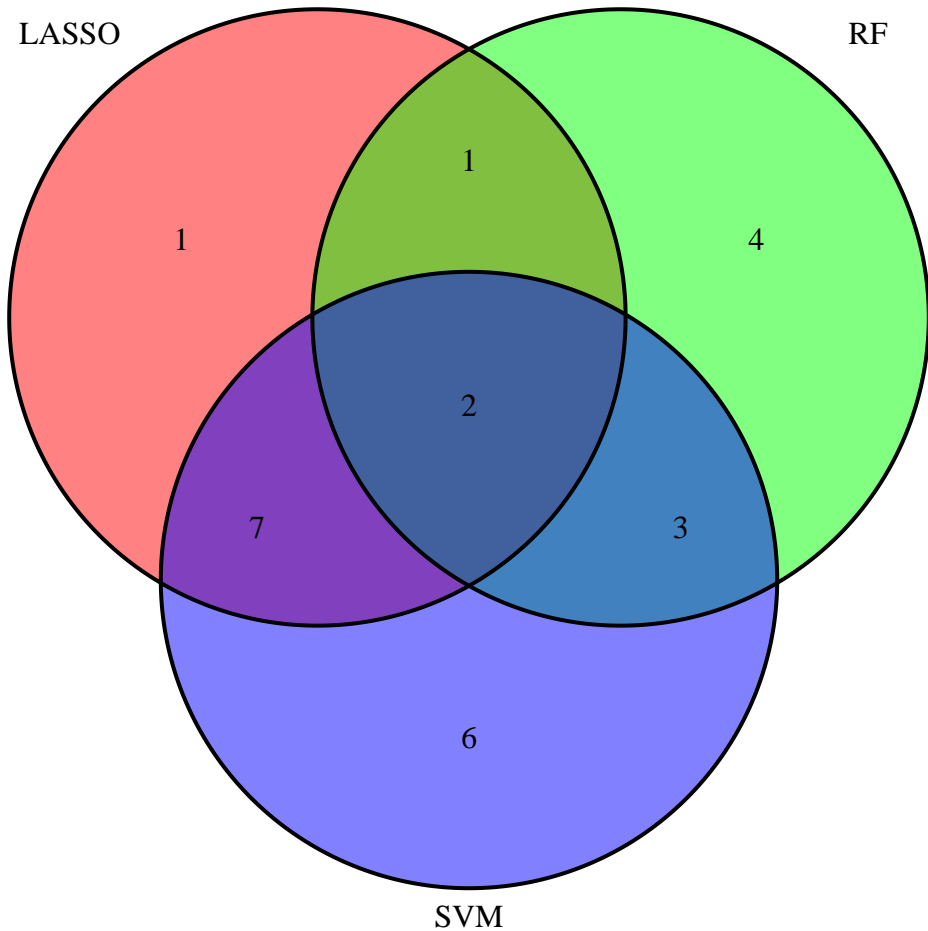

SVM

Supplement: Supplementary file 1 [file DataSheet1.zip › 1520845Supplementary files/15随机森林、SVM-RFE和lasso三者取交集/venn.pdf]

# CXCR4

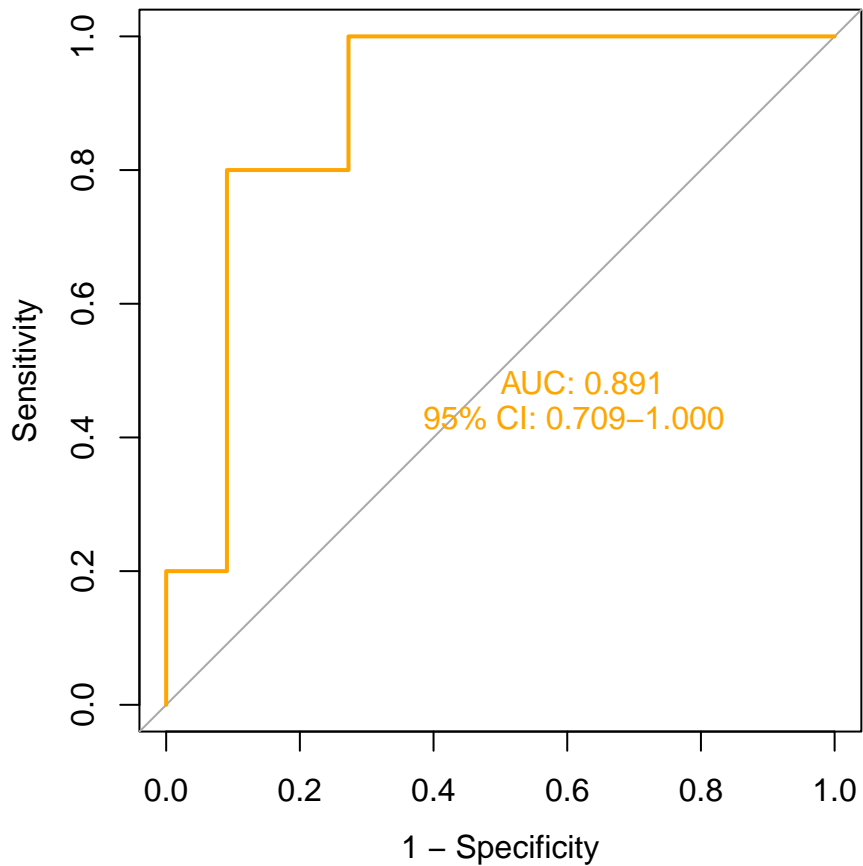

Supplement: Supplementary file 1 [file DataSheet1.zip › 1520845Supplementary files/16识别核心基因的差异分析箱线图及ROC/GSE199939.pdf]

# CXCR4

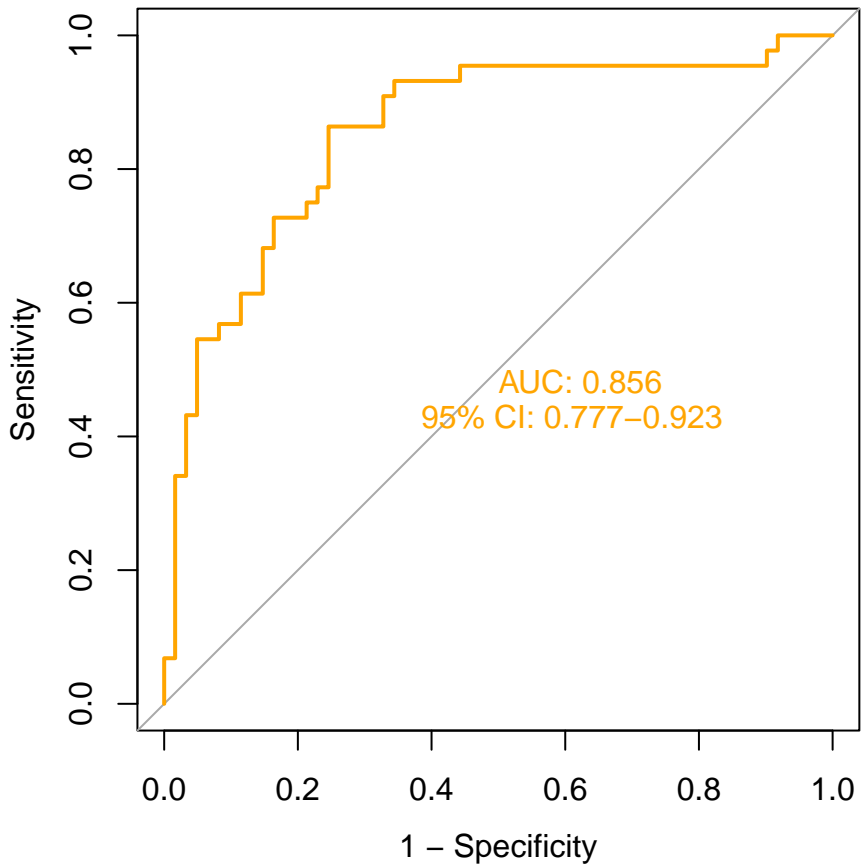

Supplement: Supplementary file 1 [file DataSheet1.zip › 1520845Supplementary files/16识别核心基因的差异分析箱线图及ROC/ROC.CXCR4.pdf]

# CXCR4

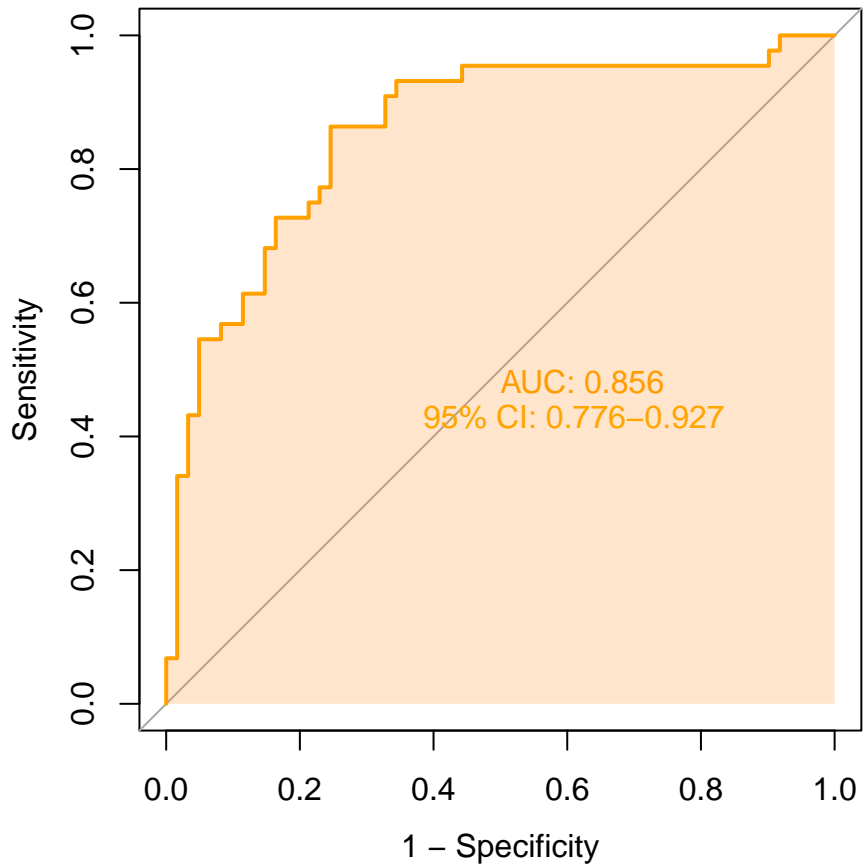

Supplement: Supplementary file 1 [file DataSheet1.zip › 1520845Supplementary files/16识别核心基因的差异分析箱线图及ROC/ROC1.CXCR4.pdf]

Con Treat

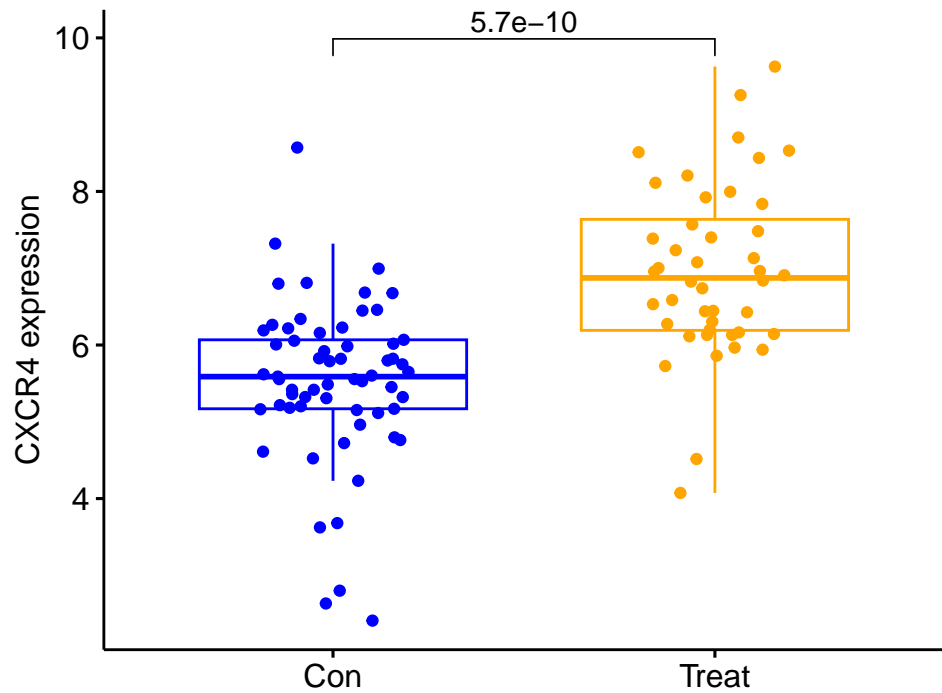

Supplement: Supplementary file 1 [file DataSheet1.zip › 1520845Supplementary files/16识别核心基因的差异分析箱线图及ROC/boxplot.CXCR4.pdf]

# CXCR4 Downregulated Pathways

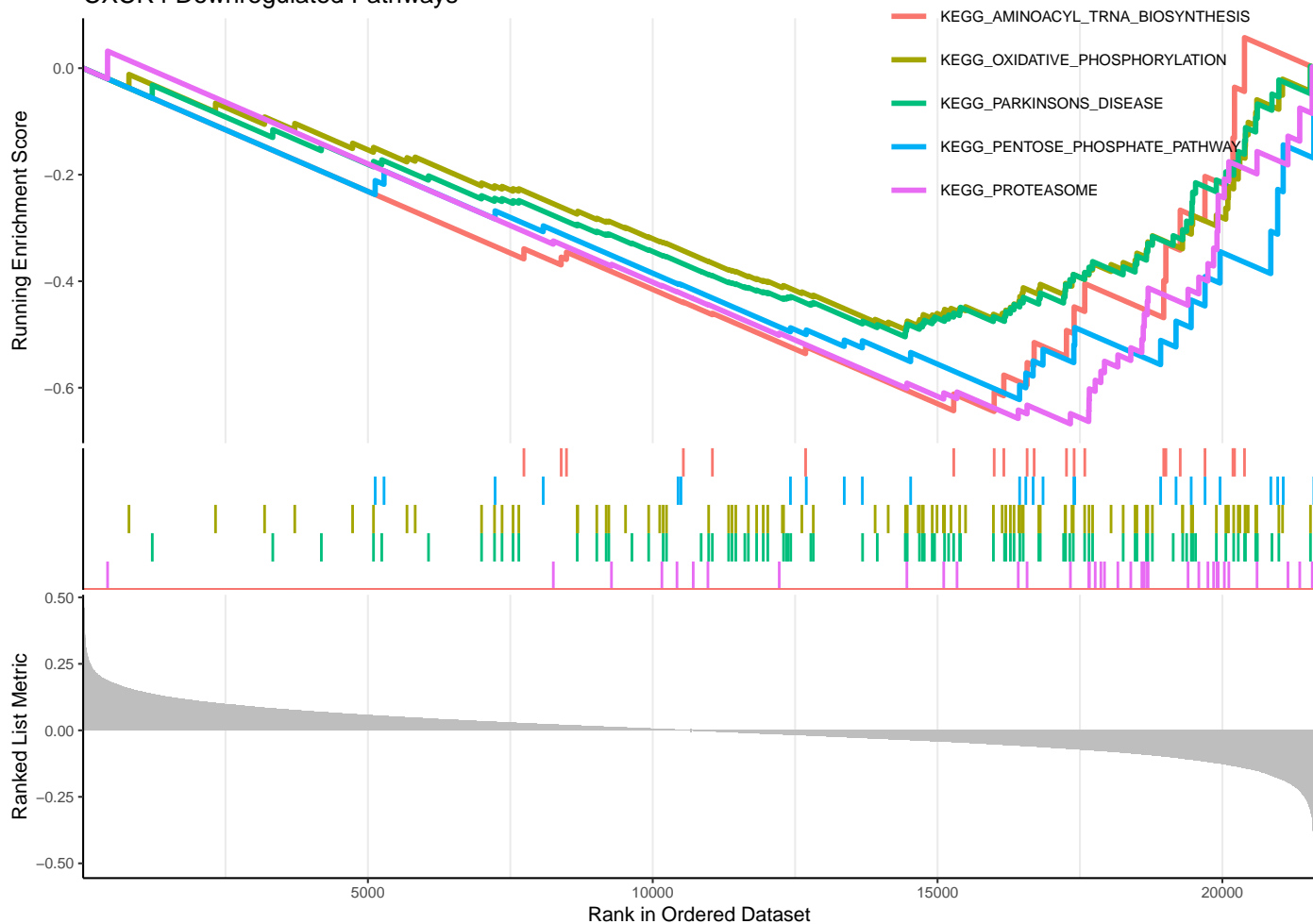

Supplement: Supplementary file 1 [file DataSheet1.zip › 1520845Supplementary files/17筛选核心基因的GSEA通路分析/GSEA_Downregulated.pdf]

# CXCR4 Upregulated Pathways

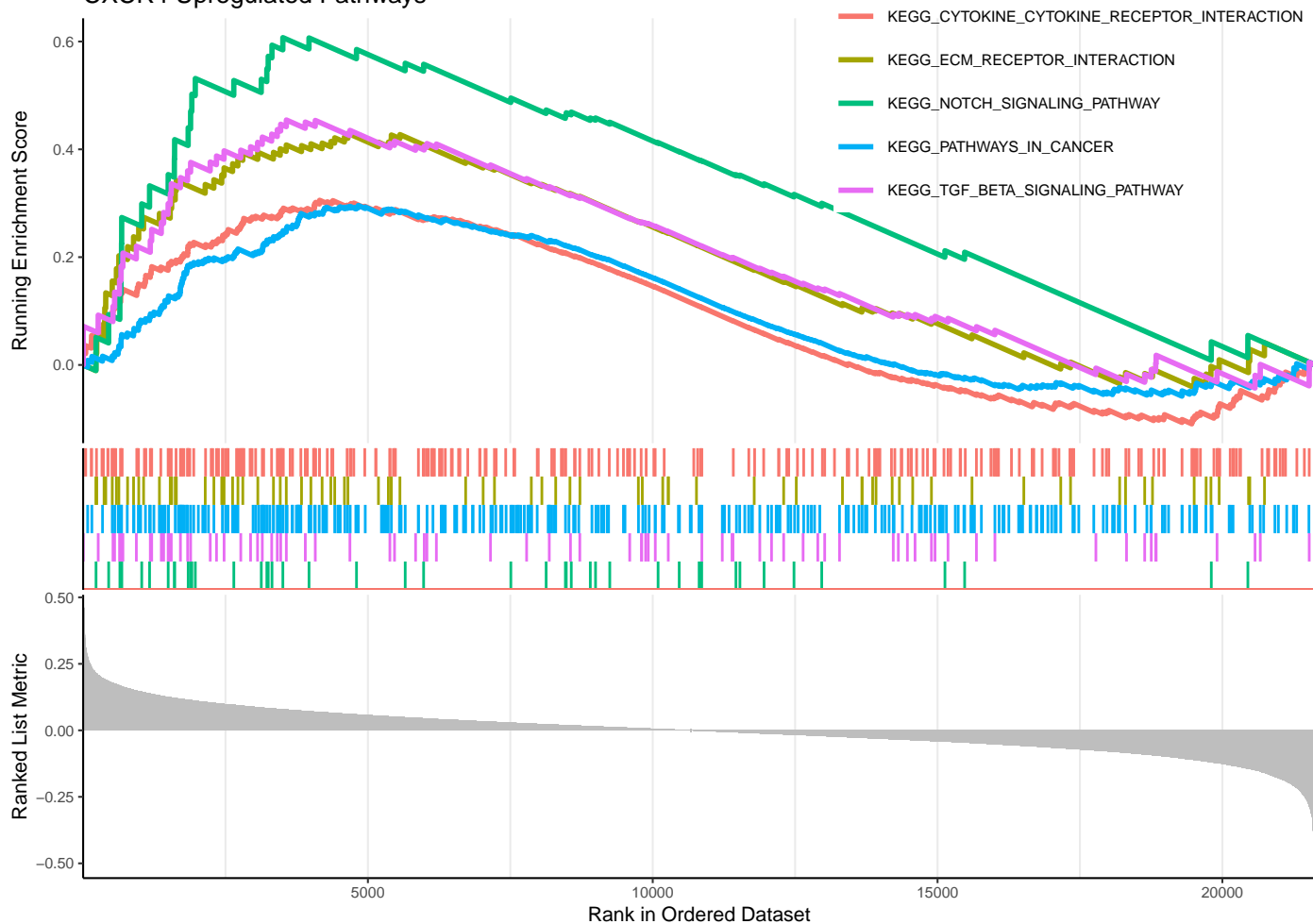

Supplement: Supplementary file 1 [file DataSheet1.zip › 1520845Supplementary files/17筛选核心基因的GSEA通路分析/GSEA_Upregulated.pdf]

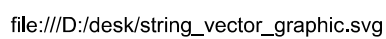

Supplement: Supplementary file 1 [file DataSheet1.zip › 1520845Supplementary files/9差异基因蛋白互作网络string/string_vector_graphic.pdf]
